# *Odf2* haploinsufficiency causes a new type of decapitated and decaudated spermatozoa, Odf2-DDS, in mice

**DOI:** 10.1038/s41598-019-50516-2

**Published:** 2019-10-03

**Authors:** Chizuru Ito, Hidenori Akutsu, Ryoji Yao, Keiichi Yoshida, Kenji Yamatoya, Tohru Mutoh, Tsukasa Makino, Kazuhiro Aoyama, Hiroaki Ishikawa, Koshi Kunimoto, Sachiko Tsukita, Tetsuo Noda, Masahide Kikkawa, Kiyotaka Toshimori

**Affiliations:** 1 Department of Functional Anatomy, Reproductive Biology and Medicine, Graduate School of Medicine, Chiba University, Chiba, 260-8670 Japan; 2 Department of Reproductive Medicine, National Research Institute for Child Health and Development, Tokyo, 157-8535 Japan; 3 Department of Cell Biology, Japanese Foundation for Cancer Research (JFCR) Cancer Institute, Tokyo, 135-8550 Japan; 4 Present Address: Next-generation Development Center for Cancer Treatment, Osaka International Cancer Institute, Osaka, 541-8567 Japan; 5 Present Address: Institute for Environmental & Gender-specific Medicine, Juntendo University Graduate School of Medicine, Chiba, 279-0021 Japan; 6 Department of Cell Biology and Anatomy, Graduate School of Medicine, The University of Tokyo, 7-3-1 Hongo Bunkyo-ku, Tokyo, 113-0033 Japan; 7 Materials and Structural Analysis (ex FEI), Thermo Ficher Scientific, Shinagawa Seaside West Tower 1F, 4-12-2 HigashiSinagawa, Shinagawa-ku, Tokyo 140-0002 Japan; 8 Research Center for Ultra-High Voltage Electron Microscopy, Osaka University, 7-1 Mihogaoka, Ibaraki, Osaka 567-0047 Japan; 9 Department of Biochemistry and Biophysics, University of California San Francisco 600 16th St., San Francisco, CA 94143 USA; 10 Department of Pathology, Stanford University School of Medicine, 300 Pasteur Drive, Stanford, CA 94305 USA; 11 Graduate School of Frontier Biosciences and Medicine, Osaka University, Osaka, 565-0871 Japan; 12 Director’s Room, Japanese Foundation for Cancer Research (JFCR) Cancer Institute, Tokyo, 135-8550 Japan; 13 Present Address: Future Medicine Research Center, Chiba University, Chiba, 260-8670 Japan

**Keywords:** Developmental biology, Diseases

## Abstract

Outer dense fibre 2 (Odf2 or ODF2) is a cytoskeletal protein required for flagella (tail)-beating and stability to transport sperm cells from testes to the eggs. There are infertile males, including human patients, who have a high percentage of decapitated and decaudated spermatozoa (DDS), whose semen contains abnormal spermatozoa with tailless heads and headless tails due to head-neck separation. DDS is untreatable in reproductive medicine. We report for the first time a new type of Odf2-DDS in heterozygous mutant *Odf2*^+/−^ mice. *Odf2*^+/−^ males were infertile due to haploinsufficiency caused by heterozygous deletion of the *Odf2* gene, encoding the Odf2 proteins. *Odf2* haploinsufficiency induced sperm neck-midpiece separation, a new type of head-tail separation, leading to the generation of headneck sperm cells or headnecks composed of heads with necks and neckless tails composed of only the main parts of tails. The headnecks were immotile but alive and capable of producing offspring by intracytoplasmic headneck sperm injection (ICSI). The neckless tails were motile and could induce capacitation but had no significant forward motility. Further studies are necessary to show that ICSI in humans, using headneck sperm cells, is viable and could be an alternative for infertile patients suffering from Odf2-DDS.

## Introduction

Morphologically abnormal spermatozoa with tailless heads and headless tails are conventionally termed decapitated and decaudated spermatozoa (DDS). DDS is found upon a screening examination of semen in patients with severe infertility. Light and electron microscopy have confirmed that DDS is caused by head-neck separation between the head and neck or basal plate^1–4^. Two genes related to DDS have been reported: *Odf1*, in studies of *Odf1*-deleted mice^5,6^ and *HOOK1* (Hook microtubule-tethering protein 1), found to have a missense mutation of A to G (p.Q286R)) in infertile patients with teratozoospermia, called DDS syndrome^7^.

Concerning the assessment of human semen, the World Health Organization (WHO)^8^ indicates that tailless heads is the term for free heads with no necks and no centrosomes and that headless tails is the term for free tails or pinheads with no chromatin and no head structures anterior to the basal plate. Because the tailless heads are immotile, implying they are dead, they are not selected for assisted reproductive technology, such as *in vitro* fertilisation and intracytoplasmic sperm injection (ICSI)^9,10^. ICSI is a technique by which a spermatozoon or a sperm head is directly injected into the ooplasm, which is suitable for the treatment of human patients with asthenozoospermia and lacking sperm motility^11^.

**Figure 1:**
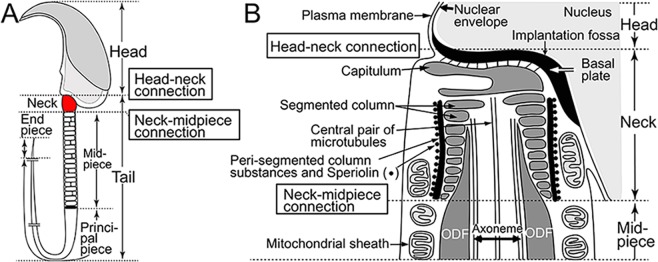
A normal spermatozoon (lateral views). (**A**) Sperm region. (**B**) Neck details. The neck is connected to the head through a basal plate connected to the capitulum. The distal end of the segmented column is connected to the base of the outer dense fibre (ODF). The segmented column is surrounded by the peri-segmented column substances associated with Speriolin.

A spermatozoon is divided into the head and flagellum or tail. The flagellum is divided into the neck or connecting piece, midpiece, principal piece and end piece (Fig. 1). Since the neck construction has complex structures^12^, we focus on two structures: the connection between the head and neck (basal plate) at the implantation fossa, termed the head-neck connection, and the connection between the distal end of the neck (centrosome-derived segmented column) and proximal base of the midpiece (flagellar outer dense fibres), termed the neck-midpiece connection (Fig. 1). The outer dense fibres (ODFs), which are covered by the mitochondrial sheath at the midpiece, surround the axoneme and extend from the distal end of the segmented column, throughout the midpiece, to the distal part of the principal piece. The ODFs are connected to the axoneme^13^, which is localised at the centre of the tail and generally consists of nine peripheral doublet microtubules with central-pair microtubules, giving it is name of the 9 + 2 axoneme. The axoneme 9 + 2 is present in motile cilia, but 9 + 0 in immotile cilia.

The cytoskeletal nature of the outer dense fibre (ODF) is important for the stability and elastic recoil of coordinated flagella-beating and to protect the flagella from shear stress during travelling from the testes to the oocytes in the female reproductive tract^12^. ODF-related proteins or genes and phenotypes are reported as follows. Odf1 and Odf2 interact with each other^14^ and mainly make up the ODFs. Homozygous *Odf1*-knockout (KO) mice lacking the promoter region and exon 1 are infertile due to head-neck separation^5^, and heterozygous males are subfertile due to the weakening of the head-neck connection caused by Odf1 haplodeficiency^6^. Odf2 is present in the outer dense fibres^14–16^.

Odf2 has been reported to play a key role in the formation of immotile primary cilia^17,18^ and motile sperm flagella^19^. Differential splicing of the *Odf2* gene produces Odf2 and cenexin 2, called Odf2/Cenexin; the longer transcript of the *Odf2* gene, Cenexin 2, is crucial for the formation of the centrosome and primary cilia^20^. Odf2 is a putative coiled-coil protein containing two leucine zipper motifs that mediate interaction with itself and microtubules^13^. XL169 ES-derived *Odf2* chimeric males are infertile due to abnormal spermatozoa with bent flagella^19^. Homozygous *Odf2*-knockout (KO) mice lacking *Odf2* exons 6 and 7 or 9 incur centrosome dysfunctions^21,22^. The homozygous conditional *Odf2*-KO (Odf2^ΔΕx6,7/ΔΕx6,7^) mice lacking exons 6 and 7 of the *Odf2* gene, which encodes centrosome-associated protein Odf2/cenexin, have defects in the structure and function of basal bodies in the ciliated tissues, causing primary ciliary dyskinesia, such as coughing in the trachea and early postnatal death by impaired gastrointestinal motility (possibly Hirschsprung’s disease), but the effect on the sperm remains unclear^21^. The genetic model RO072 ES cell-derived *Odf2*-KO mice, in which exon 9 of the *Odf2* gene is targeted, incur embryonic lethality before the embryos reach the blastocyst stage, but the *Odf2*^+/−^ males have no abnormalities^22^. The ODFs contain the enzyme Adenylate Kinase 1 (AK-1) involved in flagella movement^23^ and Tektin4^24^. A cytoskeletal protein, Septin, is present at the distal end of the midpiece and interacts with tubulin and actin^25^.

In this study, we established heterozygous *Odf2*^+/−^ mice using 129 ES-derived *Odf2* chimeric males with deletion of exons 6 and 7 of the *Odf2* gene as reported previously (Fig. S1)^18,21^. We report a new type of decapitated and decaudated spermatozoa caused by *Odf2* haploinsufficiency, termed Odf2-DDS. *Odf2* is indispensable for the neck-midpiece connection composed of a centrosome-derived component and a flagellar component.

## Results

### Characterisation of the separated neck after head-neck separation or DDS

**Figure 2:**
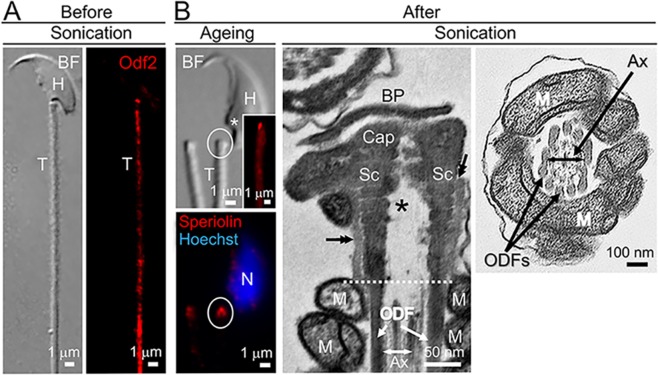
Characterisation of head-neck separation (wild-type). BF: brightfield. IF: immunofluorescence. (**A**) Before separation. (*Left*) BF. (*Right*) IF microscopy with the anti-Odf2 antibody (red). The tail (T) is connected to the head (H) and is positive for Odf2. (**B**) After separation. Separation occurs at the base of the head (*). (*Left*) Ageing. IF microscopy. Circles indicate the same region. T indicates the top of the tail. (*Top Left*) The tail is positive for Odf2 (red) (inset). (*Bottom Left*) The top of the tail is positive for Speriolin (red). Blue (Hoechst 33258): nucleus. (*Middle* and *Right*) Sonication. Transmission electron micrographs. (*Middle*) The proximal end of the tail contains the basal plate (BP) connected to the capitulum (Cap), segmented columns (Sc) connected to the outer dense fibres at the distal end (dotted line), the axoneme and peri-segmented column substances (double arrows). The white dotted line indicates the approximate position of the neck-midpiece connection. (*Right*) Normal arrangement of the tail components in the midpiece (cross-section). The total number of spermatozoa examined was 200 in each case, except it was 50 for transmission EM. N = 5 (different males). Ax: axoneme. H: head. M: mitochondria. N: nucleus.

To precisely compare the status of sperm neck components in DDS with that in Odf2-DDS (described later), we first examined the localisation of γ-Tubulin and Speriolin in separated sperm cells after natural ageing and artificial sonication in wild-type (Odf2^+/+^) males. Immunofluorescence (IF) microscopy showed that the Odf2 immunostaining was always positive in the tail before (Fig. 2A) and after head-tail separation (Fig. 2B) and that the dot-shaped immunostaining for Speriolin was always localised at the most proximal end or top of the separated tail (Fig. 2B). Since γ-Tubulin and Speriolin are reported to be present in the centrosome of the neck^18,26^, double-positive immunostaining for γ-Tubulin and Speriolin was evidence for identifying the neck. Transmission electron microscopy confirmed that head-neck separation occurred at the implantation fossa between the head and neck or basal plate and that the separated tail contained the neck components (basal plate, capitulum, and segmented columns), the proximal part of the axoneme, and other parts of the tail (Fig. 2B); these findings were consistent with those of DDS^1–4^. An electron-dense substance surrounded the segmented column, consistent with a previous report^26^, and was always observed at the most proximal part of the tail before (Figs 1B and S2,A) and after head-neck separation (Fig. 2B). Hereafter, we call the electron-dense substance associated with Speriolin (Fig. S2B) the peri-segmented column substance (Fig. 1B). The presence of the peri-segmented column substance and/or Speriolin is evidence for identifying the neck.

### Characterisation of the Odf2 chimeric neck-midpiece separation

The epididymal fluid recovered from the Odf2 chimeric cauda epididymides contained many morphologically abnormal spermatozoa: heads, heads with processes projected from the ventral base (which are later called headnecks), and heads with fractured necks (Fig. S3A). Compared with those in the wild-type, the percentages of the morphologically abnormal spermatozoa and heads with processes in the abnormal spermatozoa were higher in the chimaera (Fig. S3B). The processes were always projected from the ventral base and were approximately 1 μm in both width and length (Fig. S3C). IF microscopy showed that the processes were double-positive for Speriolin and γ-Tubulin and that the separated tails were positive for Odf2 (Fig. S3C). Transmission EM showed that the process contained neck components (basal plate, capitulum, and segmented column) but lacked the complex of outer dense fibres and axonemal microtubule doublets (Fig. S3D). Since all these results indicated the nature of the sperm neck, except for the complex of outer dense fibres and axonemal microtubule doublets, we call heads with processes “headnecks”. Almost all the separated tails were motile, but the swimming pattern was lethargic with no significant forward motility. Since these findings were similar to those of the *Odf2*^+/−^ spermatozoa, the data are shown later in the section on the *Odf2*^+/−^ spermatozoa. The embryonic development to the 2-cell and blastocyst stages was not significantly different between the wild-type and chimaera, although the development was greatly perturbed in the chimaera (Fig. S3E). Based on these lines of evidence, we assumed that the headnecks would be viable and transmit the targeted allele of the chimeric males to the offspring by intracytoplasmic headneck injection.

### Transmission of the targeted allele of the chimeric males to offspring of *Odf2*^+/−^ males

**Figure 3:**
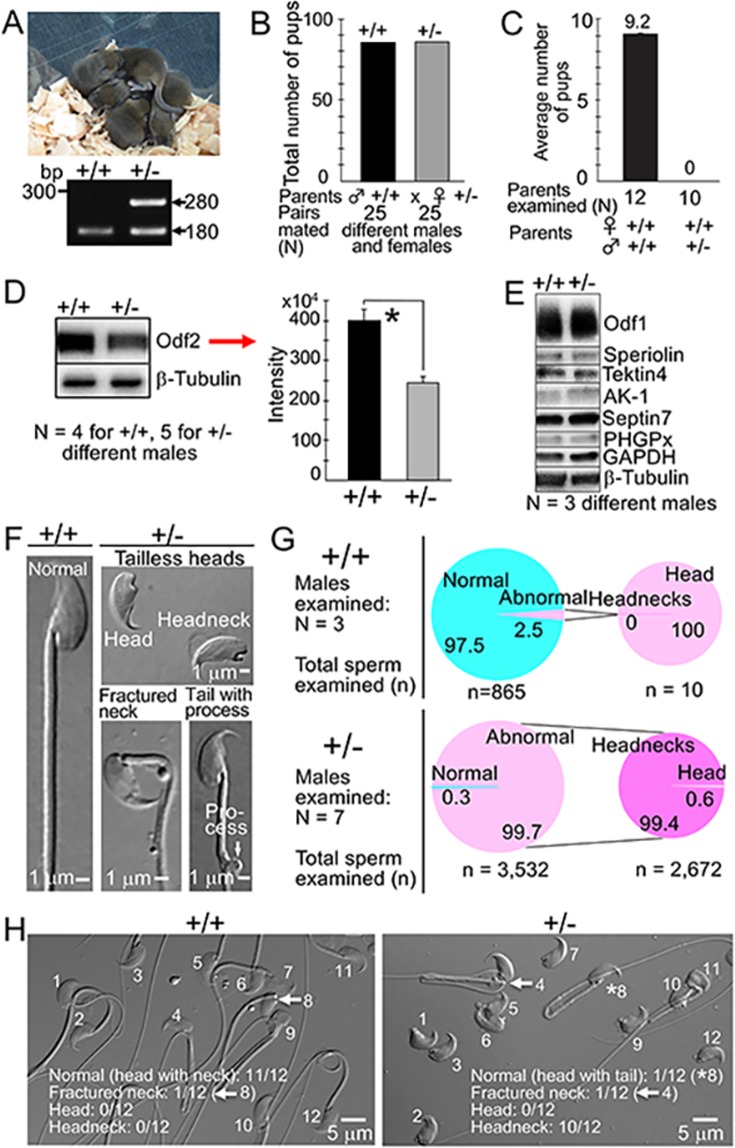
Characterisation of *Odf2*^+/−^ males. (**A**) (*Top*) Agouti coat-coloured pups produced by intracytoplasmic headneck injection. (*Bottom*) Genotyping. +/+: Wild-type (180). +/−: Heterozygous *Odf2*^+/−^ (180 and 280). (**B**) Pup production of heterozygous *Odf2*^+/−^ (+/−) females is normal. (**C**) Pup production of heterozygous *Odf2*^+/−^ (+/−) males is low (0) compared to that of wild-type (+/+) (9.2). (**D**) (*Left*) Western blotting. (*Top*) Reduced amount of the 75 kDa Odf2 protein in *Odf2*^+/−^ (+/−) compared to that in *Odf2*^+/+^ (+/+). Full-length membranes (CBB staining and western blotting); all data are presented in Fig. S4. (*Bottom*) β-Tubulin; internal control. The same amount of protein (0.5 μg/lane) was loaded. (*Right*) The reduction is quantitatively significant (*P < 0.05). The immunostaining intensity obtained by western blotting (*Left* image) was subjected to analysis with Image Lab Software in a Bio-Rad ChemiDoc XRS+ (molecular imager). The mean ± SEM is 243 × 10^4^ ± 16 × 10^4^ in Odf2^+/−^ versus 400 × 10^4^ ± 28 × 10^4^ in Odf2^+/+^. (**E**) The display shows the western blots for the midpiece-related proteins cropped from the same or different membranes. Full-length membranes (CBB staining and western blotting) for all samples are presented in Fig. S5. No marked difference in the midpiece-related proteins between the wild-type (+/+) (*Left*) and heterozygotes (+/−) (*Right*). β-Tubulin was used as an internal control. The same amount of protein per lane was loaded for Odf1 (1 μg), Speriolin (3 μg), Tektin4 (16 μg), AK-1 (16 μg), Septin7 (6 μg), PHGPx (16 μg), GAPDH (16 μg) and β-Tubulin (16 μg). (**F**) Classification of sperm morphology (brightfield). (**G**) Percentages (%) of abnormal spermatozoa (*Left*; large pie chart) and headnecks among abnormal spermatozoa (*Right*; small pie chart) are high in *Odf2*^+/−^ (+/−) (99.7% and 99.4%, respectively) compared to those in *Odf2*^+/+^ (+/+) (2.5% and 0%, respectively). The total number of samples examined is written at the bottom of the pie charts. (**H**) Representative images at low magnification (brightfield) of *Odf2*^+/+^ (+/+) and *Odf2*^+/−^ (+/−) spermatozoa. Numerals (1–12) in each figure are sperm number. The proportion of headnecks shown in these figures is well in accord with that of *G* (*Right*).

Intracytoplasmic headneck injection produced F0 offspring: two heterozygous *Odf2*-KO (*Odf2*^+/−^) females, two *Odf2*^+/+^ males and one *Odf2*^+/+^ female (Fig. 3A). The two F0 *Odf2*^+/−^ females were fertile and produced *Odf2*^*+/+*^ and *Odf2*^+/−^ pups at a similar ratio to Mendel’s laws of heredity after pairing with *Odf2*^*+/+*^ males (Fig. 3B). Therefore, we maintained the *Odf2*^+/−^ mouse line by natural mating of the *Odf2*^+/−^ females with *Odf2*^+/+^ C57BL/6JJmsSlc (B6) males. On the other hand, the *Odf2*^+/−^ males were infertile (Fig. 3C). When we examined the amount of Odf2 protein in the spermatozoa recovered from *Odf2*^+/−^ cauda epididymides by western blotting, the amount of the 75 kDa Odf2 protein was significantly reduced in *Odf2*^+/−^ compared with that in *Odf2*^+/+^ males, as confirmed by the quantitative analysis (Figs 3D and S4). By contrast, the immunostaining intensities of other tail-related proteins (Odf1, Speriolin, Tektin4, AK-1, Septin7, PHGPx, GAPDH and β-Tubulin) were not different between the *Odf2*^+/−^ and *Odf2*^+/+^ samples by western blot (Figs 3E and S5). These results led us to conclude that the specific reduction in the amount of the Odf2 protein in *Odf2*^+/−^ spermatozoa is caused by heterozygous deletion of the *Odf2* gene (*Odf2* haploinsufficiency).

**Figure 4:**
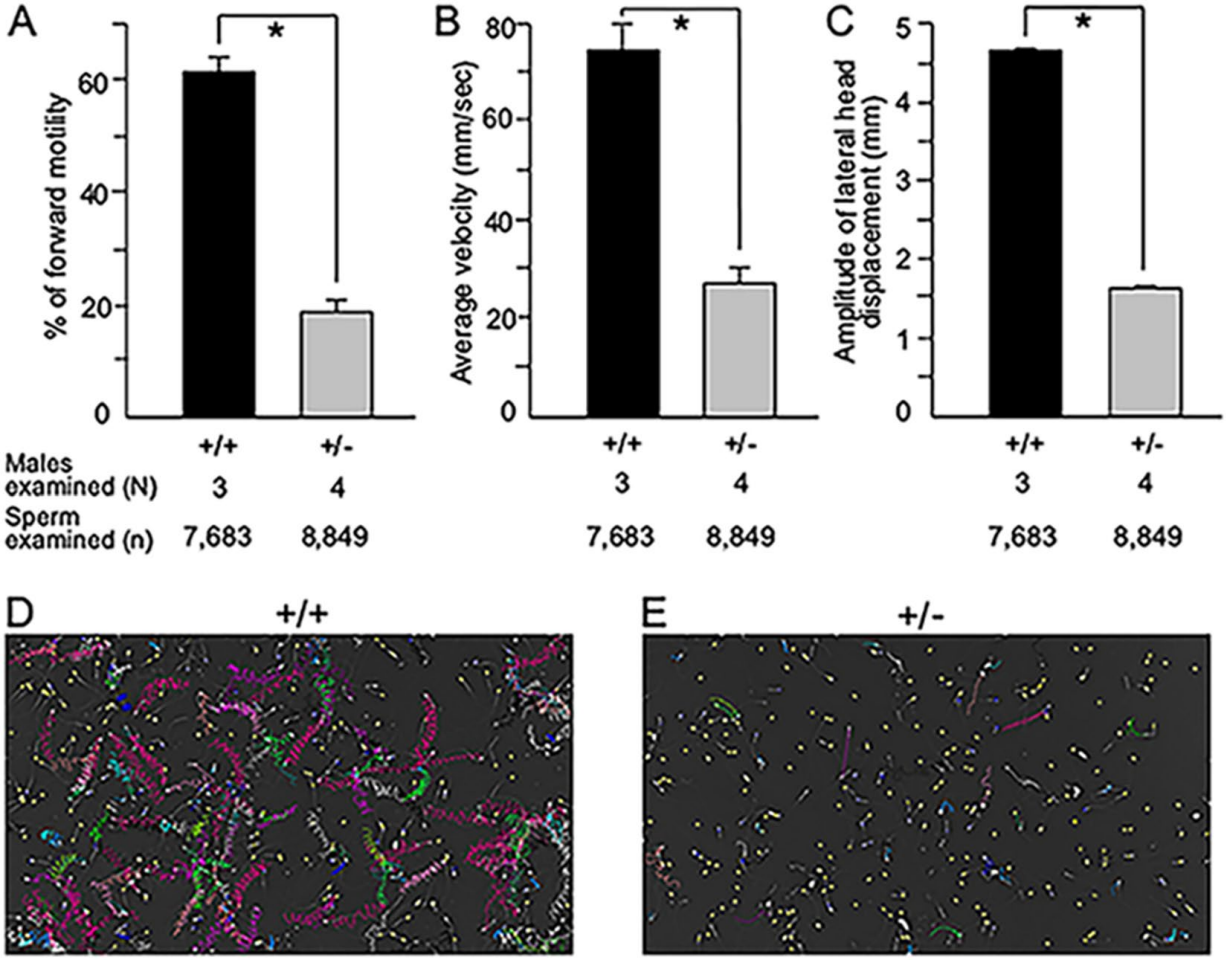
Sperm movement analysed by SMAS *Odf2*^+/−^ (+/−) spermatozoa are poor in movement compared to *Odf2*^+/+^ (+/+) spermatozoa. (**A**) Forward motility. (**B**) Average velocity. (**C**) The amplitude of the lateral head. All these factors are significantly lower (*P < 0.01) in *Odf2*^+/−^ (+/−) than in *Odf2*^+/+^ (+/+). The total numbers of spermatozoa and males examined are written at the bottom of the figures. Representative tracts: optimal motility of *Odf2*^+/+^ (+/+) spermatozoa (**D**) and poor motility of *Odf2*^+/−^ (+/−) spermatozoa (**E**). The total numbers of males and spermatozoa examined are written at the bottom of the figures.

*Odf2*^+/−^ epididymal fluid contained many morphologically abnormal spermatozoa, as was the case for the chimaera. The abnormalities were classified into three groups: heads, spermatozoa with fractured necks, and spermatozoa with a lateral process at the distal end of the midpiece (Fig. 3F). Later, the heads were further classified into headnecks and heads (Fig. 3G). *Odf2*^+/−^ epididymal fluid contained 99.7% abnormal spermatozoa, which was higher than the 2.5% in the *Odf2*^+/+^ epididymal fluid. The proportion of headnecks in the abnormal spermatozoa was significantly higher in the *Odf2*^+/−^ (99.4%) than in the wild-type (0%). The *Odf2*^+/−^ headnecks were immotile; however, the neck-fractured spermatozoa and neckless tails were motile, though the movement was lethargic with no significant forward motility (Fig. 4 and Movie S1), implying that the separated tails could not transport the paternal genome to the egg but that the headnecks were alive.

### Characterisation of the *Odf2*^+/−^ spermatozoa caused by neck-midpiece separation (Odf2-DDS)

**Figure 5:**
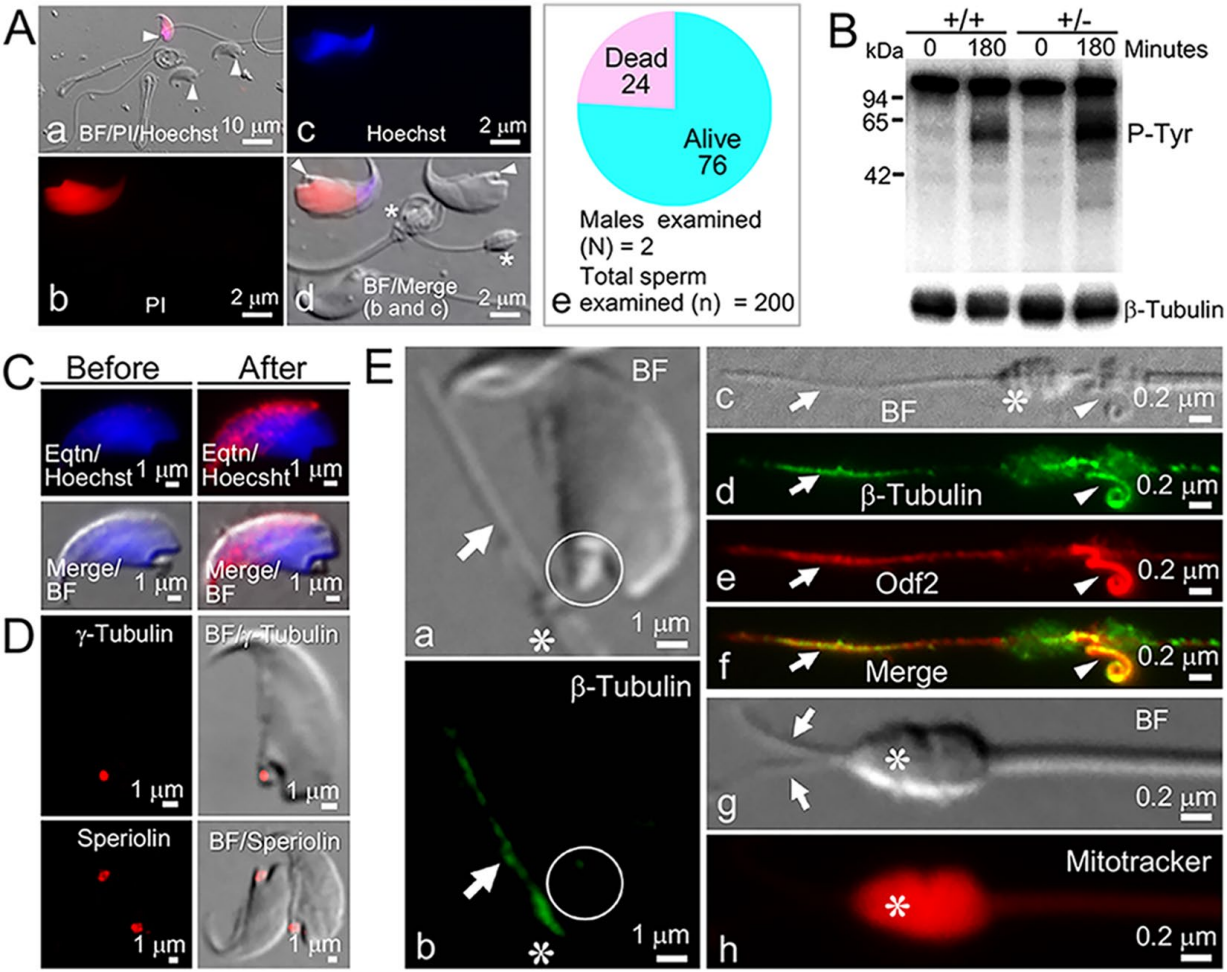
Characterisation of *Odf2*^+/−^ spermatozoa. BF: brightfield. IF: immunofluorescence. (**A**) IF microscopy. PI-positive (red) and 33258-positive (blue) heads are dead. PI-negative and Hoechst-negative heads are alive. (*a*) Low magnification of headnecks (arrowheads). (*b*–*d*) High magnification of different areas of *a*. (*d*) Arrowheads and asterisks (*) indicate the necks of headnecks and the barrel-shaped structure, respectively. (*e*) Three-fourths of *Odf2*^+/−^ spermatozoa (mostly headnecks) are alive. (**B**) The display shows increased tyrosine phosphorylation (p-Tyr) in the western blots for *Odf2*^+/−^ (+/−) and control (+/−) after capacitation (180). The images were cropped from the same membranes. Full-length membranes for CBB staining and western blotting are presented in Fig. S6. Spermatozoa were incubated in TYH medium for 180 minute and then treated with the anti-phosphotyrosine antibody for western blotting. β-Tubulin; internal control. The same number of sperm tails (7 × 10^6^/lane) was loaded. (**C**) IF microscopy. (*Right*) Acrosome reaction of the headnecks: MN9-positive (red). (*Left*) Before the acrosome reaction: MN9-negative. Spermatozoa (headnecks) were incubated in TYH medium for 180 minute. The acrosome reaction was monitored with the anti-acrosome antibody MN9 (red), without detergent treatment. Blue (Hoechst): nucleus. (**D**) IF microscopy for γ-Tubulin (*Top*: red) and Speriolin (*Bottom*: red) in headnecks with anti-γ-Tubulin and anti-Speriolin antibodies, respectively. (**E**) IF microscopy. (*a* and *b*) Anti-β-Tubulin (green) antibody. Arrows and white circles indicate the β-Tubulin-positive rod-shaped structure (green) projected from the separated tail and the β-Tubulin-negative headneck, respectively. An asterisk indicates the top of the tail. (*c*-*f*) Anti-β-Tubulin (green) and anti-Odf2 (red) antibodies. Arrows (β-Tubulin-positive) and arrowheads (Odf2-positive) indicate the rod-shaped structure and the whirl-shaped structure, respectively. An asterisk indicates a barrel-shaped structure. (*g* and *h*) Arrows indicate two rod-shaped structures projected from the top of the tail, near a barrel-shaped structure (asterisks) stained red due to the presence of mitochondria by MitoTracker after Triton X-100 treatment. N = 3 different males for *A*-*E*. The total number of spermatozoa examined was at least 100 from 2 different males for (**A,C–E**).

We examined whether the *Odf2*^+/−^ headnecks were alive or dead using propidium iodide (PI) and Hoechst 33258 (Hoechst). Seventy-six percent of the headnecks were PI-negative and Hoechst-negative (Fig. 5A), indicating that the headnecks were alive. The remaining 24% of the headnecks were PI-positive and Hoechst-positive, indicating death. All the *Odf2*^*+/+*^ heads by natural ageing were also PI-positive and Hoechst-positive, indicating death. Interestingly, *Odf2*^+/−^ spermatozoa, mostly composed of neckless tails, induced capacitation as shown by western blotting with an anti-phosphotyrosine antibody, a marker for capacitation^27^ (Figs 5B and S6). In addition, the headnecks induced the acrosome reaction, as shown by IF microscopy with the anti-acrosomal antibody MN9, a marker for the acrosome reaction^28^ (Fig. 5C). IF microscopy showed that the headnecks were positive for γ-Tubulin and Speriolin (Fig. 5D) and that the tails were positive for Odf2 (Fig. 5E *a* and *b*). In addition, we found two other interesting structures: one was a fine rod-shaped structure (0.1–0.5 μm in diameter) projected from the most proximal end of the separated tail, and the other was a barrel-shaped structure localised near the top of the separated tails. IF microscopy showed that the former was double-positive for β-Tubulin and Odf2 (Fig. 5E *c–h*), and the latter was positive for MitoTracker, a marker for mitochondria (Fig. 5E *g* and *h*). These two structures were further analysed by TEM in the next section. Another prominent structure, a whirl-shaped structure localised at the distal midpiece, was not analysed further because it did not directly relate to the purpose of this study (analysis of the neck-midpiece separation).

**Figure 6:**
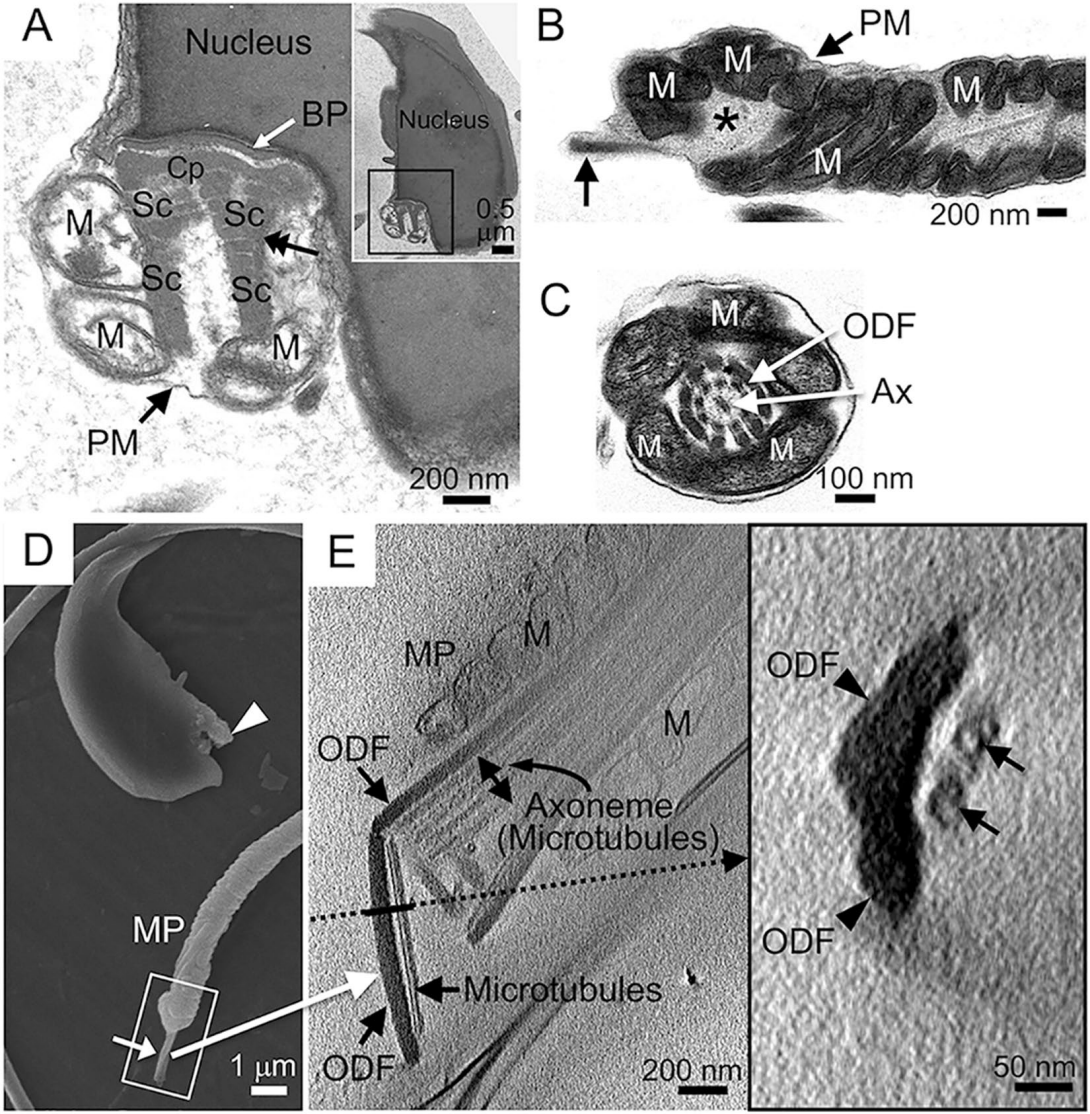
*Odf2*^+/−^ headnecks and neckless tails revealed by electron microscopy (EM). (**A–C**) Transmission EM. (**D**) Scanning EM. (**E**) Scanning transmission EM (STEM). (**A**) The neck region of the headneck is magnified from the rectangular area of the inset, where the neck components are found; basal plate (BP), capitulum (Cp), segmented column (Sc), mitochondria (M) and peri-segmented column substances (double arrows). The complex of outer dense fibres and axonemal microtubules is absent; the absence was further proven by a Z-stack (44 photographs with 5 nm thickness/photograph; 155.8 MB) obtained by Cryo-STEM. The images show the whole region (approximately 200 nm in width) where the segmented columns are localised. These Z-stack images are provided as additional Supplementary Images. (**B**) An arrow indicates a rod-shaped structure (approximately 150 nm in diameter) projected from the proximal end of the separated tail, near the disarranged mitochondria in the barrel-shaped structure (*). (**C**) Rather normally arranged outer dense fibres and axoneme found in a more distal region of the midpiece (cross-section). (**D**) A headneck (arrowhead) and a rod-shaped structure projected from the proximal end of the separated tail (arrow). Similar rod-shaped structures (large white arrow, rectangle) were further examined by cryo-STEM (**E**). (**E**) (*Left*) Three-dimensional tomography created after taking images by STEM. This area is further magnified in Fig. S8B for better observation. (*Right*; inset) The transverse cross-section along the thick line indicated in the left image. The rod-shaped structure consists of a complex of outer dense fibres (ODF) and microtubule doublets (arrows), which are closely associated. The same region but at a different angle is magnified in Fig. S8B. Ax: axoneme. M: mitochondria. MP: midpiece. ODF: outer dense fibre. PM: plasma membrane. (**A–D**) The experiments were performed in duplicate in 2 different males (N = 2).

TEM demonstrated that the neck localised at the base of the headneck by light microscopy contained a basal plate, capitulum, a segmented column, peri-segmented column substances and neck mitochondria, all of which were enveloped by the plasma membrane (Fig. 6A). However, the complex of outer dense fibres and axonemes was not detected in the *Odf2*^+/−^ headneck or in the chimeric headneck. In accordance with this result, immunogold particles from immunoelectron microscopy with the anti-Odf2 antibody conjugated to immunogold particles were absent in the *Odf2*^+/−^ headneck (Fig. S7A,B), while the immunogold particles were rich in the outer dense fibres at the midpiece, which could be used as an internal control (Fig. S7B). Transmission EM and scanning EM showed that the fine rod-shaped structure projected from the tail top was electron-dense (Fig. 6B) and bright (Figs 6D and S8A), respectively. Severe disarrangements of the outer dense fibres and axonemes were found in the barrel-shaped structure of the separated midpiece (Fig. 6B), but the normal-looking arrangement of the outer dense fibres and axonemes was also observed in other midpieces (Fig. 6C). Since it was difficult to recognise the components of the fine rod-shaped structure by conventional EM, we confirmed it by cryo-STEM (scanning transmission electron microscopy) tomography. As a result, the fine rod-shaped structure consisted of the complex of outer dense fibres and axonemal microtubules; they were closely associated with each other (Figs 6E and S8B).

Testicular cross-sections stained with toluidine blue showed normally proceeding spermatogenesis in the *Odf2*^+/−^ testis (Fig. S9A), indicating that the germ cell division was not disturbed. Although we did not detect neck-midpiece separation in the thin sections, we detected it in the sperm cells freed from the *Odf2*^+/−^ seminiferous tubules (Fig. S9B).

**Figure 7:**
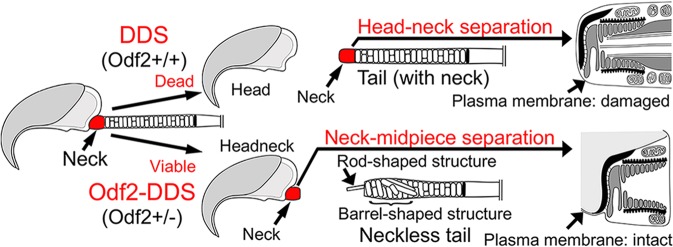
Diagram of the differences between decapitated and decaudated spermatozoa (DDS) and Odf2-DDS. (*Top*) DDS; head-neck separation. DDS is untreatable due to sperm death. The degenerated tail could contain the neck components, with the overlying plasma membrane damaged (Fig. 1). (*Bottom*) Odf2-DDS; neck-midpiece separation. Odf2-DDS is treatable by intracytoplasmic headneck injection or ICSI, since the headneck contains a healthy paternal genome with the overlying intact plasma membrane. The separated headneck lacks the complex of outer dense fibres and microtubules because they have slipped out from the neck; the complex is found as a rod-shaped structure(s) at the top of the separated tail, near a barrel-shaped structure.

The differences between conventional DDS and this new Odf2-DDS are summarised in Fig. 7 and Table 1.

**Table 1:** Differences between decapitated and decaudated spermatozoa (DDS) and Odf2-DDS.

Type of DDS	Status of head	Head-neck	Neck-midpiece	Status of tail
Conventional DDS and DDS syndrome	Propidium iodide positive, without neck Tailless head	Disconnected	Connected	Immotile, with neck Headless tail
Odf2-DDS	Propidium iodide negative, with neck, Headneck paternal genome, γ-Tubulin and Speriolin	Connected	Disconnected	Motile, without neck Neckless tail

## Discussion

Male infertility was attributed to *Odf2* haploinsufficiency in spermatozoa, which then disrupted the connection between the centrosome-derived segmented column and flagellar outer dense fibres and caused neck-midpiece separation. Such a disruption has not been reported until now. Since *Odf2* is specifically expressed in male germ cells to start the production of the encoded Odf2 protein in round (step 5) spermatids^29,30^, it is rational that neck-midpiece separation occurred during spermiogenesis. The *Odf2*-deficient headnecks were viable and entirely enclosed by the plasma membrane.

We have shown that heterozygous *Odf2*^+/−^ males and females were healthy and grew well and that the normal spermatogenesis proceeded in the males, indicating that the heterozygous deletion of the *Odf2* gene does not affect centrosome function. The phenotype of the *Odf2*^+/−^ males as well as the chimaera was also different from that of XL169 ES-derived *Odf2* chimeric males, which have abnormal spermatozoa with bent flagella^19^. Although the exact reason for the difference is unclear, there is a difference between the two studies. Exons 6 and 7 of the *Odf2* gene were deleted in this study, while the XL169 ES-derived *Odf2* chimeric males were made by inserting the vector pGT0Lxf between exons 4 and 5 of the *Odf2* gene (gene trap XL169, Bay Genomics). Since this strategy predicts deletion of the leucine zipper that mediates binding to Odf1, as shown by Mouse Genome Informatics data, the amount of Odf2 protein produced by the chimeric sperm may not be reduced.

The decrease in the Odf2 protein amount in Odf2-DDS sperm cells will weaken the binding ability of the outer dense fibres to the segmented columns, leading to neck-midpiece separation. On the other hand, the complex of outer dense fibres and microtubules remained engaged in the separated tails. Since the Odf2 protein was reported to interact with microtubules^13^, it is possible that sub-nanometre level failure(s) might occur in the tail. For example, the outer dense fibres might fail to bind mitochondria because mitochondrial disarrangements frequently happen in the barrel-shaped structure near the top of the separated necks. *Odf2* haploinsufficiency appears not to affect the other midpiece-related proteins, because the western blot for Odf1, Speriolin, Tektin4, AK-1, Septin7, PHGPx, GAPDH and β-Tubulin was not different in the *Odf2*^+/−^ spermatozoa.

As summarised in Table 1, the most significant difference between Odf2-DDS and DDS, including DDS syndrome, was the highly viable headneck carrying the paternal genome with a motile tail *versus* the dead head with an immotile tail. The Odf2 chimeric headneck injected into oocytes was able to develop into a blastocyst and transmit the paternal genome to offspring by intracytoplasmic headneck injection. By contrast, the heads produced in DDS are unavailable for assisted reproductive technology as recommended by the WHO. Historically, the decapitated or decaudated sperm defects were first reported in infertile bulls^31^. Since then, approximately 10 mammalian DDS-related studies have been reported. All reported cases were caused by head-neck separation^2–5,7,32–37^. DDS syndrome in two brothers was thought to be of genetic origin, but the details were unclear^3^. The genes responsible for DDS were not reported in some of the 10 studies^2^, though the authors thought the cases were of genetic origin, and the genes were unknown in two other cases. Gene mutations that cause head-neck separation in rodents are *hd* (hypodactylous) mutation^37^, *OAZ-t* (ornithine decarboxylase antizyme) deletion^38^ and *KSR2* (Kinase suppressor of Ras 2) deletion^39^, but neck-midpiece separation has not been reported. Thus, the nature of Odf2-DDS differs from that of other mammalian DDS.

Until now, Odf2-DDS had been discovered in neither animals nor humans. There may be several reasons. First, it is quite difficult to diagnose the neck-midpiece separation upon the initial screening examination of semen because the immotile Odf2-DDS sperm cells or headnecks are likely thought to be dead cells and because the impairments are at the subcellular and biochemical levels. In fact, it was difficult for us to recognise that the processes (approximately 1 μm in width and length) of the headnecks are the sperm necks themselves and are caused by neck-midpiece separation. It was also difficult to understand that the rod-shaped structure (approximately 150 nm in diameter) projected from the proximal end of the separated tail consisted of a complex of outer dense fibres and microtubule doublets. In addition, information concerning the *Odf2* gene, including haploinsufficiency and head-tail separation, is limited, although some things are known about it, as follows. All chimeric males that had sperm cell-related haploinsufficiency (affected gene(s): *Prm1* and *Prm2*^40^, *Cyp17*^41^, *Klhl10*^42^, *Spag16/Pf20*^43^, *hnRNPG-T*^44^, and *Tssk1* and *Tssk2*^45^) did not transmit the targeted allele to the offspring; thus, none of their pathologies were related to head-tail separation. Only two genes related to DDS have recently been reported: mouse *Odf1*^5,6^ and human *HOOK1*^7^. Two studies relating to the *Odf2* gene were also recently reported, as described in the Introduction. One was on homozygous conditional *Odf2*-KO mice with exons 6 and 7 targeted^21^, in which the effect on the sperm remained unclear. The other was on gene-trap KO mice with exon 9 targeted^22^, in which *Odf2*^+/−^ males had no abnormalities.

However, Odf2-DDS could be found in the future, as infertility due to asthenoteratozoospermia is reported to be mainly caused by impaired development of the outer dense fibres^46^, and as the amino acid sequences of human and mouse ODF2 are almost identical^47^. In addition, three patients, whose conditions were thought to be of genetic origin, contained numerous motile acephalic sperm (headless tails)^35^ as shown in this study, in which headless tails as well as tail-less head were abundant. Patients with severe infertility due to Odf2 haploinsufficiency can survive to adulthood and will be diagnosed with a type of teratozoospermia.

## Conclusions

We have shown the nature of a new type of Odf2-DDS due to *Odf2* haploinsufficiency. Figure 7 shows the differences in the characteristic structures between DDS and Odf2-DDS. The head-tail separation occurs at the neck-midpiece connection in Odf2-DDS, leading to the generation of headneck sperm cells, which are completely covered by intact plasma membranes. In contrast, it occurs at the head-neck connection in DDS, leading to the generation of head-only sperm cells, which are damaged or dead. The headnecks are immotile but alive, have neck proteins, and are capable of producing offspring by ICSI. ICSI using human headneck sperm cells could be an alternative for infertile patients suffering from Odf2-DDS. Further studies are necessary to prove that ICSI using headneck sperm cells can produce viable embryos in humans.

## Methods

### Animals

Animal experiments using C57BL/6JJmsSlc (B6), B6D2F1 (BDF1) and ICR mice (Japan SLC and CLEA) were conducted according to the Declaration of Helsinki, and protocols approved by Chiba University.

### Antibodies

The antibodies and chemicals used are described in Table S1.

### Generation of Odf2 mutant mice

Exons 6 and 7 of the *Odf*2 gene were disrupted (Fig. S1). The targeting vector was transfected into 129/Sv embryonic stem (ES) cells, and the recombinant cells were injected into blastocysts derived from B6 mice. The blastocysts were transplanted into pseudopregnant ICR female mice to make chimeric mice. Germline transmission was confirmed by PCR amplification using tail DNA from the progeny. The genotyping primers for PCR were (5′-3′) CGGAATGAAAGGGGACACCGTGAATGTACG (forward; Primer 1), AAGTCTGAGGCCTGCTAGAATCTGACTGAC (reverse; Primer 3) and CCTCCGCAAACTCCTATTTCTG AG (reverse; Primer 2).

### SDS-PAGE and western blot

The methods were as reported previously^28^ with some modifications. For Odf2, Odf1, Speriolin, Tektin4 and β-Tubulin, spermatozoa recovered from cauda epididymides or testicular germ cells for Speriolin were washed with PBS (phosphate- buffered saline) and centrifuged. The pellets were lysed in lysis buffer containing 2% Triton X-100 and 5 mM DTT on ice for 15 minute and lysed in a buffer containing 6 M urea, 5 mM DTT and protease inhibitor cocktail [0.5 mmol/L 4-(2-aminoethyl)benzenesulfonyl fluoride hydrochloride, 0.15 μmol/L aprotinin, 1 μmol/L E-64, 1 μmol/L leupeptin hemisulfate monohydrate, 0.5 mmol/L disodium edetate dihydrate (EDTA)] (Nacalai) on ice for 20 hours. To precisely compare the amount of sperm proteins, protein concentrations were measured, referring to the control reference, and bovine serum albumin (0–2 mg/ml) was measured by a Multi-Mode Microplate Reader (Molecular Device), using the Pierce 660 nm Protein Assay Kit (Pierce). The lysates were mixed in SDS sample buffer (4% SDS, 20% glycerin, 0.01% BPB, 10% 2-ME, 0.125 M Tris-HCl) (Nacalai). For AK-1, Septin7, PHGPx, GAPDH and β-Tubulin, sperm pellets collected as indicated above were lysed in RIPA buffer containing 50 mmol/L Tris-HCl (pH 7.6), 150 mmol/L NaCl, 1% Nonidet P 40, 0.5% sodium deoxycholate, 0.1% sodium dodecyl sulfate, and protease inhibitor cocktail (Nacalai). Protein concentration was measured using the Microplate Reader with the BCA (bicinchoninic acid) protein assay kit (Nacalai). The lysates were mixed in SDS sample buffer (Nacalai). For the capacitation experiment, sperm samples were prepared as reported previously^28^, resuspended to a final concentration (5 × 10^3^ cells/μl) by counting tails and capacitated in IVF dishes in a humidified atmosphere of 5% (v/v) CO_2_ in air at 37 °C in a CO_2_ incubator. Spermatozoa before capacitation (0 minute) and after capacitation (180 minute) were washed with PBS, centrifuged, lysed in SDS-sample buffer (Nacalai) at a final concentration of 7 × 10^4^ cells/μl, and separated by SDS-PAGE followed by western blotting with anti-phosphotyrosine antibody. All samples, except urea-treated samples, were boiled at 98 °C for 5 minute prior to running the gels. The separation was done in 10% or 12% acrylamide Mini-PROTEAN TGX^®^ Precast Gels equipped in Mini-PROTEAN 3 Cell^®^ (Bio-Rad) chambers. For western blotting, proteins separated by SDS-PAGE were transferred to polyvinylidene difluoride (PVDF) membranes using the transfer pack for the Trans-Blot Turbo system (Bio-Rad). The proteins on the PVDF membranes were visualised with Coomassie Brilliant Blue (CBB) Stain One^®^ (Nacalai), destained with the Rapid CBB Destain Kit^®^ (Nacalai), and soaked in Blocking One^®^ (Nacalai) buffer for 30 minute to suppress nonspecific background. The blots were incubated with primary antibodies at room temperature for 3 hours, incubated with HRP-conjugated secondary antibodies for 1 hour and developed with the Clarity Western ECL Substrate^®^ (Bio-Rad). The signals were analysed by Image Lab Software from the Molecular Imager ChemiDoc XRS Plus (Bio-Rad). The experiments were performed in triplicate (N = 3).

### Separation of head and neck

Spermatozoa were removed from the wild-type cauda epididymides by pricking the wall with a 28-G injection needle^28^ and washed in TYH medium^48^. Separation was done by a Handy Sonic model UR-20P sonicator (dial 8 for 30 seconds, TOMY SEIKO).

### Intracytoplasmic headneck or sperm injection (ICSI)

Female B6 mice were super-ovulated as reported previously^28^. At 14 hours post-hCG, oocytes were collected from the oviducts and cultured in CZB medium^49^ containing 10 mM Hepes (CZB-H) in a CO_2_ incubator. Cumulus cells were removed by a brief incubation with hyaluronidase at 0.5 mg/ml (Sigma) in CZB-H. For ICSI, separated headnecks from *Odf2* chimeric males were collected from the cauda epididymides and stored in R18S3 medium (ARK Resource) in a −80 °C freezer. Upon ICSI, the samples were thawed out, rinsed in CZB-H medium and used. For the control, wild-type spermatozoa from BDF1 males were similarly prepared. Samples were aspirated into a glass injection pipette with the help of faint piezo-electric pulses exerted with a piezo-micromanipulator (PRIME TECH) to perforate the oolemma. Then, the samples were injected into the ooplasm, and the oocytes were cultured in KSOM medium (ARK Resource) in a CO_2_ incubator until they developed into two-cell embryos. There were 4 trials for 2 wild-type males (84 eggs in total) and 7 trials for 2 chimeric males (173 eggs in total). The following day, 23 two-cell embryos from the chimeric headnecks and 45 two-cell embryos from the wild-type spermatozoa were transplanted into the oviduct of pseudopregnant ICR females. Nineteen days after the embryo transfer, foetuses were recovered by Caesarean operation from the pregnant ICR females and nursed by surrogated ICR mothers.

### IF light microscopy

Spermatozoa were not treated by any detergent, but some were briefly treated with 0.1% Triton X-100 in PBS to see the internal components. The samples were incubated with primary and secondary antibodies and Hoechst 33258 at room temperature for 1 hour. To see whether the cells were alive or dead, spermatozoa were incubated with TYH containing propidium iodide (PI) and Hoechst 33258 for 15 minute at room temperature without detergent treatment. The analyses were done by a BX50 epifluorescence microscope (Olympus) equipped with an imaging system composed of the appropriate filters for fluorescence and a CCD camera RETIGA Exi FAST 1394 (QImaging)^28^. The data were acquired using SlideBook 4 or 5 software (Intelligent Imaging Innovations). To examine spermatogenesis and whether head-tail separation occurred in the testis, testes were decapsulated, and interstitial cells were removed from the seminiferous tubules by gentle washing with TYH. After the tubules were put on microscope slides and covered by coverglasses, spermatogenic cells released from the tubules were observed by a BX50 microscope.

### Sperm motility analysis

Spermatozoa were removed from cauda epididymides, washed in TYH, and incubated for 30 minute. Sperm motility and other characteristic parameters were quantitatively analysed by an automated Sperm Motility Analysis System (SMAS; DITECT Co. Ltd.)^28^.

### Conventional electron microscopy (EM)

Spermatozoa removed from 2 chimaeras, 3 heterozygotes, and 3 wild-type males were routinely processed for transmission EM (TEM) and examined by a 1200 EX (JEOL) at 80 kV as reported previously^28^ and a JSM-6510LV (JEOL) at 15 kV for scanning EM (SEM). At least 50 spermatozoa were analysed in each experiment.

### Cryo-STEM tomography

Spermatozoa removed from 2 heterozygous males were treated with 2% Triton-X100 in PBS for 25 minute to remove the membrane and mitochondria^50^ and then replaced with HMDEK buffer (30 mM Hepes-NaOH, pH 7.2, 5 mM MgCl_2_, 1 mM DTT, 1 mM EGTA, 50 mM CH_3_COOK) supplemented with a protease inhibitor cocktail (Nacalai). As fiducial markers for tomographic reconstruction, an anti-mouse antibody conjugated with 15-nm colloidal gold was attached to spermatozoa via the anti-β-Tubulin antibody (T0198; Sigma)^51^. Bovine serum albumin coated with 15-nm gold nanoparticles (Aurion) was also applied as a fiducial marker. Spermatozoa plus colloidal gold were loaded onto homemade holey carbon grids and plunge-frozen in liquid ethane at −180 °C with a plunge-freezing device EM GP (Leica). Titan Krios (Thermo Fisher) was used for 3D structure analysis at 300 kV for the STEM mode as reported previously^52,53^ with some optimisations. The convergent angle of the STEM probe was set to 0.12 mrad (semi-angle). The image pixel size of the STEM image was 0.86 nm, the image size was 4096 × 4096, and the field of view was 3.51 μm. The dose rate of the beam was 0.1 electrons/second, and the frame rate was 30 seconds/image. The dose for one image was calculated as 3 electrons/A^2^. The tilt series was taken using a Saxton scheme step of 3 degrees up to ±70 degrees. Fifty-one images were taken for one series; the total dose was approximately 150 electrons/A^2^. The image processing for the reconstruction of 3D tomograms was performed as described previously^54^ using the IMOD software^55^.

### Movie

The sperm movement was recorded at 30 fps for 15 seconds by a DMi8 stereomicroscope equipped with neo PLAN x20 and x40 lenses and an MC170 HD camera (Leica).

### Statistics

Data are shown as the mean ± standard error of the mean (SEM). Two-tailed analyses were performed by the Mann-Whitney U test (Figs 3D and S3E), Student’s t-test (Fig. 4A,B), and Welch’s t test (Fig. 4C), using the add-in Statcel 3 software (OMS, Ltd) for Microsoft Excel. Probability (*P*) values were regarded as statistically significant; *P < 0.05 (Fig. 3D) and **P < 0.01 (Fig. 4).

## Supplementary information

SI Figs S1-S7,Table S1,Movie S1

SI Z stack images for Fig.6A

Low magnification image of Odf2Odf2Odf2Odf2+/++/++/++/+ (+/+) sperm movement.

High magnification image of Odf2Odf2Odf2Odf2+/++/++/++/+ (+/+) sperm movement.

Low magnification image of Odf2Odf2Odf2Odf2+/-+/-+/-+/- (+/-) sperm movement.

High magnification image of Odf2Odf2Odf2Odf2+/-+/-+/-+/- (+/-) sperm movement.
