# Supplementary material for: Odf2 haploinsufficiency causes a new type of decapitated and decaudated spermatozoa, Odf2-DDS, in mice
Source: Sci Rep. 2019 Oct 3;9:14249. doi: 10.1038/s41598-019-50516-2 (PMC6776547; doi:10.1038/s41598-019-50516-2)
Supplement: Supplementary file 1 — SI Figs S1-S7,Table S1,Movie S1 [file 41598_2019_50516_MOESM1_ESM.pdf]

Odf2 haploinsufficiency causes a new type of decapitated and decaudated spermatozoa, Odf2-DDS, in mice

Chizuru Ito[a,*,1], Hidenori Akutsu[b], Ryoji Yao[c], Keiichi Yoshida[a,d], Kenji Yamatoya[a,e], Tohru Mutoh[a], Tsukasa Makino[f], Kazuhiro Aoyama[g,h], Hiroaki Ishikawa[i], Koshi Kunimoto[j], Sachiko Tsukita[k], Tetsuo Noda[l], Masahide Kikkawa[f], Kiyotaka Toshimori[a,m,*,1]

[a]Department of Functional Anatomy, Reproductive Biology and Medicine, Graduate School of Medicine, Chiba University, Chiba, 260-8670, Japan
[b]Department of Reproductive Medicine, National Research Institute for Child Health and Development, Tokyo, 157-8535, Japan
[c]Department of Cell Biology, Japanese Foundation for Cancer Research (JFCR) Cancer Institute, Tokyo, 135-8550, Japan
[d]Current address: Next-generation Development Center for Cancer Treatment, Osaka International Cancer Institute, Osaka, 541-8567, Japan.
[e]Current address: Institute for Environmental & Gender-specific Medicine, Juntendo University Graduate School of Medicine, Chiba 279-0021, Japan.
[f]Department of Cell Biology and Anatomy, Graduate School of Medicine, The University of Tokyo, 7-3-1 Hongo Bunkyo-ku, Tokyo, 113-0033, Japan.
[g]Materials and Structural Analysis (ex FEI), Thermo Ficher Scientific, Shinagawa Seaside West Tower 1F, 4-12-2 HigashiSinagawa, Shinagawa-ku, Tokyo 140-0002, Japan
[h]Research Center for Ultra-High Voltage Electron Microscopy, Osaka University, 7-1 Mihogaoka, Ibaraki, Osaka 567-0047, Japan
[i]Department of Biochemistry and Biophysics, University of California San Francisco 600 16th St. San Francisco, CA 94143 USA
[j]Department of Pathology, Stanford University School of Medicine, 300 Pasteur Drive, Stanford, CA 94305, USA
[k]Graduate School of Frontier Biosciences and Medicine, Osaka University, Osaka 565-0871, Japan
[l]Director's Room, Japanese Foundation for Cancer Research (JFCR) Cancer Institute, Tokyo, 135-8550, Japan
[m]Current address: Future Medicine Research Center, Chiba University, Chiba, 260-8670, Japan

*These authors equally contributed to this work.

[1]Correspondence and requests for materials should be addressed to K.T. (email: ktoshi@faculty.chiba-u.jp) or to C.I. (email: chizuru@faculty.chiba-u.jp)

Supplemental Figures

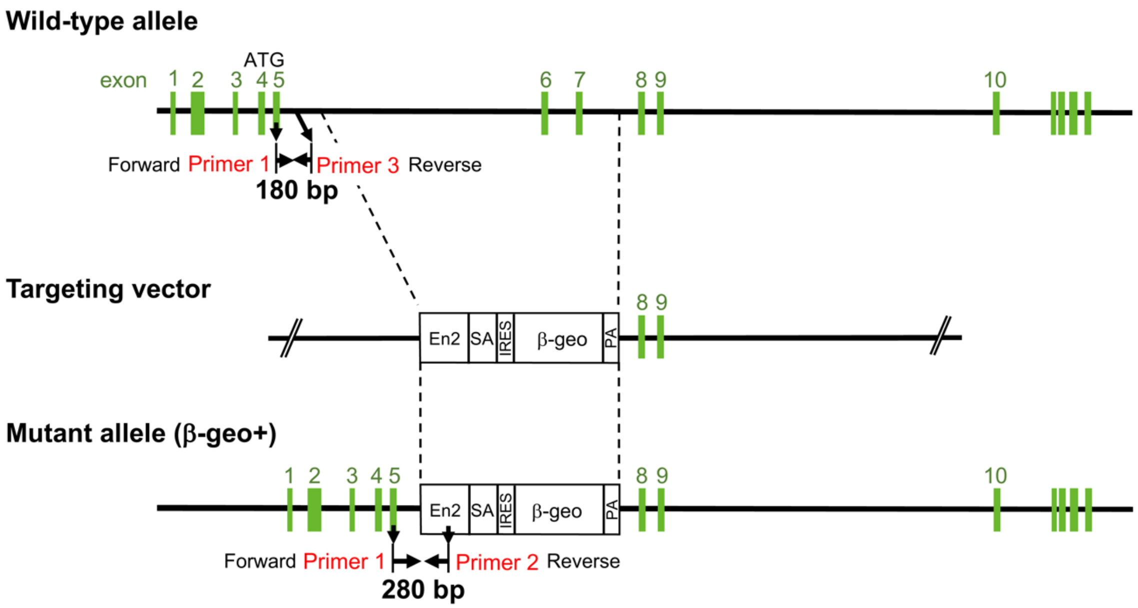

Fig. S1. Generation of *Odf2*-deficient mice. Construction of the wild-type allele, targeting vector and targeted allele (β-geo+) of the mouse Odf2 gene used in this study. This was redrawn according to the original drawing previously published by one author's group (S.T.)[21]. The targeting vector was designed to replace exons 6 and 7 with β-galactosidase/neomycin-resistance fusion gene (β-geo) combined with engrailed-2 (En2), a splicing acceptor (SA), an internal ribosome entry site (IRES) and a polyadenylation site (PA). The cassette was located on both sides of exons 6 and 7. The exon information is from the ENSEMBL accession number: ENSMUST00000046571.

Figure S1 (Ito et al)

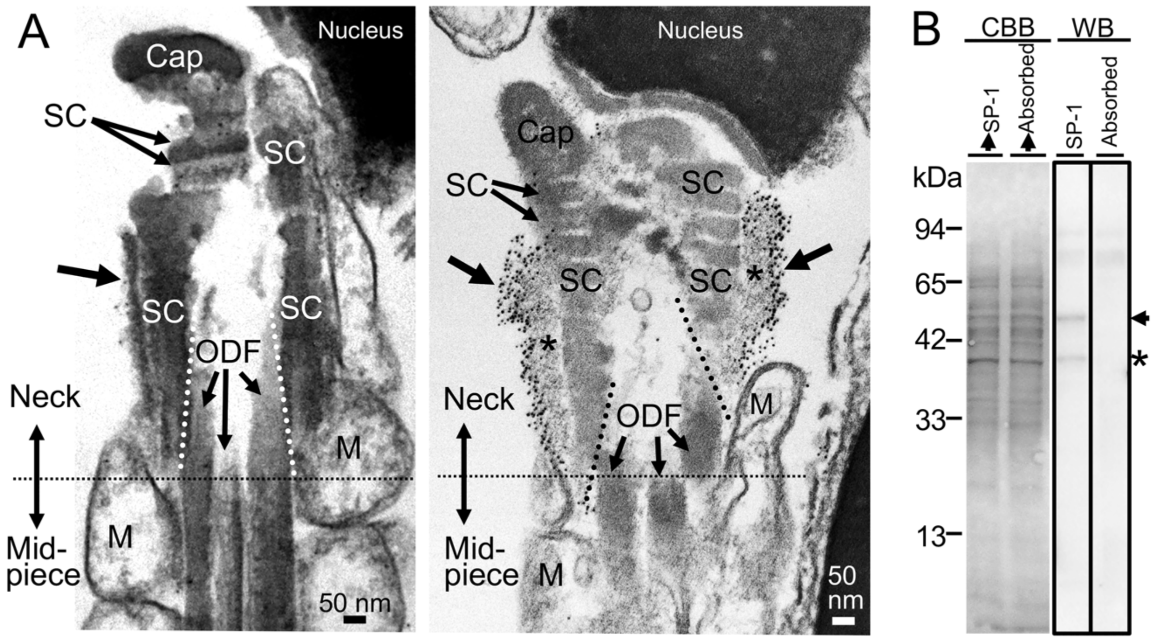

Fig. S2. Wild-type *Odf2*$^{+/+}$ normal spermatozoa. (*A*) Transmission electron micrographs. The dotted lines indicate the distal border of the segmented column and outer dense fibres (ODFs). (*Left)* A thick arrow indicates the peri-segmented column substances surrounding the segmented column (SC). (*Right)* Immunogold electron microscopy using the anti-Speriolin antibody (SP-1) conjugated with 5-nm gold particles. The thick arrows indicate many immunogold particles localised around the peri-segmented column substances (*), indicating the localisation of Speriolin. Spermatozoa were demembranated with 2% Triton-X 100 before the immunogold treatment. Cap: capitulum. M: mitochondria. SC: segmented column. (*B*) Full-length PVDF membranes for CBB staining (*Left)* and western blotting (*Right)* to validate the SP-1 antibody. The anti-Speriolin rabbit polyclonal antibody (SP-1) was raised against a synthetic 17-amino-acid peptide (RILSSIFPERVRLYGFC) conjugated to the keyhole limpet haemocyanin. The raised SP-1 was affinity purified using beads conjugated with the synthetic 17-residue peptide. The membranes from the same membrane were cropped for immunostaining with the SP-1 antibody (*Left*) and absorbed SP-1 antibody (Absorbed; negative control, *Right*). The SP-1 antibody recognises a major band at 53 kDa (arrowhead) and a minor band at 40 kDa (asterisk; *), as reported previously[25]. Negative control, absorbed SP-1 (Absorbed), does not recognise any bands. Similar positive images for Speriolin (53 kDa) with CBB staining are also shown in Fig. S5. The samples were treated with 6 M urea to extract Speriolin and prepared for western blotting as described in the Methods. The same amount (5.7 μg/lane) of sample was loaded, and the same concentrations of SP-1 and absorbed SP-1 antibodies (1.9 μg/mL) were applied. The absorbed SP-1 antibody was prepared by absorbing SP-1 with a synthetic peptide

(RILSSIFPERVRLYGFC) of a part of Speriolin that was used for raising the anti-Speriolin antibody. The experiments were performed in triplicate using different males.

Figure S2 (Ito et al)

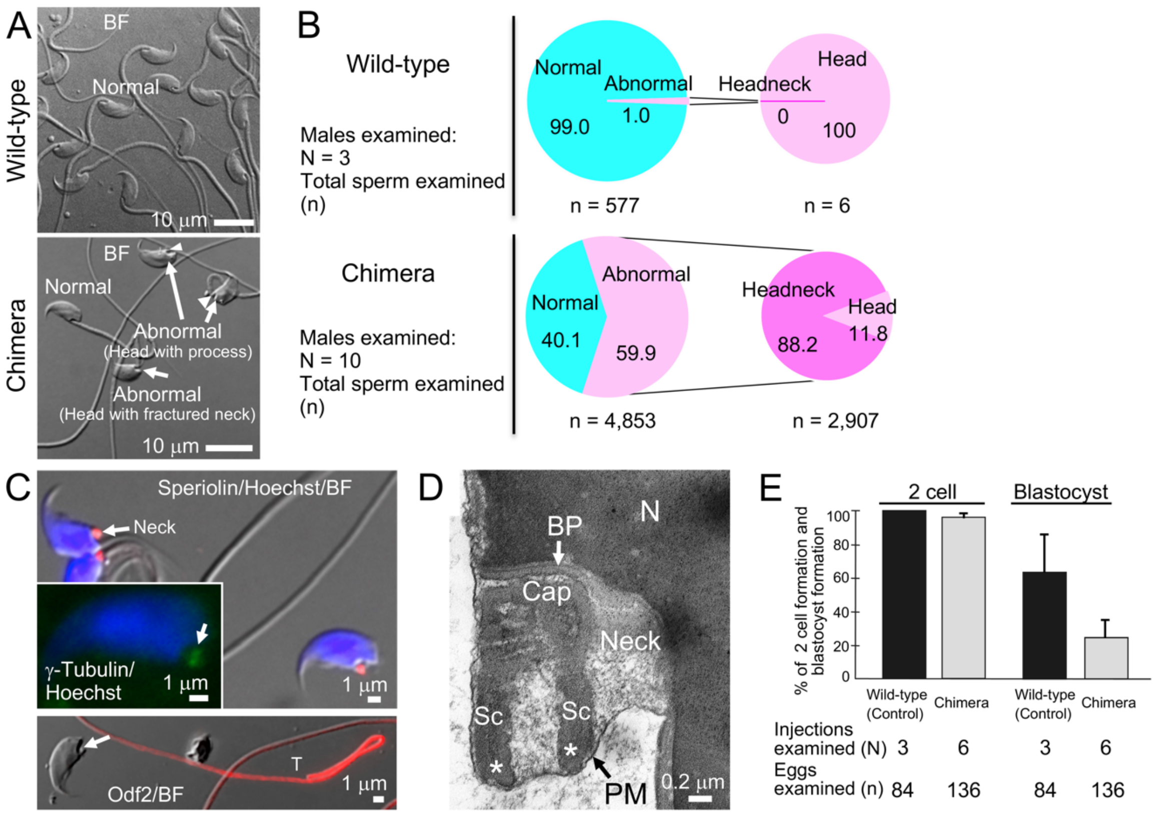

Fig. S3. Odf2 chimeric cauda spermatozoa. BF: brightfield. (*A*) Sperm morphology. (*Top*) Wild-type *Odf2*$^{+/+}$ normal spermatozoa. (*Bottom*) Chimeric abnormal spermatozoa with fractured necks and headnecks (arrowheads). Neckless heads are not observed in this image. (*B*) Percentage (%) of abnormal spermatozoa (Abnormal) and headnecks (Headnecks). (*Left)* The percentage of abnormal spermatozoa is high in the chimaera (59.9%) compared to that of the *Odf2*$^{+/+}$ (+/+) (1.0%). (*Right*) The percentage of headnecks in the abnormal spermatozoa is high in the chimaera (88.2%) compared to that in the *Odf2*$^{+/+}$ (+/+) (0%). (*C*) (*Top*) Speriolin (red) and γ-Tubulin (green, inset) in the neck of the headneck. (*Bottom*) Odf2 is detected in the separated tail (red) but is not detected in the neck of the headneck (arrow). IF microscopy with anti-Speriolin (SP-1), anti-Odf2 and anti-γ-Tubulin antibodies. Blue (Hoechst 33258): nucleus. (*D*) A headneck shown by TEM. The neck of the headneck contains neck components (basal plate: BP. Capitulum: Cap. Segmented column: Sc). The distal end of the segmented columns abruptly terminates at the region indicated by asterisks (*) but is covered by the plasma membrane (PM). Peri-segmented column substances are not well observed in this image but are recognised in the *Odf2*$^{+/-}$ spermatozoa (Fig. 6). The complex of the outer dense fibres and axial filaments are absent. N: nucleus. The total number of spermatozoa was at least 100 sperm from 5 different males for *A* and *C* (N = 5) and 20 spermatozoa from 3 different males for *D* (N = 3). (*E*) Percentage (%) of

embryonic development to the 2-cell (*Left*) and blastocyst stages (*Right*). The development into the 2-cell and blastocyst stages is not significantly different between the wild-type and chimaera. The data are presented as the mean ± SEM. Total trials of intracytoplasmic injection of headnecks or spermatozoa and total numbers of eggs examined are written at the bottom of the figures. Mann-Whitney U test (two-tailed analysis) for development to 2-cell stage. Student's *t*-test (two-tailed analysis) for development to blastocyst.

Figure S3 (Ito et al)

A

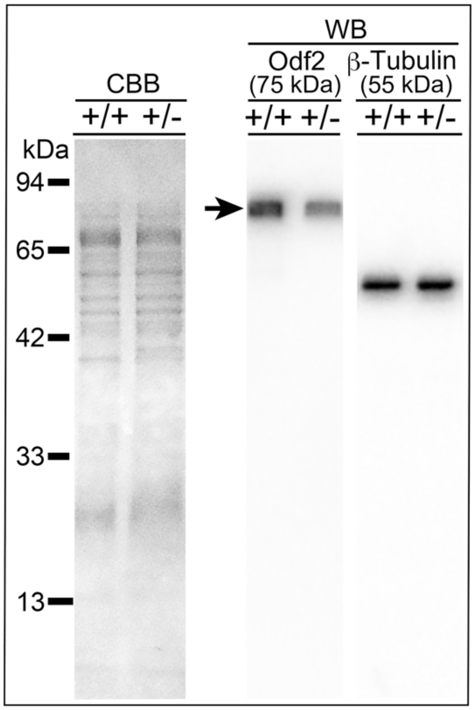

C

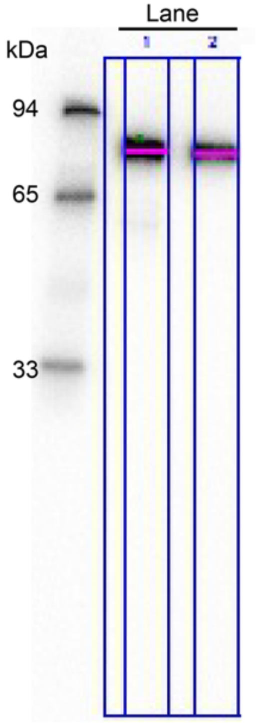

B

Odf2	Wild-type (W)	Hetero-type (HT)
Number	Lane 1	Lane 2
1	3623025	2556510
2	3523320	1819104
3	4090194	2514736
4	4761156	2647835
5		2629040
Average	3999423.75	2433445
Standard Error	282374.76	155465.1989
Ratio (HT/W): 2433445/3999423.75=0.608		

D

	Band detection:<br>Automatically detected bands with sensitivity: Low<br><br>Lane Background Subtraction:<br>Lane background subtracted with disk size: 10<br><br>Lane width: Variable
Quantity Analysis	Reference Band: Lane 1 Band 1

Lane And Band Analysis

Lane 1

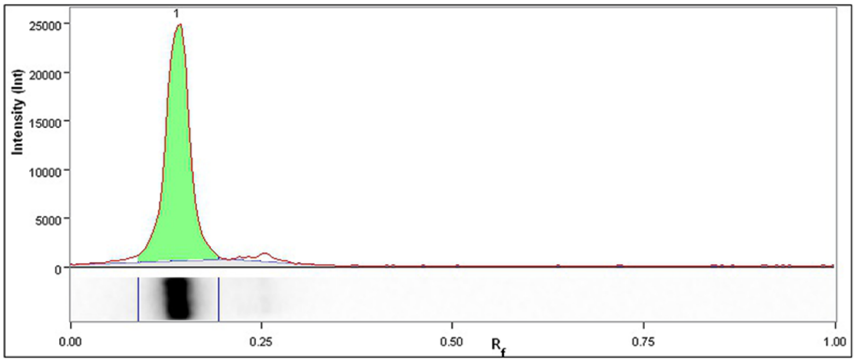

Band No.	Band Label	Mol. Wt. (KDa)	Relative Front	Volume (Int)	Abs. Quant.	Rel. Quant.	Band %	Lane %
1		N/A	0.145	3,623,025	N/A	1.00	100.0	93.0

Band Detection	Automatically detected bands with sensitivity: Low
Lane Background	Lane background subtracted with disk size: 10
Lane Width	3.07 mm

Lane 2

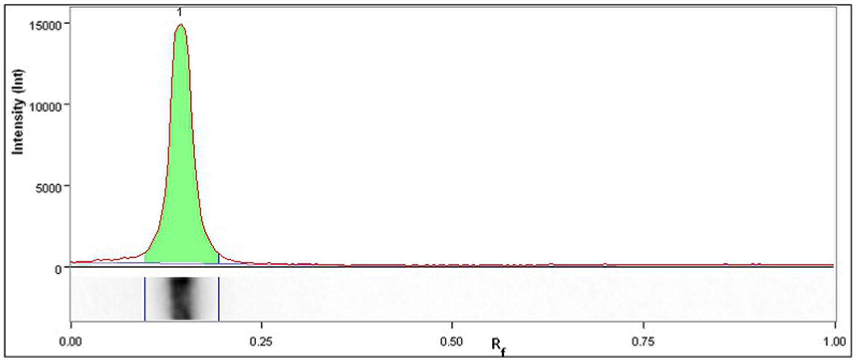

Band No.	Band Label	Mol. Wt. (KDa)	Relative Front	Volume (Int)	Abs. Quant.	Rel. Quant.	Band %	Lane %
1		N/A	0.149	2,556,510	N/A	0.71	100.0	91.4

Band Detection	Automatically detected bands with sensitivity: Low
Lane Background	Lane background subtracted with disk size: 10
Lane Width	3.07 mm

Fig. S4. (*A*) Full-length membranes for Fig. 3D. (*Left*) CBB staining. (*Middle*) Western blotting with anti-Odf2 antibody (arrow). (*Right*) Western blotting with anti-β-Tubulin antibody (internal control). Immunostaining with anti-Odf2 antibody was performed on the same membrane for CBB after destaining. The membrane for the internal control (β-Tubulin) was cropped from the different lanes from the same membrane for CBB and Odf2. (*B*) All data of band intensities in western blots. These data were obtained by Image Lab Software in Bio-Rad ChemiDoc XRS+ (molecular imager). Analysed data for Number 1 are further shown in *C* and *D* as a representative. N=4 for wild-type, 5 for hetero-type. (*C* and *D*) Band analyses for Number 1. Lane 1: wild-type. Lane 2: hetero-type. (*C*) Pink lines are automatically made on the western blot membrane for the analysis points. (*D*) Raw data obtained by the Image Lab Software in the Bio-Rad ChemiDoc XRS+ (molecular imager), in which 3,623,025 and 2,556,510 Volume (Int) are shown for Lane 1 and Lane 2, respectively.

Figure S4 (Ito et al)

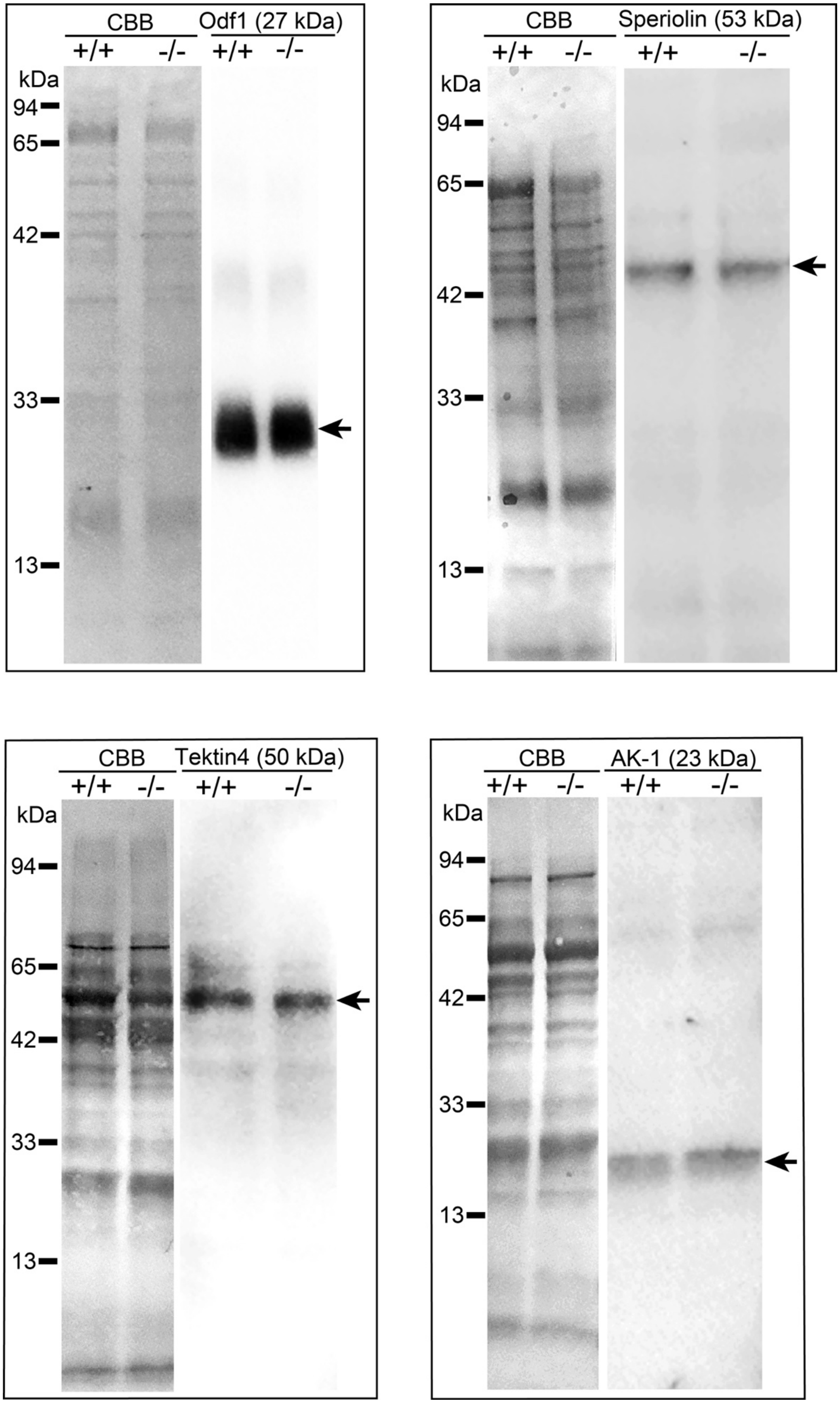
CBB
Odf1 (27 kDa)
+/+
-/-
+/+
-/-
kDa
94
65
42
33
13
CBB
Speriolin (53 kDa)
+/+
-/-
+/+
-/-
kDa
94
65
42
33
13
CBB
Tektin4 (50 kDa)
+/+
-/-
+/+
-/-
kDa
94
65
42
33
13
CBB
AK-1 (23 kDa)
+/+
-/-
+/+
-/-
kDa
94
65
42
33
13

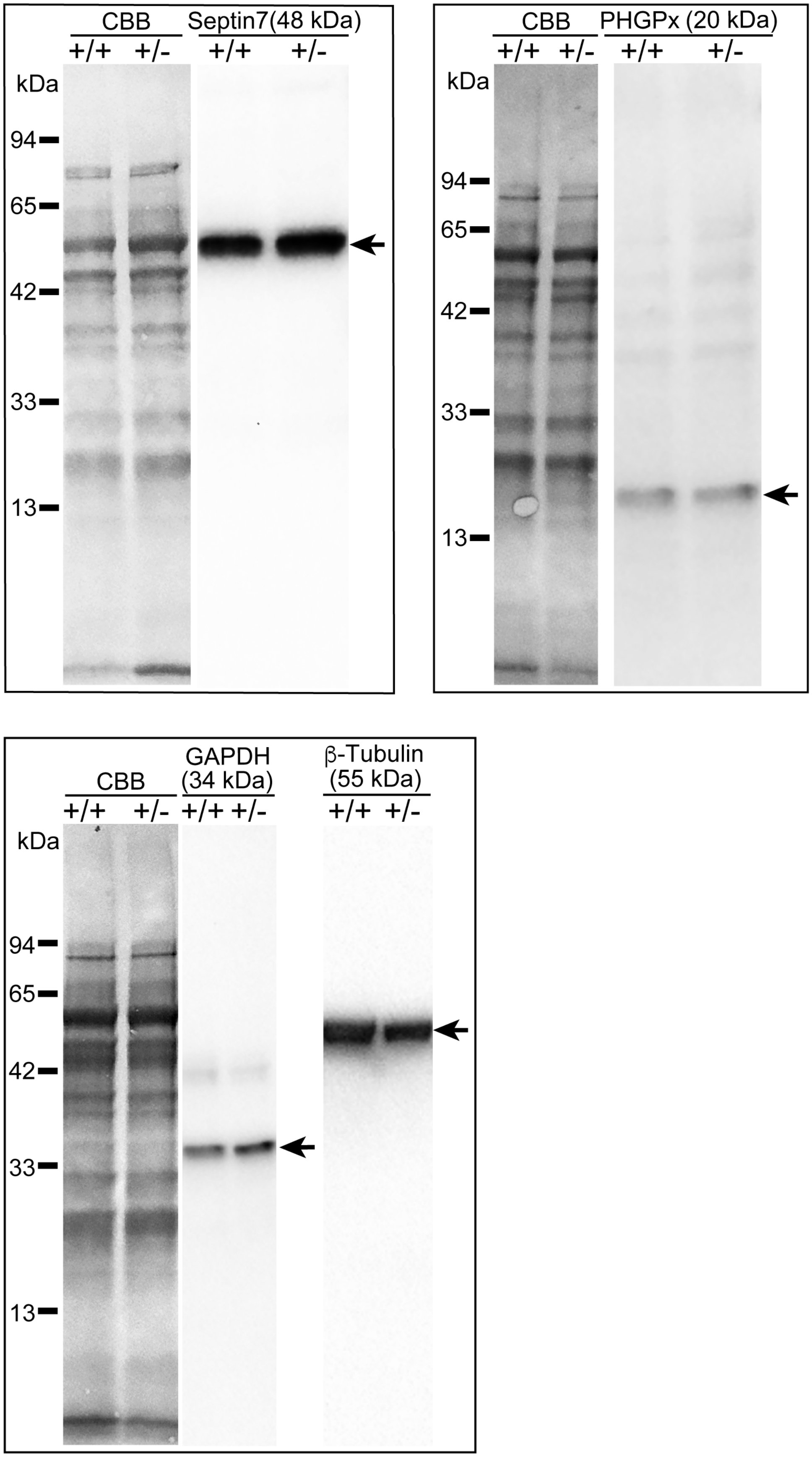
CBB
Septin7(48 kDa)
+/+ +/- +/+ +/-
kDa
94
65
42
33
13
CBB
PHGPx (20 kDa)
+/+ +/- +/+ +/-
kDa
94
65
42
33
13
CBB
GAPDH (34 kDa)
β-Tubulin (55 kDa)
+/+ +/- +/+ +/- +/+ +/-
kDa
94
65
42
33
13

Fig. S5. Full-length membranes for CBB staining (*Left*) and western blotting (*Right*) for Fig. 3E. No marked difference between the wild-type (+/+) and the heterozygous (+/-) is observed in either display (arrows). Numerals on the left side (kDa) are the positions of migrated markers. β-Tubulin; internal control. β-Tubulin is similarly used as an internal control in other blots.

Figure S5 (Ito et al)

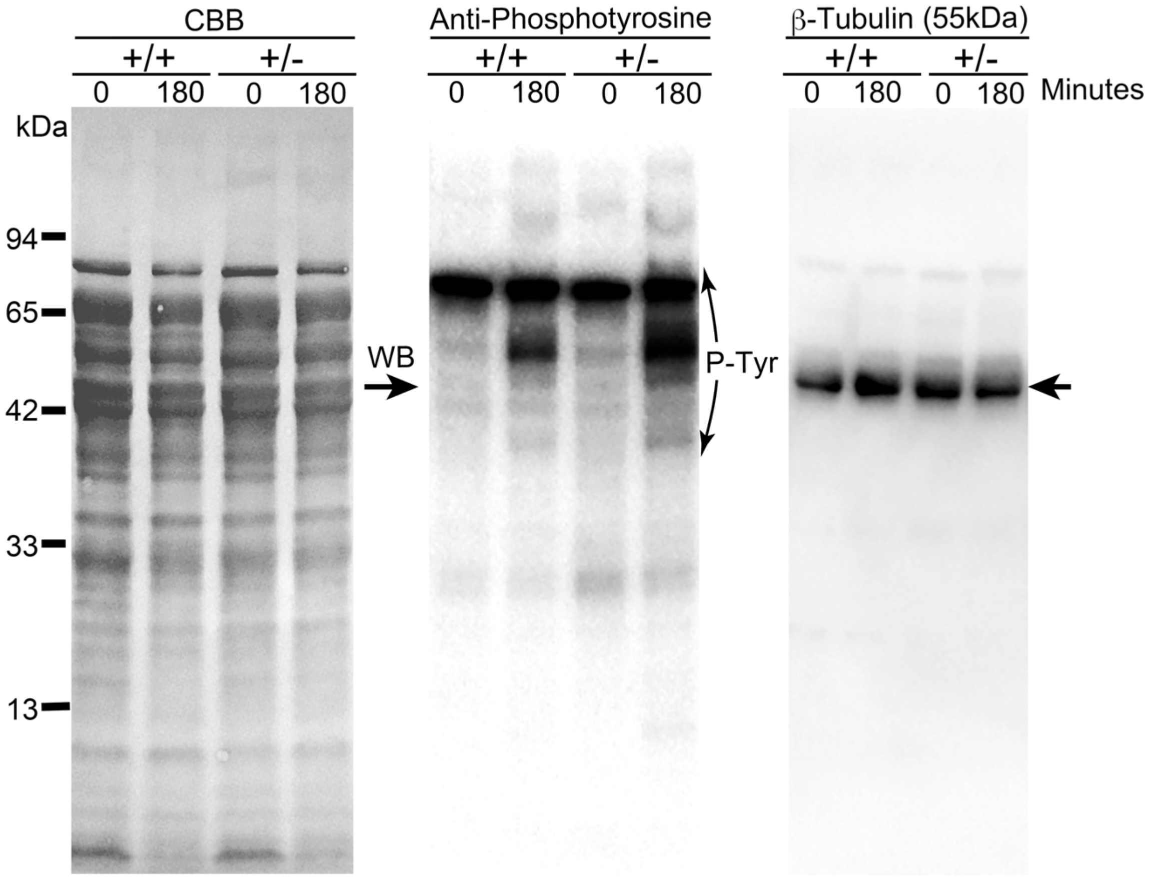

Fig. S6. Full-length membranes of CBB staining (*Left*), western blotting with anti-phosphotyrosine (P-Tyr) (*Middle*) and anti-β-Tubulin antibodies (internal control; *Right*) for Fig. 5B. CBB and western blotting with anti-phosphotyrosine antibody are cropped from the same lanes of the same membranes. The membrane for the internal control (β-Tubulin) was cropped from the same membrane as for the CBB staining and anti-phosphotyrosine blotting.

Figure S6 (Ito et al)

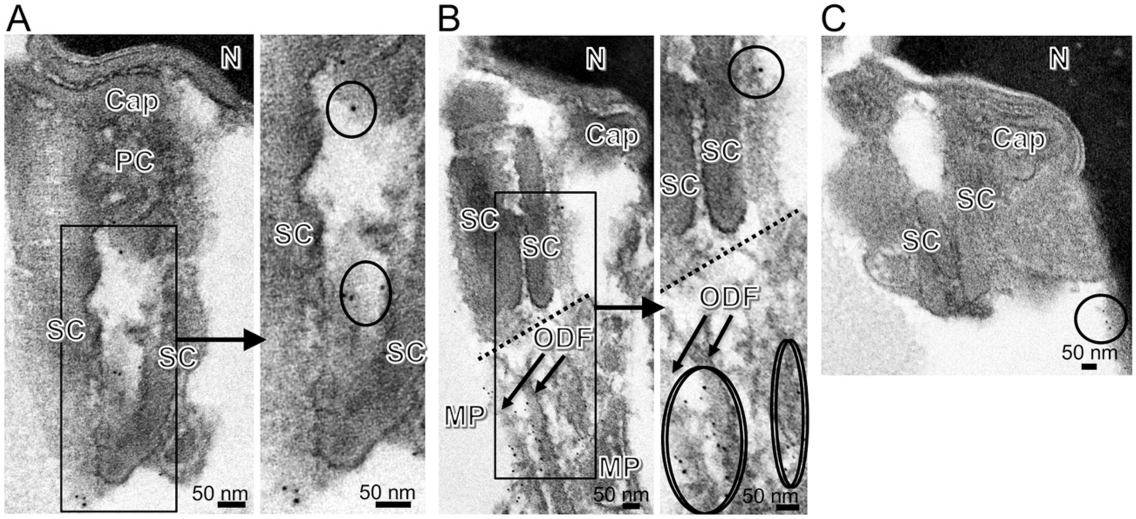

Fig. S7. Only a few immunogold particles localised in the headnecks, but relatively many did in the outer dense fibres at the midpiece in *Odf2*$^{+/-}$. Immunogold transmission electron micrographs with primary anti-Odf2 antibody and secondary antibody labelled with 5-nm gold particles. (*A* and *B*) *Odf2*$^{+/-}$ headnecks. (*A*) Completely separated headneck. (*B*) Fractured neck with the proximal part of the midpiece. This figure is similar to that shown in Fig. 3F, in which the proximal part of the midpiece is still weakly attached to the neck, as indicated by dotted lines showing the neck-midpiece connection where the distal tail is slightly flexed. *A* inset (*Right*) and *B* inset (*Right*). Because the outer dense fibres are almost completely lost in the headneck, the amount of immunogold is scarce (black circles); 2-4 in the *A* inset (*Right*) and 2 in the *B* inset (*Right*). By contrast, many immunogold particles (approximately 20 immunogold particles) are found on the outer dense fibres (ODFs) present in the middle piece (MP), which is an internal positive control (double circles in the inset in *B*). (*C*) The negative control treated with absorbed antibody SP-1 followed by secondary antibody labelled with 5-nm gold particles shows only background-level or nonspecific labelling of immunogold particles at the base of the head (circle). At least 50 sperm from 2 different males were examined in each experiment. N: nucleus. Cap: capitulum. ODF: outer dense fibre. SC: segmented column.

Figure S7 (Ito et al)

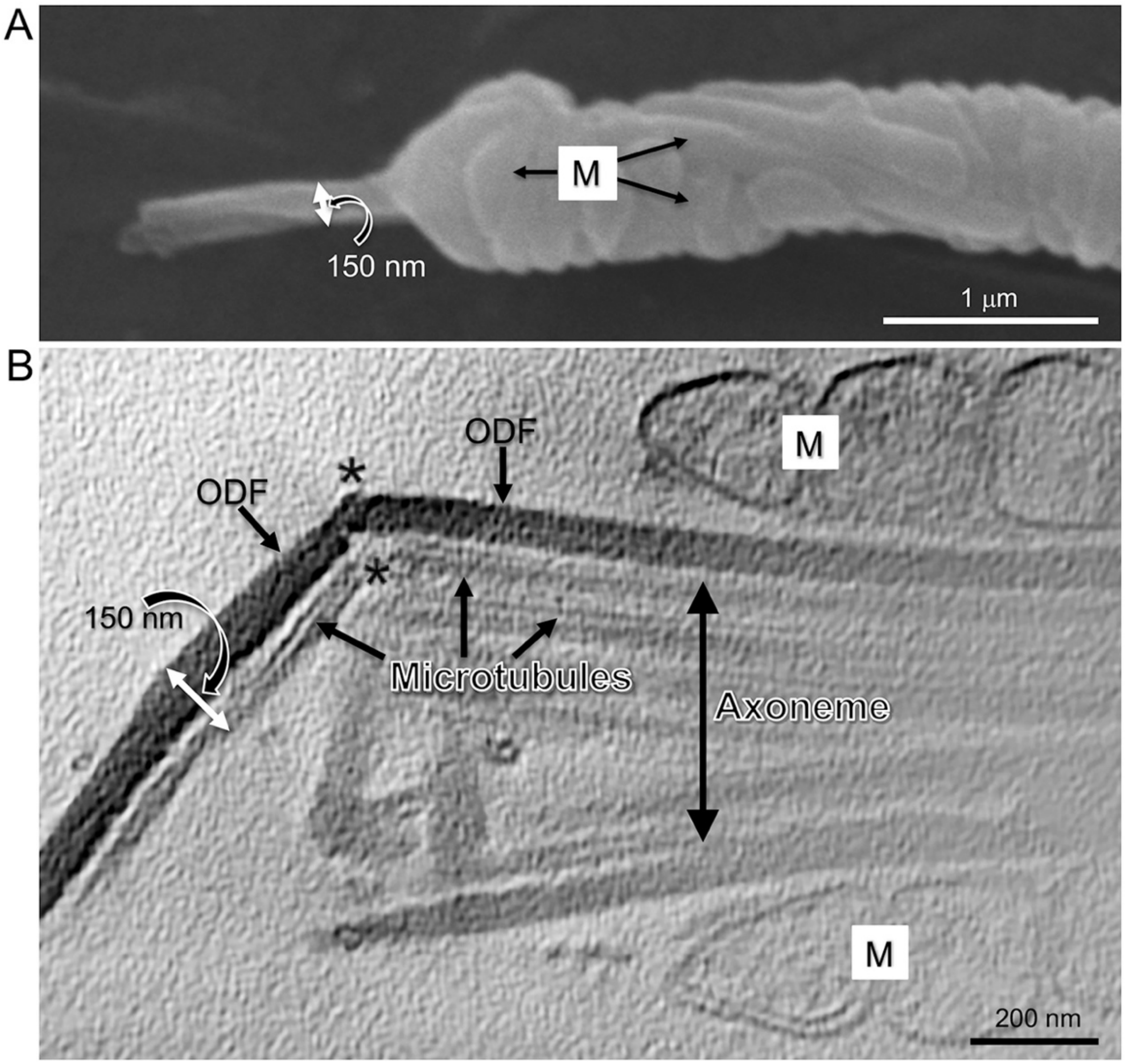

Fig. S8. Higher magnification of a rod-shaped structure projected from the separated tail (related to Figs. 6D and 6E). (*A*) The rod-shaped structure is approximately 150 nm in diameter. (*B*) A rod-shaped structure is shown by STEM tomography. A different angle of Figure 6E of the three-dimensional images is shown here, where a rod-shaped structure of approximately 150 nm in diameter consists of the complex of the outer dense fibres (ODFs) and microtubules. The asterisks (*) indicate the top of the separated tail where both the outer dense fibres and microtubules are fractured. M: mitochondria.

Figure S8 (Ito et al)

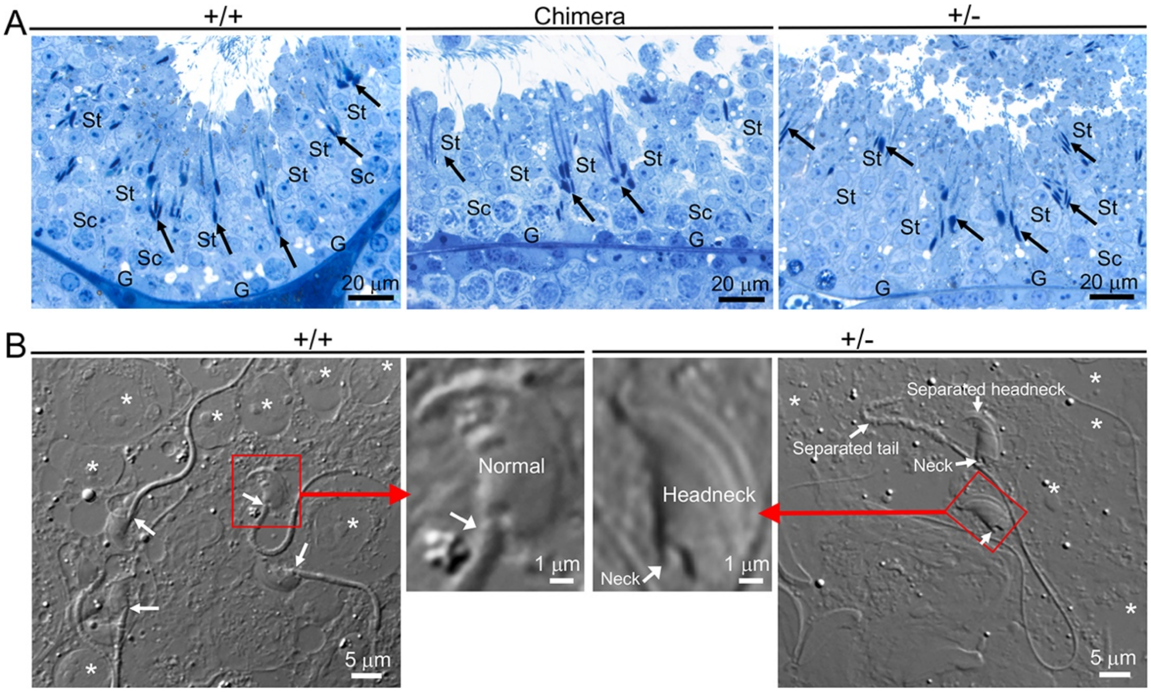

Fig. S9. Normal spermatogenesis and neck-midpiece separation in *Odf2*$^{+/-}$ testes. Light micrographs. (*A*) Spermatogenesis (stage V) shown by toluidine blue staining. Spermatogenesis proceeds normally in *Odf2*$^{+/-}$ (+/-) (*Right*) as well as in *Odf2*$^{+/+}$ (+/+) (*Left*) and Odf2 chimaera males (*Middle*). Neck-midpiece separation is not detected in conventional thin sections of both *Odf2*$^{+/-}$ and chimaera testes. G: spermatogonia. Sc: spermatocytes. St: spermatids. N = 3 (different males). (*B*) DIC images of spermatogenic cells freed from seminiferous tubules. (*Left*) Normal head-neck connections (arrows) found in *Odf2*$^{+/+}$ (+/+). (*Right*) Abnormal neck-midpiece separations (arrows) found in *Odf2*$^{+/-}$ (+/-) headnecks. Insets (*Centre*): Higher magnification of the rectangular areas to show the neck regions, especially for *Odf2*$^{+/-}$ (+/-) neck-midpiece separation (arrow) or the occurrence of headnecks, compared to the normal *Odf2*$^{+/+}$ (+/+) neck-midpiece connection (arrow). The seminiferous tubules were minced with scissors. Asterisks (*): round spermatids. N = 6 (different males).

Figure S9 (Ito et al)

Antibodies (characteristic) or chemicals	Host*	Dilution (concentration) for western blotting	Dilution or concentration for IIF, cryo-STEM or chemical staining	Reeference or source
Anti-AK1 (E-8)	M	1/500 of the stock solution (0.2 mg/ml)		Santa Cruz Biotechnology, Inc. (Dallas, TX)
Anti-β-tubulin	M	1/10,000 of the stock solution (0.5 mg/ml)		FUJIFILM Wako Pure Chemicals (Osaka, Japan)
Anti-β-tubulin	M		1/200 of the stock solution (0.5 mg/ml)	Sigma-Aldrich (St Louis, MO)
Anti-β-tubulin (T0198)	M		1/5,000; fiducial marker for cryo-STEM	Sigma-Aldrich (St Louis, MO)
Antii-equatorin (MN9)	M		1/20,000 of the stock solution (4 mg/ml); marker for acrosome reaction	Ito et al (2018)[28]
Anti-γ-tubulin (clone GTU-88)	M		1/200 of the stock solution (28.3 mg/ml); marker for sperm neck	Sigma-Aldrich (St Louis, MO)
Anti-human Septin7	R	1/2,000 of the stock solution (0.05 mg/ml))		Immuno-Biological Laboratories (Gunma, Japan)
Anti-Odf1 (G-11)	G	1/1,000 of the stock solution (0.2 mg/ml)		Santa Cruz Biotechnology, Inc. (Dallas, TX)
Anti-Odf2 (N-20)	G	1/2,000 of the stock solution (0.2 mg/ml)	1/100 of the stock solution (0.2 mg/ml)	Santa Cruz Biotechnology, Inc. (Dallas, TX)
Anti-Odf2	R	1/5,000 of the stock solution (0.53 mg/ml)	1/200 of the stock solution (0.53 mg/ml)	Proteintech Group (Rosemont, Ill)
Anti-Odf2	R		1/500 of the stock solution of the original gift	Kyoto University (Kyoto, Japan)
Anti-phosphotyrosine (clone 4G10)	M	1/1,000 of the stock solution (4 mg/ml)		Merck Millipore (Damstadt, Germany)
Anti-Speriolin (SP-1)	R		1/250 of the stock solution (1.4 mg/ml)	This study
Anti-Tektin4	R	1/5,000 of the stock solution of the original gift		IIda et al (2005)[24]
Anti-goat IgG (Alexa Fluor 488)	D		1/2,000 of the stock solution (2 mg/ml) for Odf2	Molecular Probe (Eugene, OR)
Anti-mouse IgG (Alexa Fluor 488)	G		1/2,000 of the stock solution (2 mg/ml) for β-tubulin and γ-tubulin	Molecular Probe (Eugene, OR)
Anti-mouse IgG (15 nm gold conjugated)	G		1/50; fiducial marker for cryo-STEM	BBI solution (South Wales, UK)
Anti-rabbit IgG (Alexa Fluor 546)	G		1/2,000 of the stock solution (2 mg/ml) for Odf2 and Speriolin	Molecular Probe (Eugene, OR)
Anti-goat IgG (Horseradish peroxidase)	D	1/10,000 of the stock solution (0.4 mg/ml)		Jackson Immunoresearch (West Grove, PA)
Anti-mouse IgG (Horseradish peroxidase)	G	1/10,000 of the stock solution (0.4 mg/ml)		Jackson Immunoresearch (West Grove, PA)
Anti-rabbit IgG (Horseradish peroxidase)	G	1/10,000 of the stock solution (1mg/ml)		Sera Care Life Sciences (Milford, MA)
Hoechst 33342			1.7μg/ml for nuclear staining	Thermo Fisher Scientific (Waltham, MA)
Propidium iodide			1μM/ml for nuclear staining	Thermo Fisher Scientific (Waltham, MA)
Hoechst 33258			1.7μg/ml for nuclear staining	Thermo Fisher Scientific (Waltham, MA)
MitoTracker Red CMXRos®			0.5μM/ml for mitochondria staining	Thermo Fisher Scientific (Waltham, MA)
Pregnant mare serum gonadotropin (PMSG)			5 IU for superovulation	ASKA Pharmaceutical Co., Ltd (Tokyo, Japan)
human Chorionic Gonadotropin (hCG)			5 IU for superovulation	ASKA Pharmaceutical Co., Ltd (Tokyo, Japan)
Bovine serum albumin (15 nm gold conjugated)			1/50; fiducial marker for cryo-STEM	Aurion (Wageningen, Netherlands)

*D,Donkey; G, Goat; M, Mouse; R, Rabbit.

Table S1. Antibodies and chemicals used in this study.

Table S1 (Ito et al)

Movie S1. Failure of *Odf2*$^{+/-}$ sperm movement. (*A*) and (*C*) Low-magnification images with the x20 objective lens. (*B*) and (*D*) Magnified images with the x40 objective lens. (A) and (B) *Odf2*$^{+/+}$ (+/+) control. Spermatozoa move vigorously (*A*), and many can readily reach the edge of the drop of culture medium (*B*). (*C*) and (*D*) *Odf2*$^{+/-}$ (+/-). The tailless headnecks are immotile (*C*), but the tails are motile (*D*). Since the tail movement is lethargic with no significant forward motility, the tails are unable to reach the edge of the culture medium drop, except by accidental collision (*D*). These movies are in accord with the sperm movement analyses by SMAS shown in Fig. 4.

Movie S1 (Ito et al)
