# Supplementary material for: Odf2 haploinsufficiency causes a new type of decapitated and decaudated spermatozoa, Odf2-DDS, in mice
Source: Sci Rep. 2019 Oct 3;9:14249. doi: 10.1038/s41598-019-50516-2 (PMC6776547; doi:10.1038/s41598-019-50516-2)

***Odf2* haploinsufficiency causes a new type of decapitated and decaudated spermatozoa, Odf2-DDS, in mice**

Chizuru Ito[a,*,1], Hidenori Akutsu[b], Ryoji Yao[c], Keiichi Yoshida[a,d], Kenji Yamatoya[a,e], Tohru Mutoh[a], Tsukasa Makino[f], Kazuhiro Aoyama[g,h], Hiroaki Ishikawa[i], Koshi Kunimoto[j], Sachiko Tsukita[k], Tetsuo Noda[l], Masahide Kikkawa[f], Kiyotaka Toshimori[a,m,*,1]

[a]Department of Functional Anatomy, Reproductive Biology and Medicine, Graduate School of Medicine, Chiba University, Chiba, 260-8670, Japan
[b]Department of Reproductive Medicine, National Research Institute for Child Health and Development, Tokyo, 157-8535, Japan
[c]Department of Cell Biology, Japanese Foundation for Cancer Research (JFCR) Cancer Institute, Tokyo, 135-8550, Japan
[d]Current address: Next-generation Development Center for Cancer Treatment, Osaka International Cancer Institute, Osaka, 541-8567, Japan.
[e]Current address: Institute for Environmental & Gender-specific Medicine, Juntendo University Graduate School of Medicine, Chiba 279-0021, Japan.
[f]Department of Cell Biology and Anatomy, Graduate School of Medicine, The University of Tokyo, 7-3-1 Hongo Bunkyo-ku, Tokyo, 113-0033, Japan.
[g]Materials and Structural Analysis (ex FEI), Thermo Ficher Scientific, Shinagawa Seaside West Tower 1F, 4-12-2 HigashiSinagawa, Shinagawa-ku, Tokyo 140-0002, Japan
[h]Research Center for Ultra-High Voltage Electron Microscopy, Osaka University, 7-1 Mihogaoka, Ibaraki, Osaka 567-0047, Japan
[i]Department of Biochemistry and Biophysics, University of California San Francisco 600 16th St. San Francisco, CA 94143 USA
[j]Department of Pathology, Stanford University School of Medicine, 300 Pasteur Drive, Stanford, CA 94305, USA
[k]Graduate School of Frontier Biosciences and Medicine, Osaka University, Osaka 565-0871, Japan
[l]Director's Room, Japanese Foundation for Cancer Research (JFCR) Cancer Institute, Tokyo, 135-8550, Japan
[m]Current address: Future Medicine Research Center, Chiba University, Chiba, 260-8670, Japan

*These authors equally contributed to this work.

[1]Correspondence and requests for materials should be addressed to K.T. (email: ktoshi@faculty.chiba-u.jp) or to C.I. (email: chizuru@faculty.chiba-u.jp)

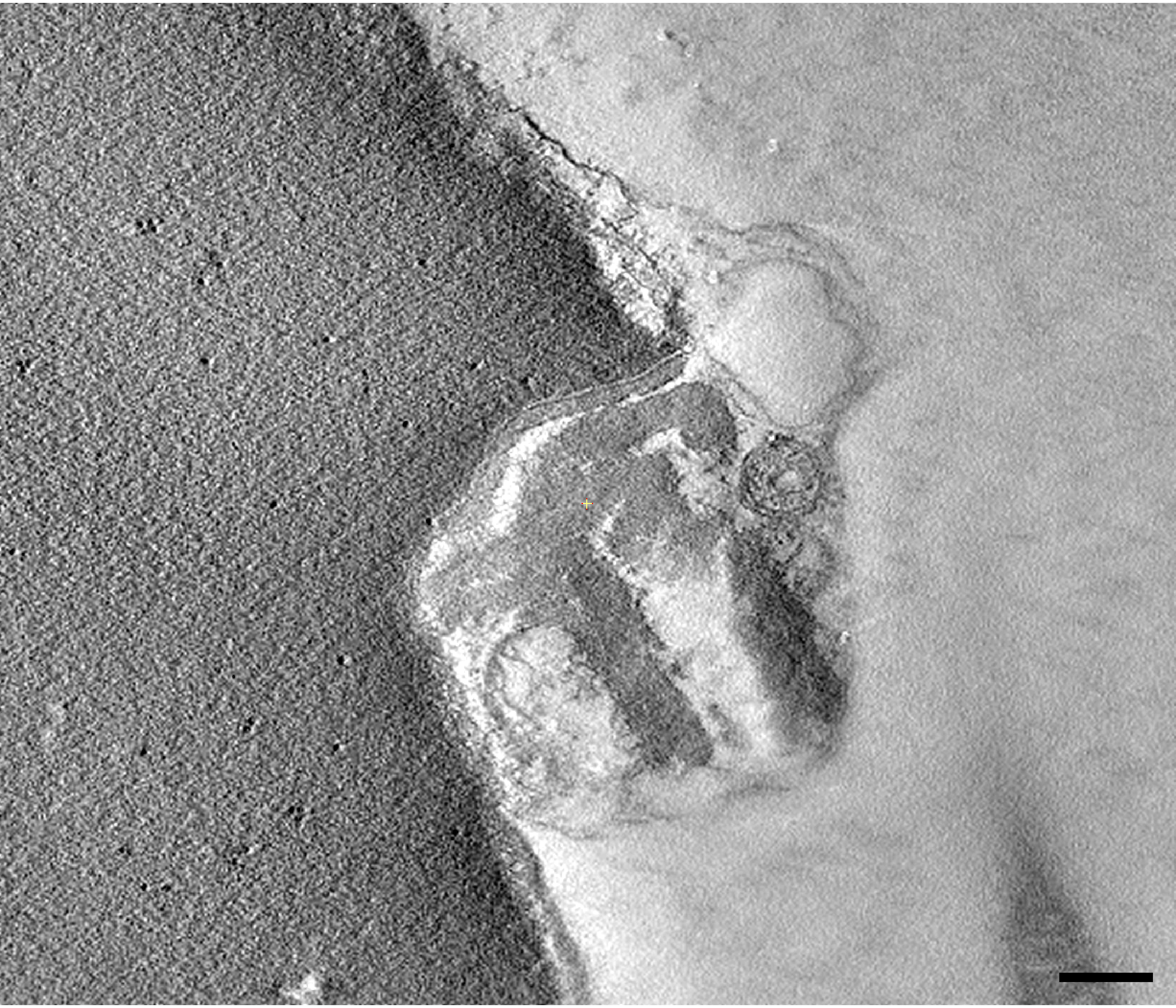

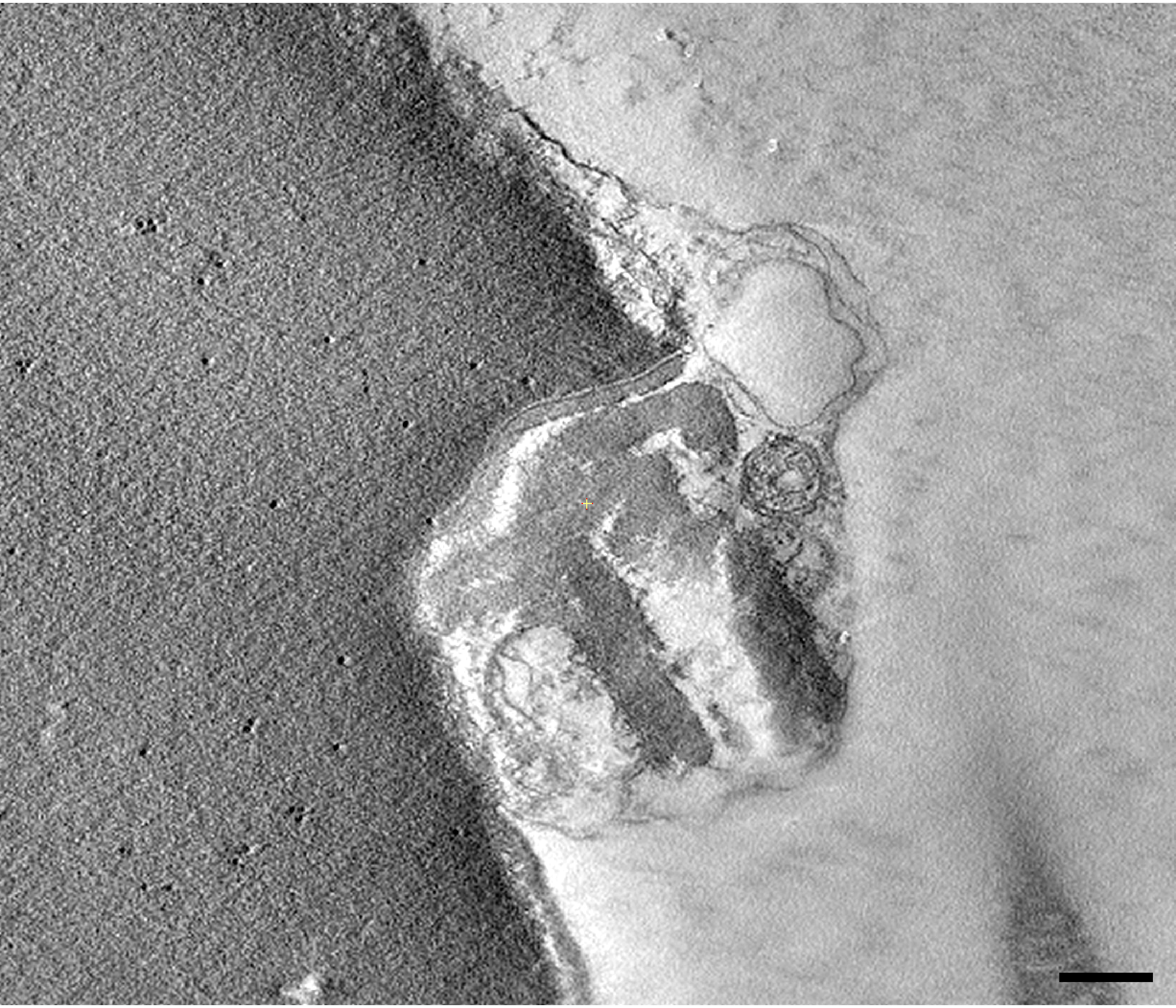

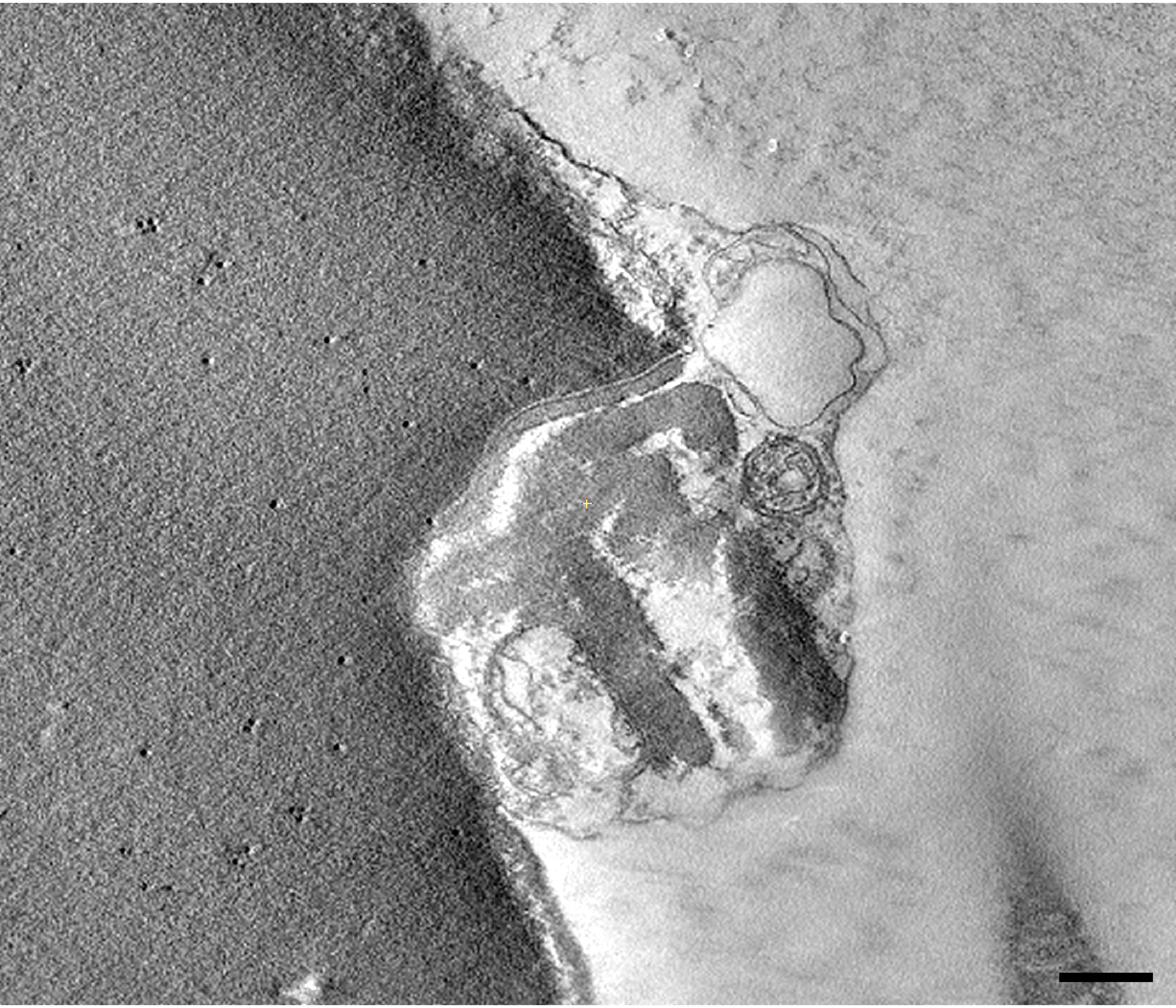

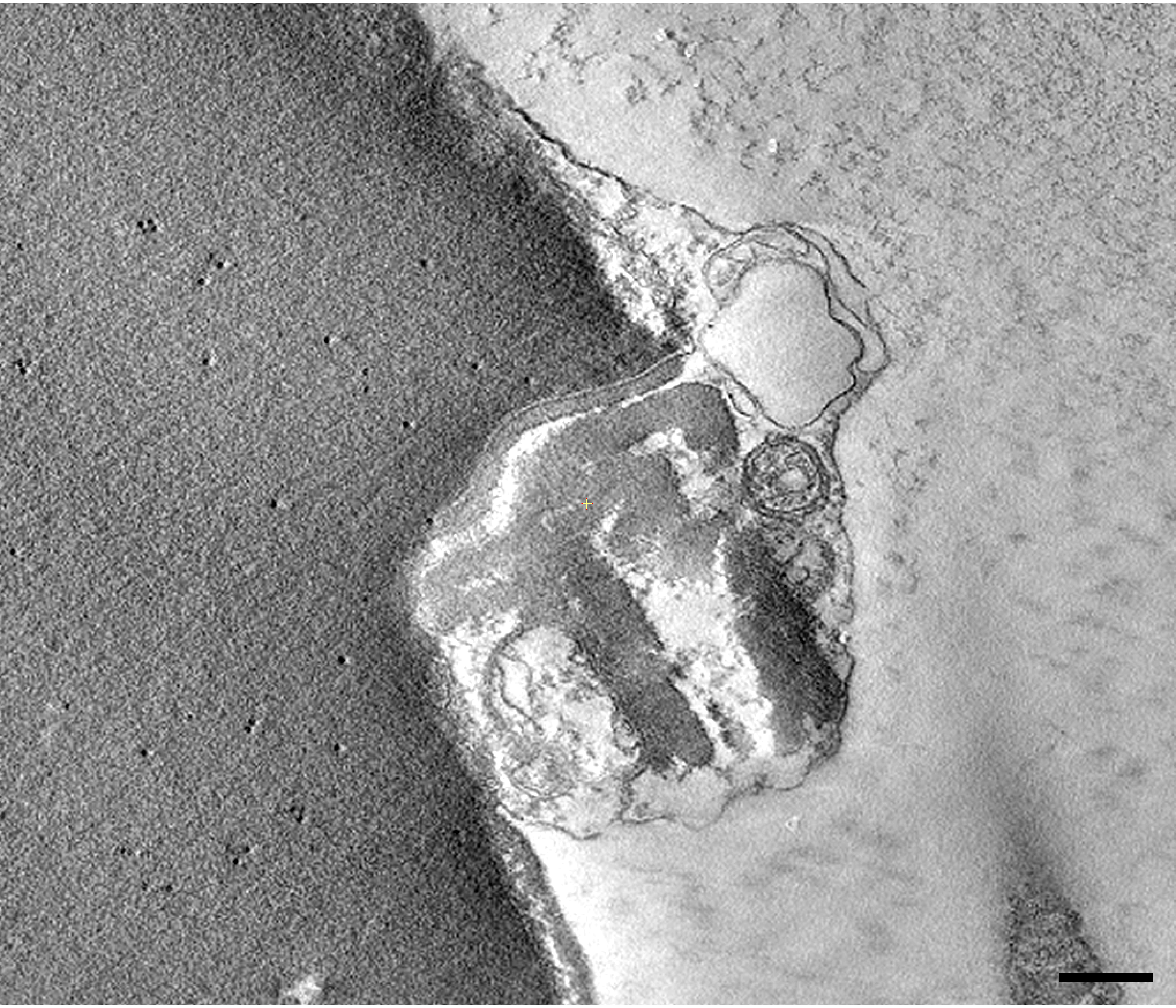

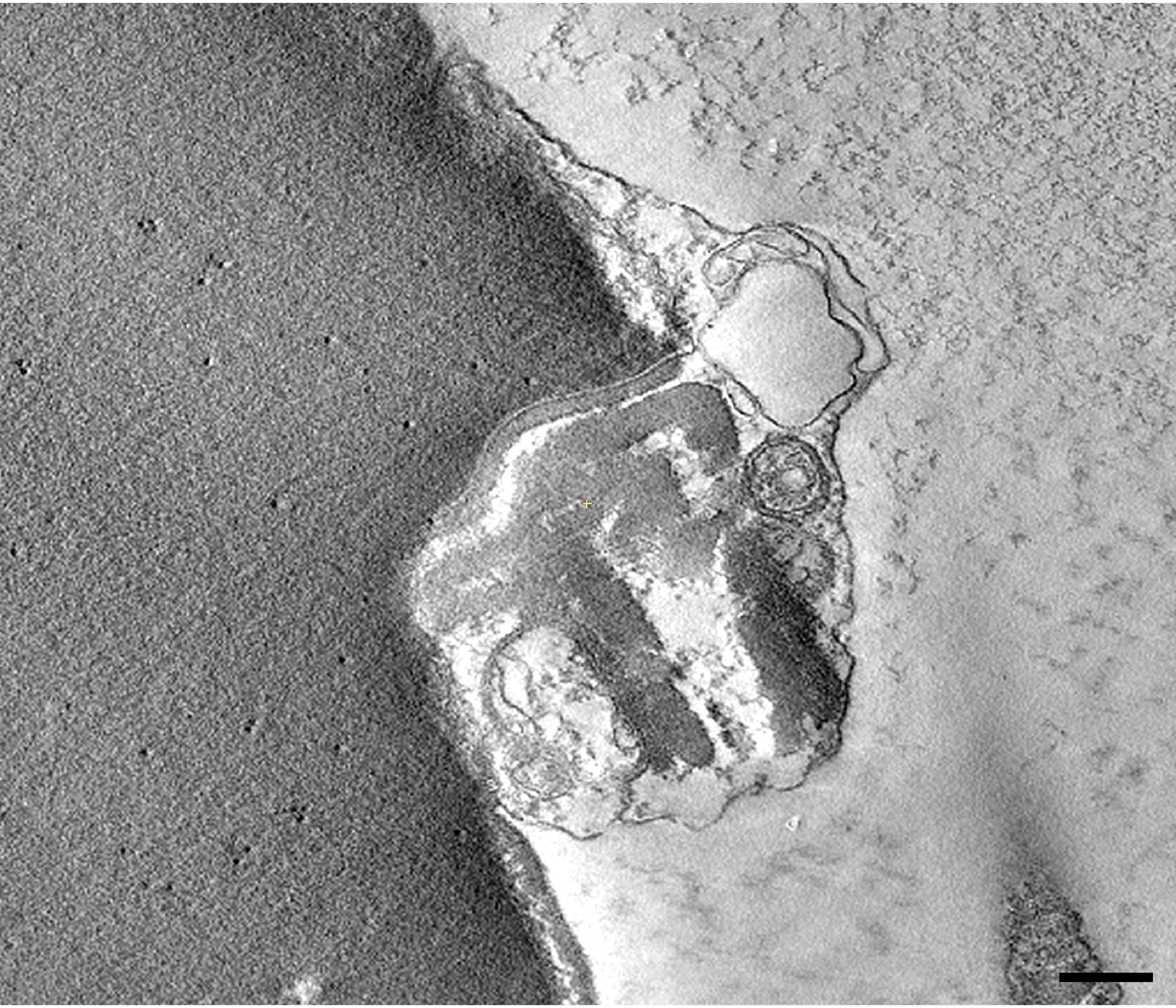

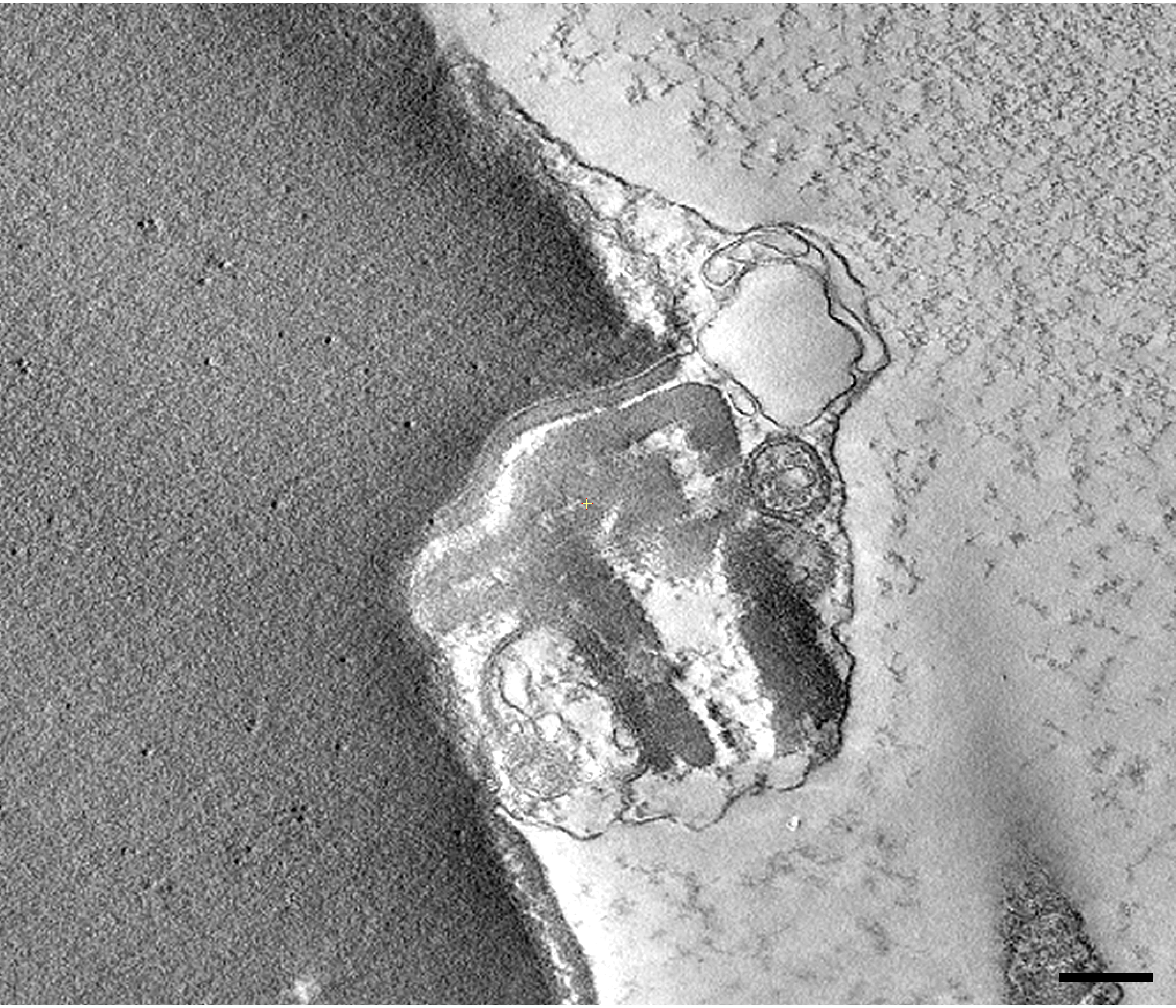

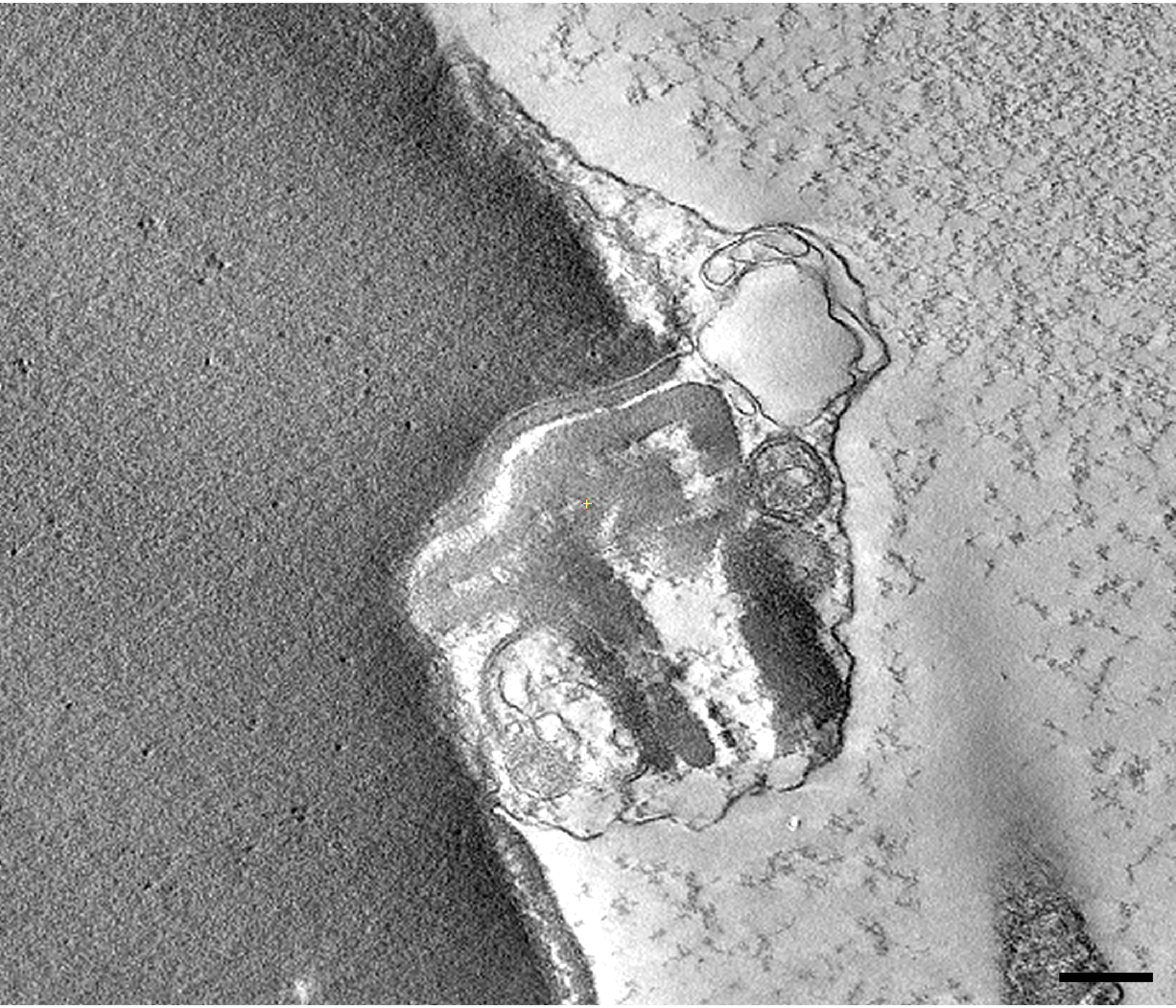

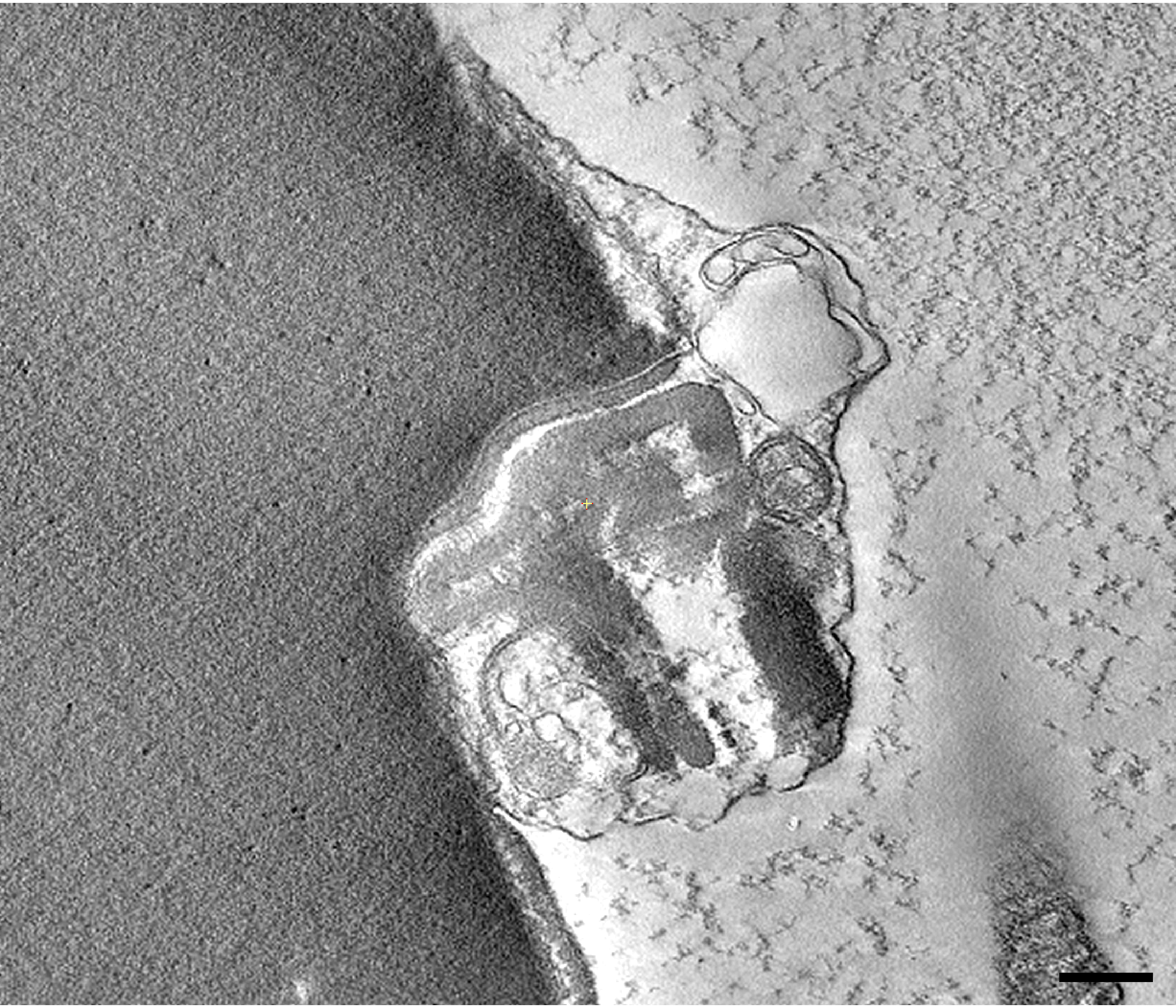

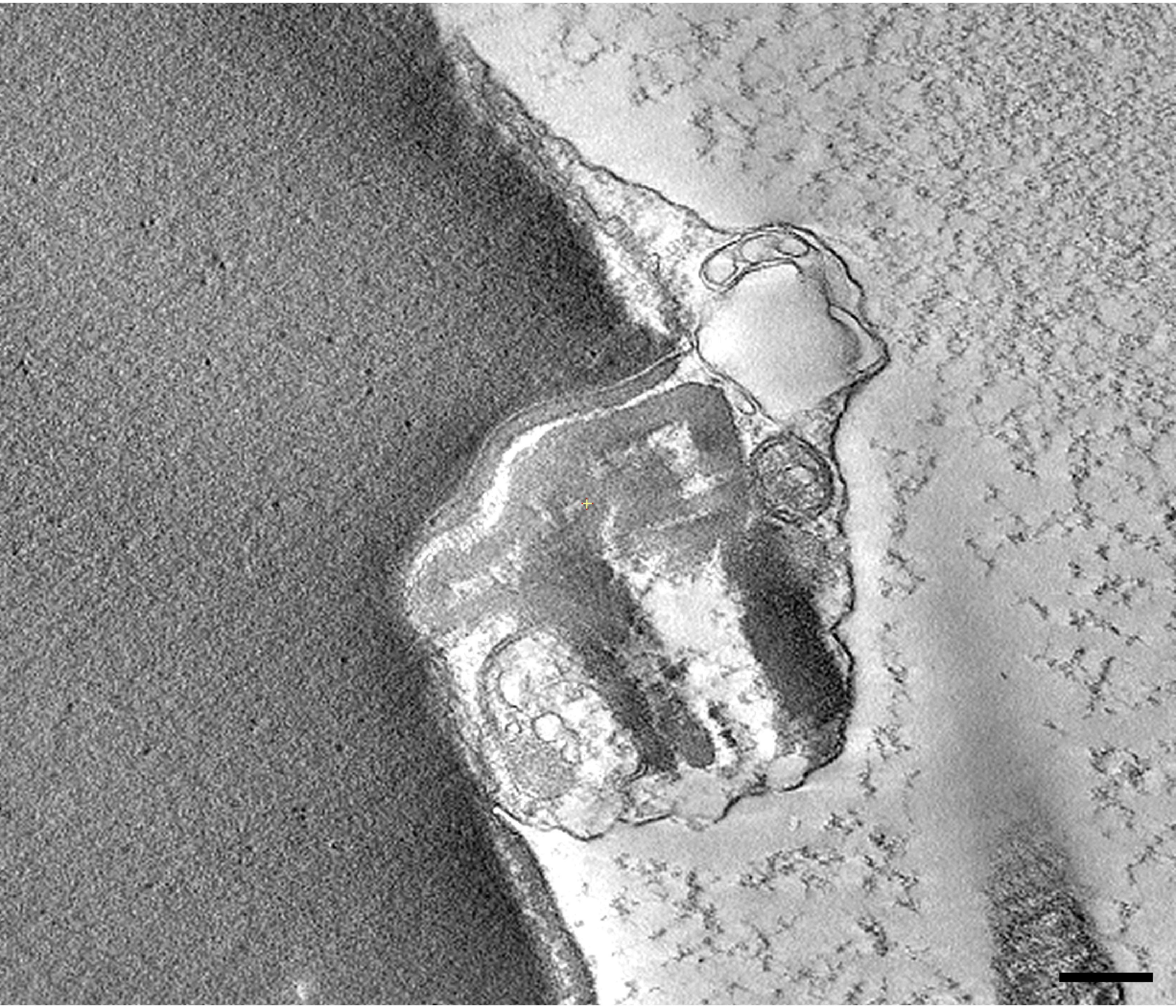

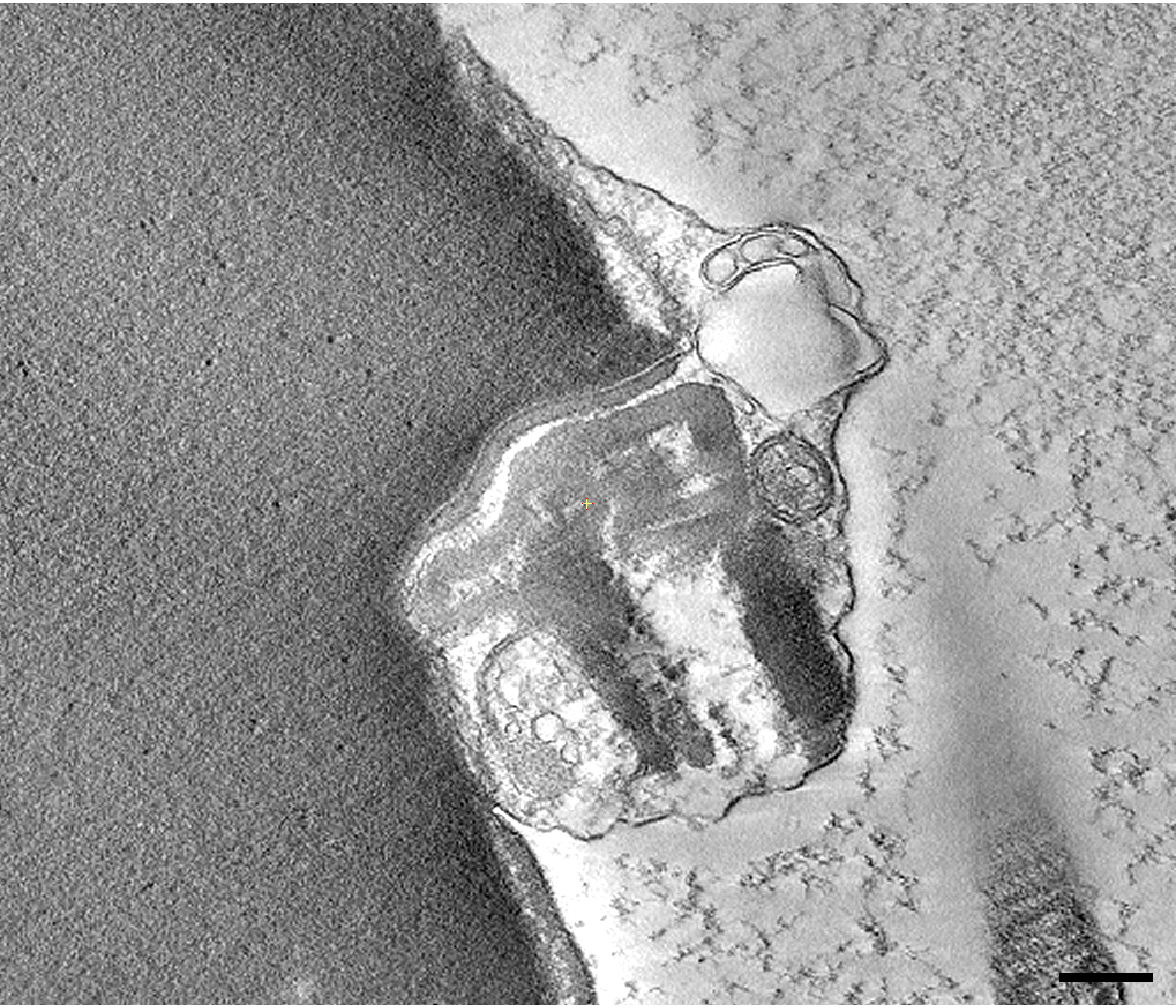

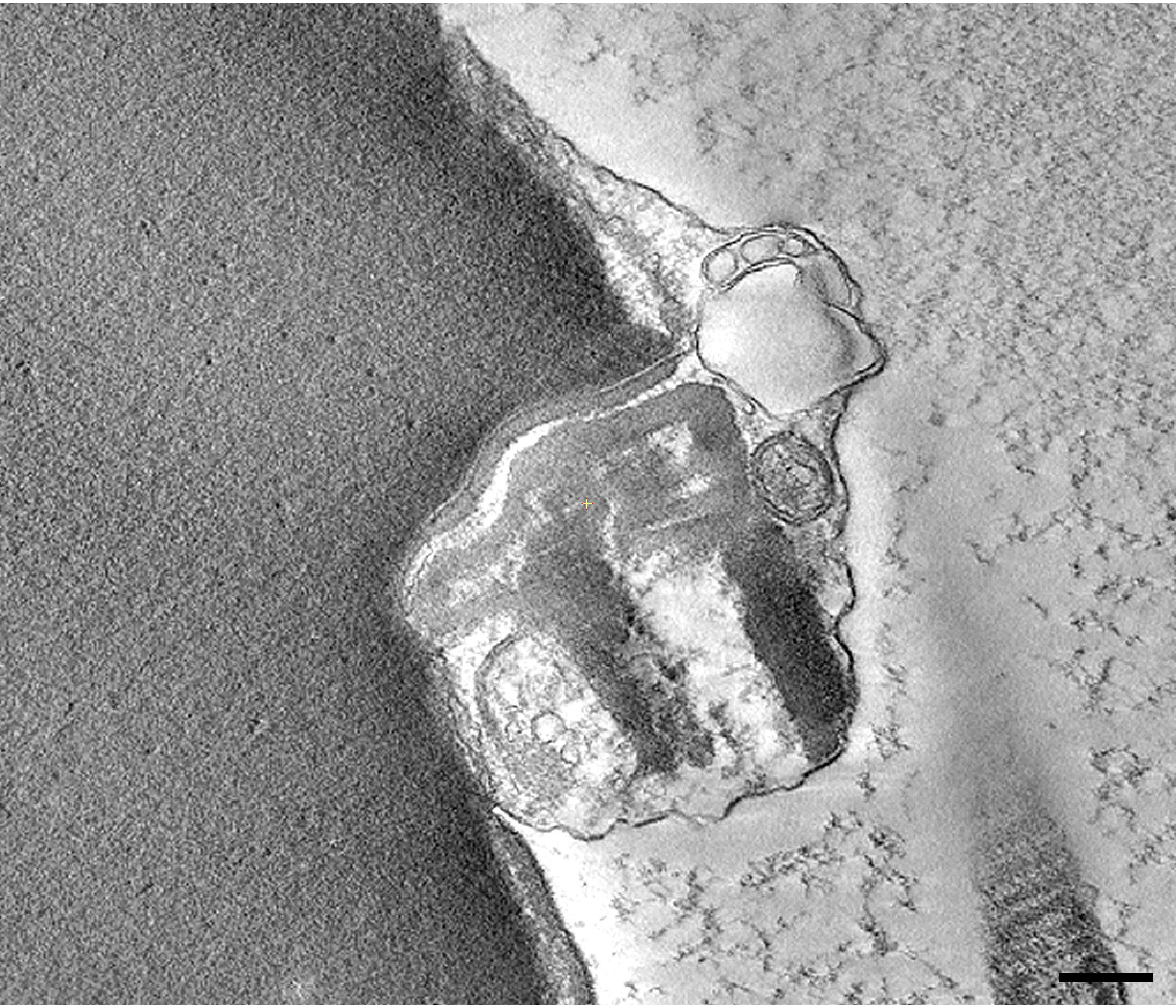

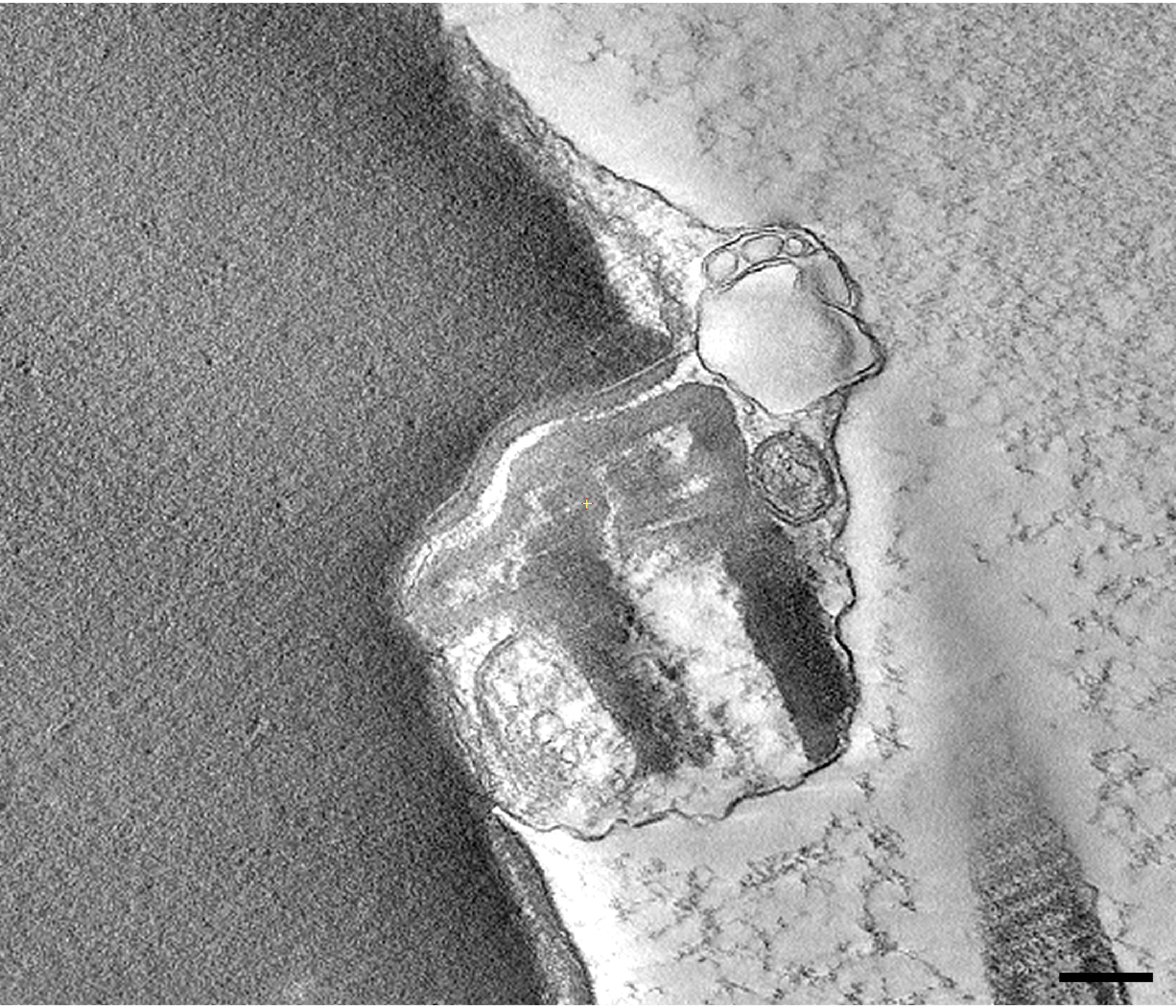

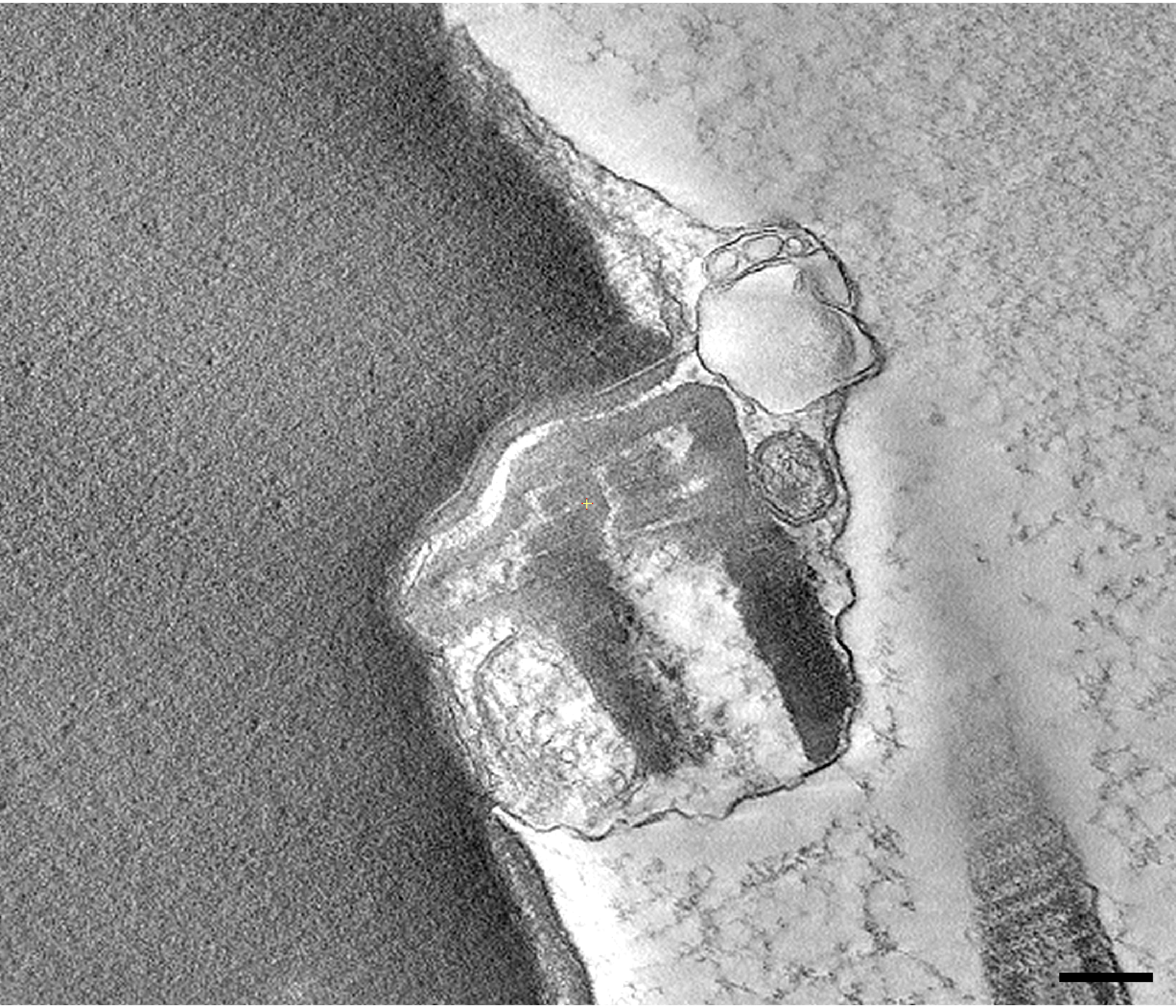

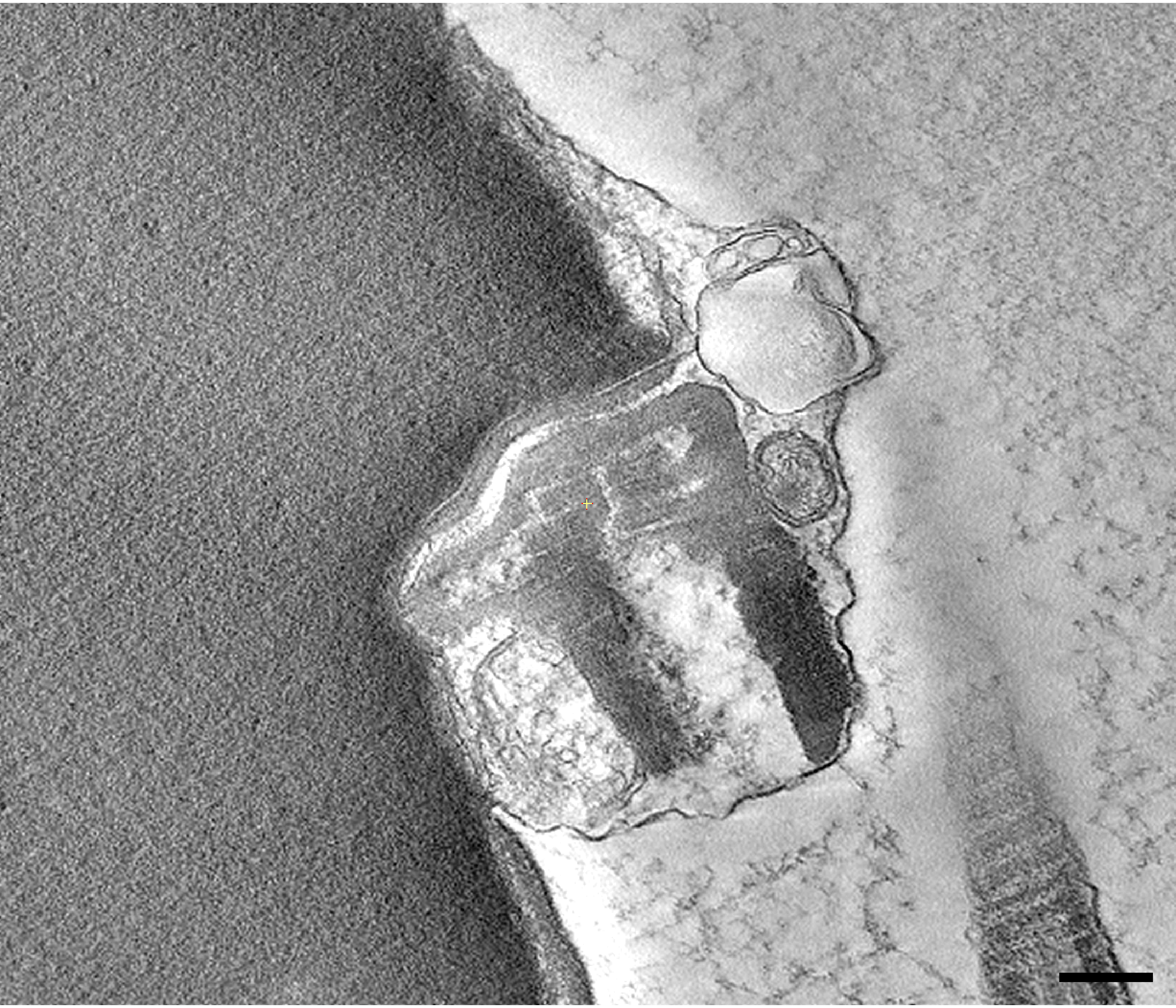

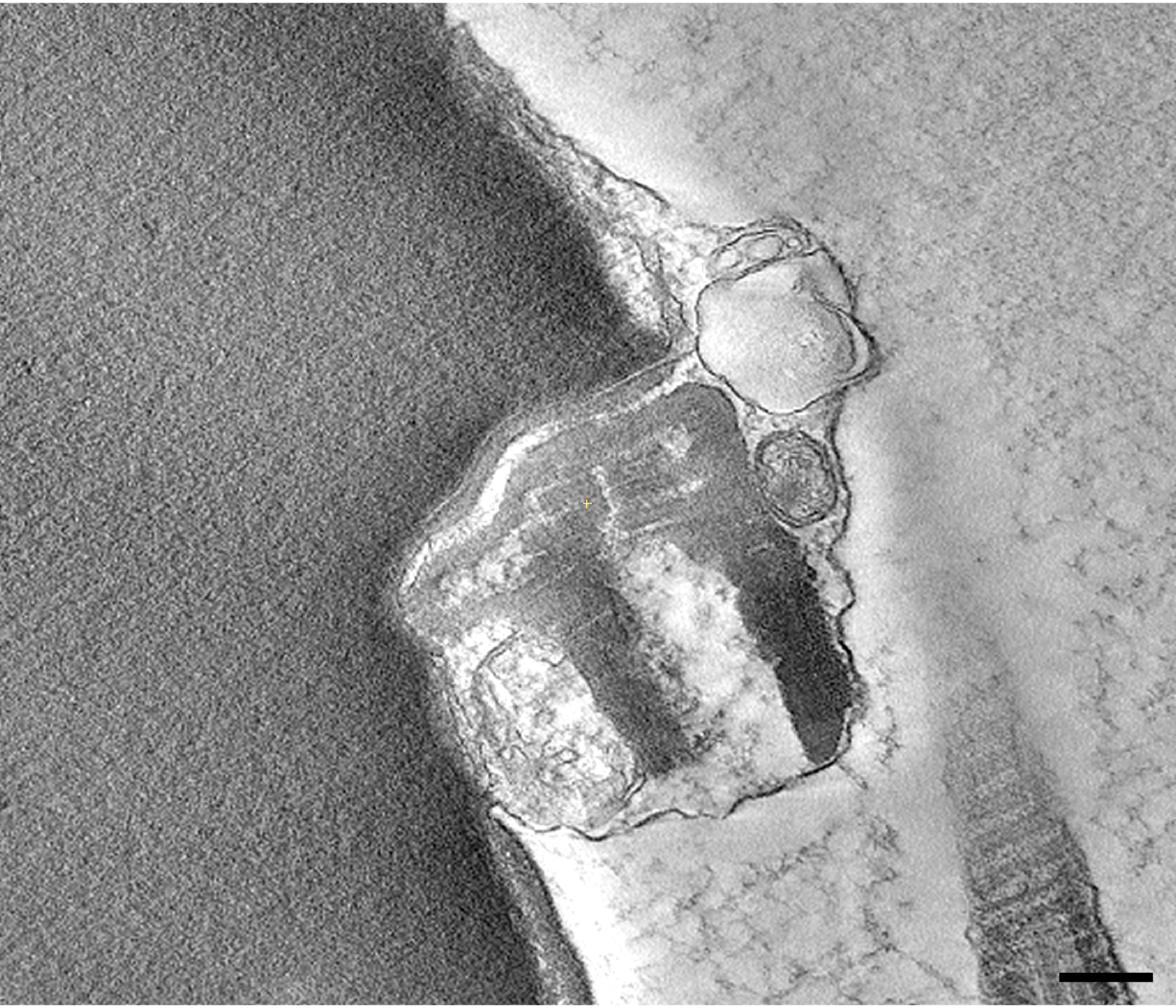

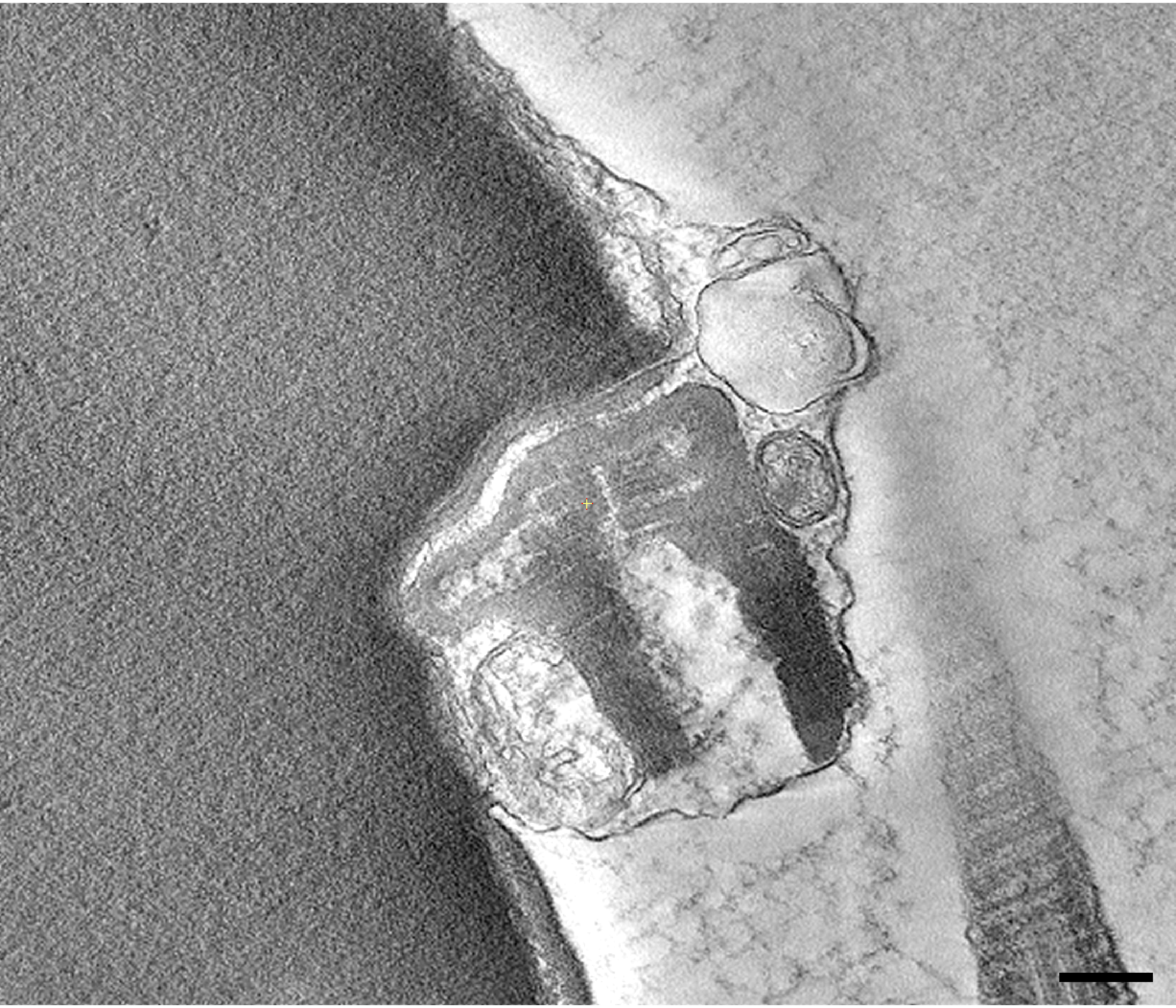

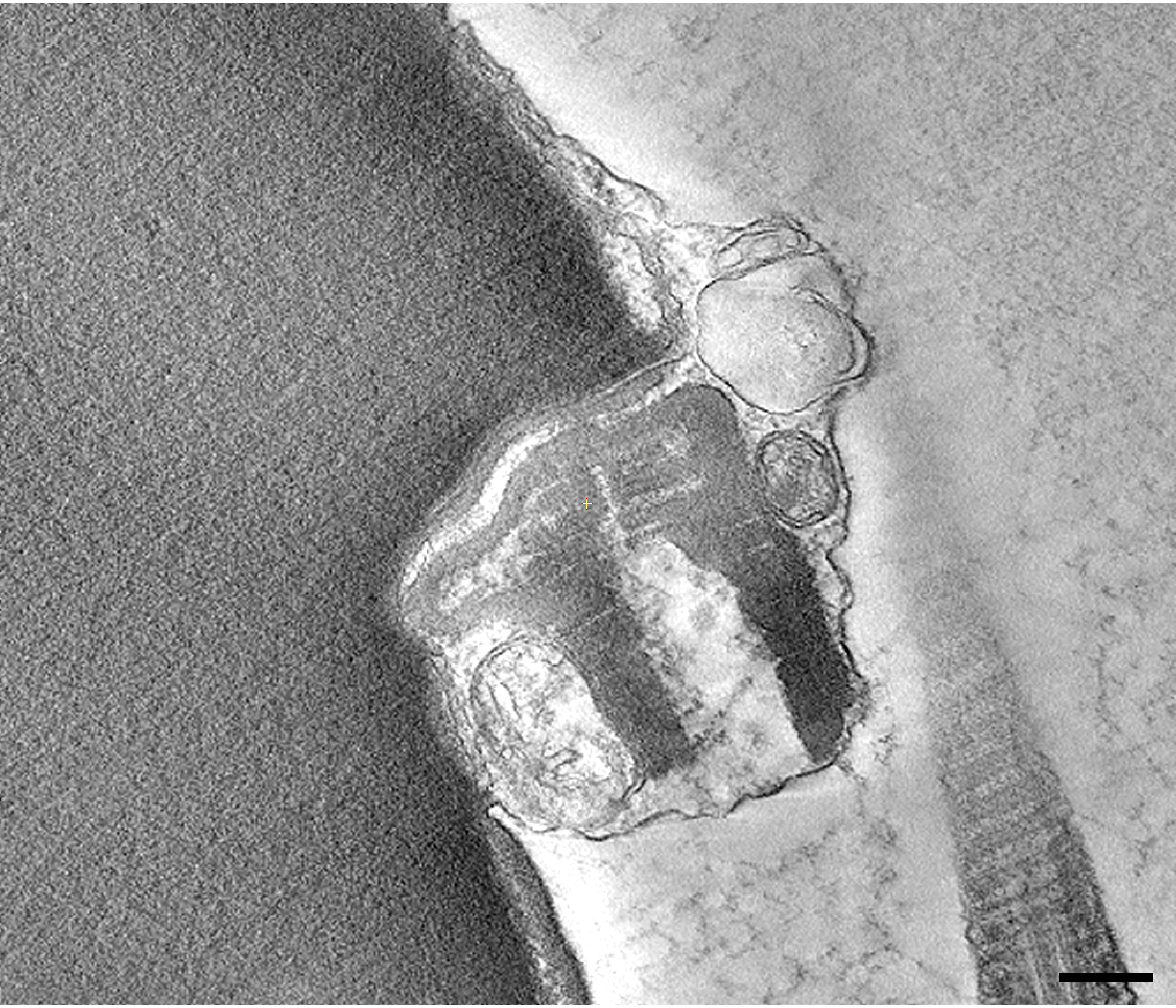

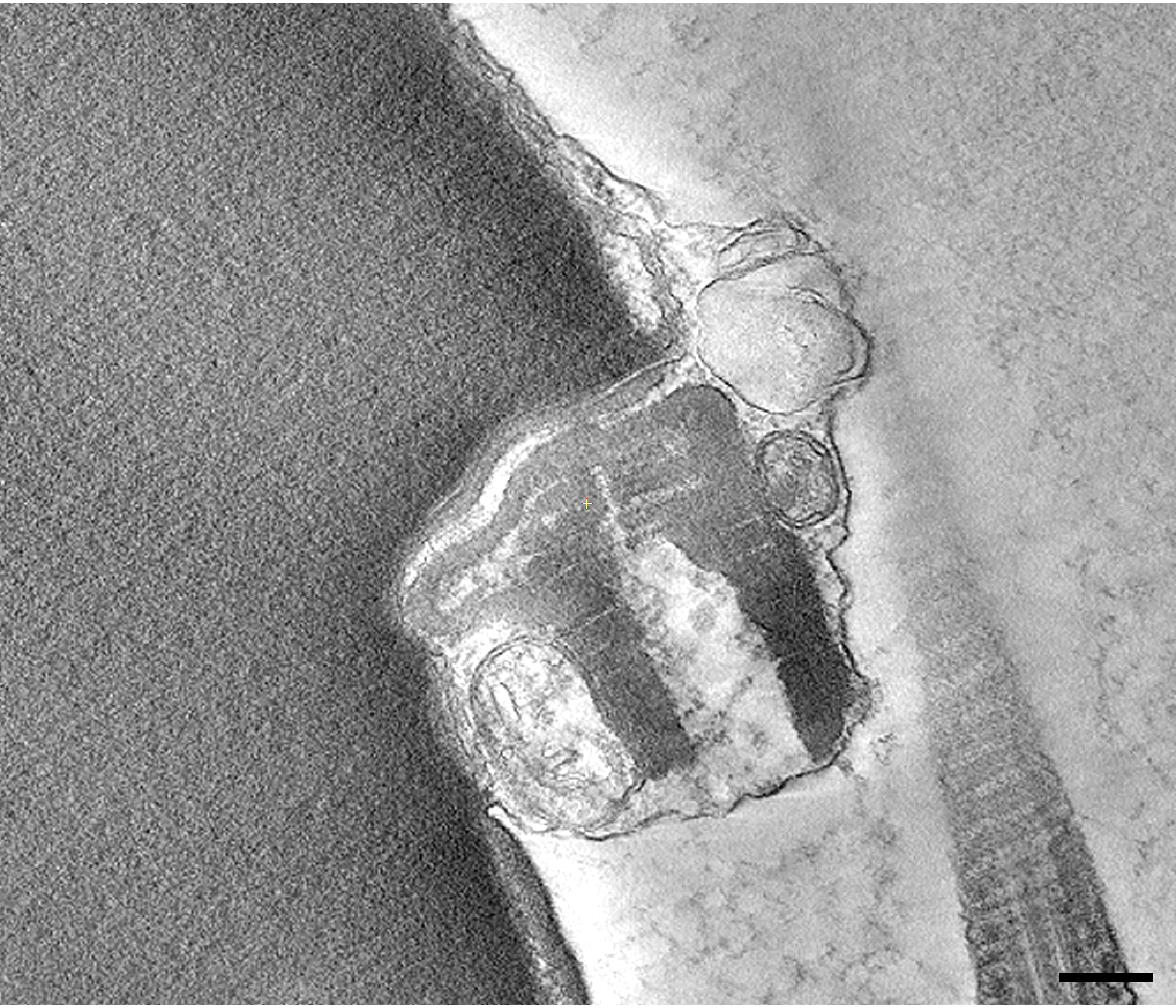

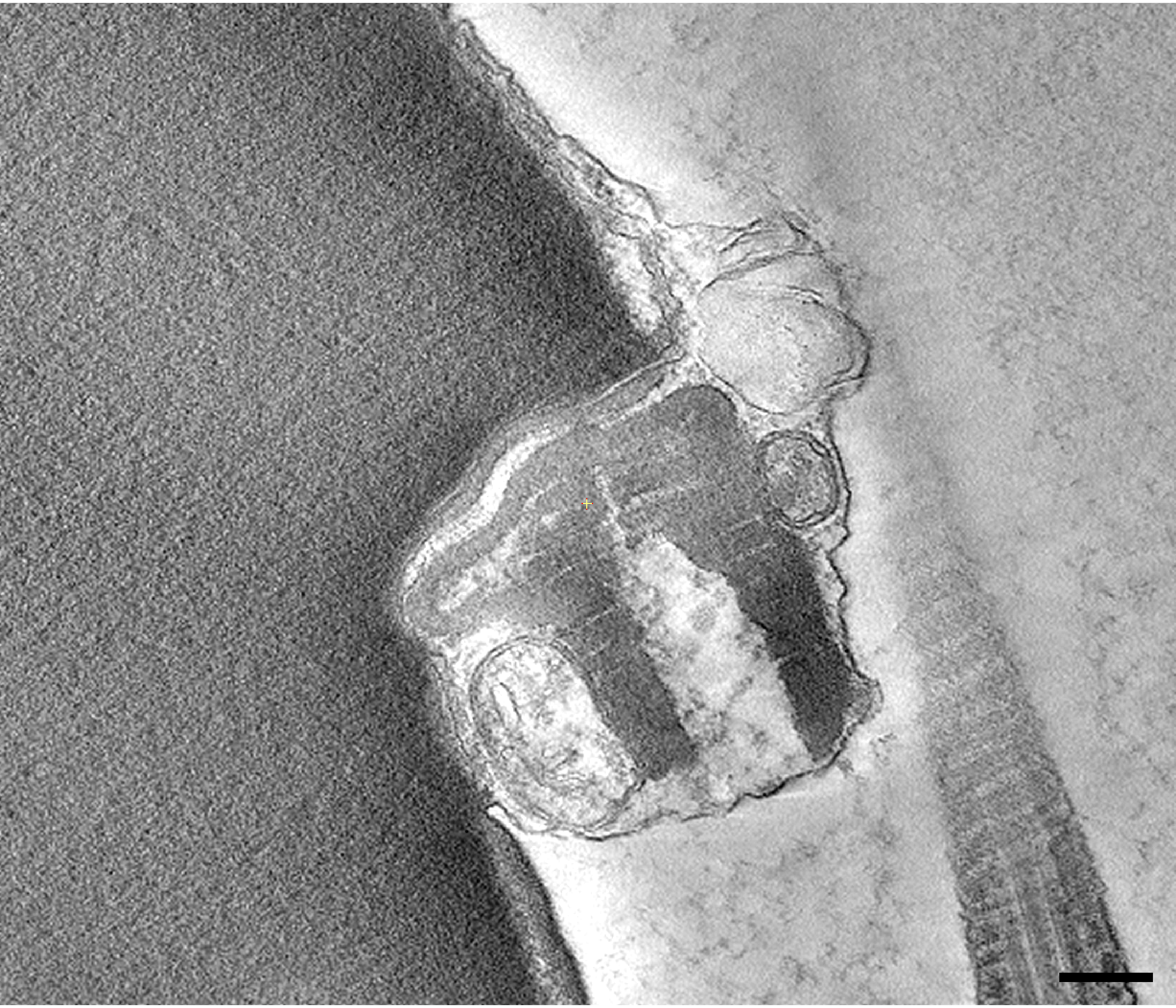

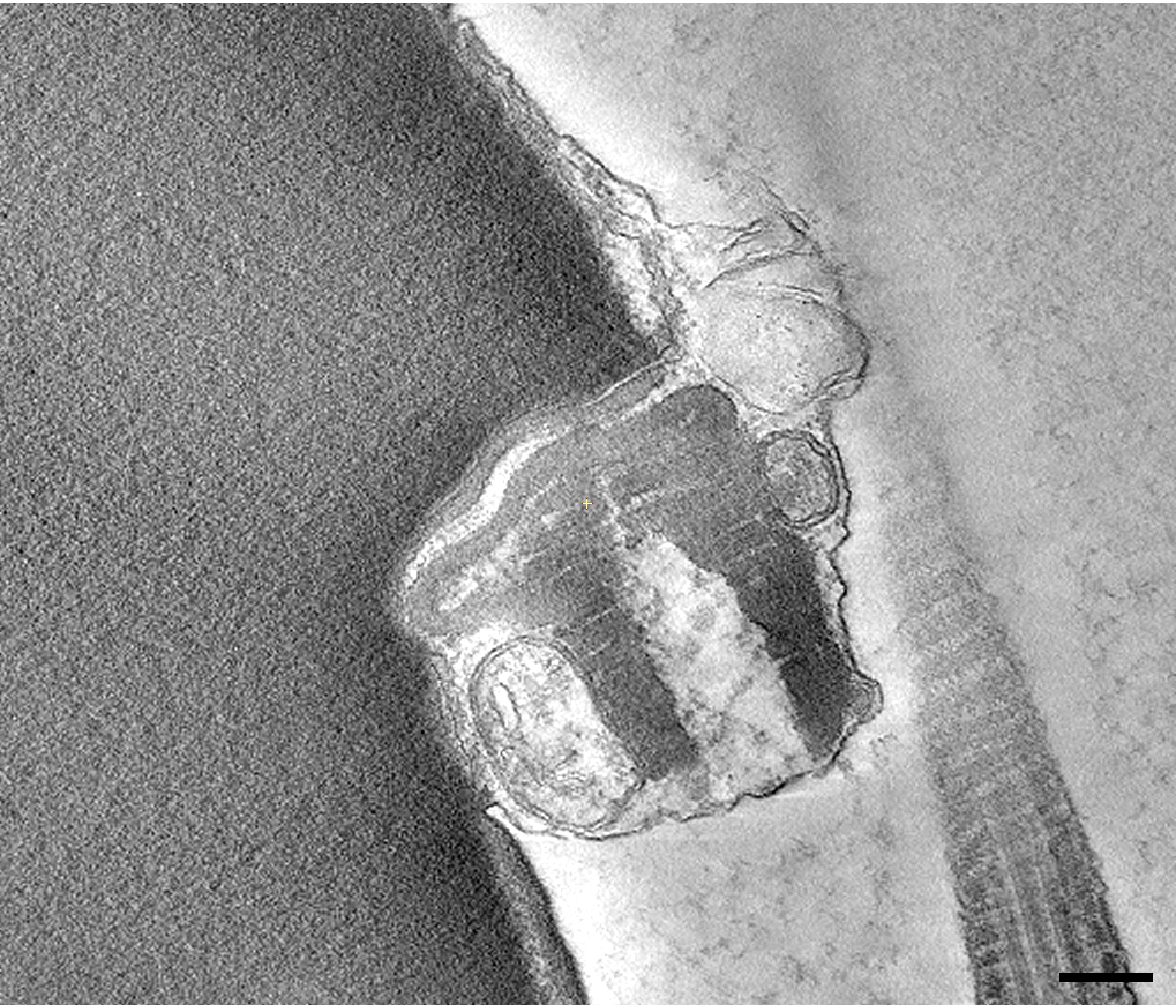

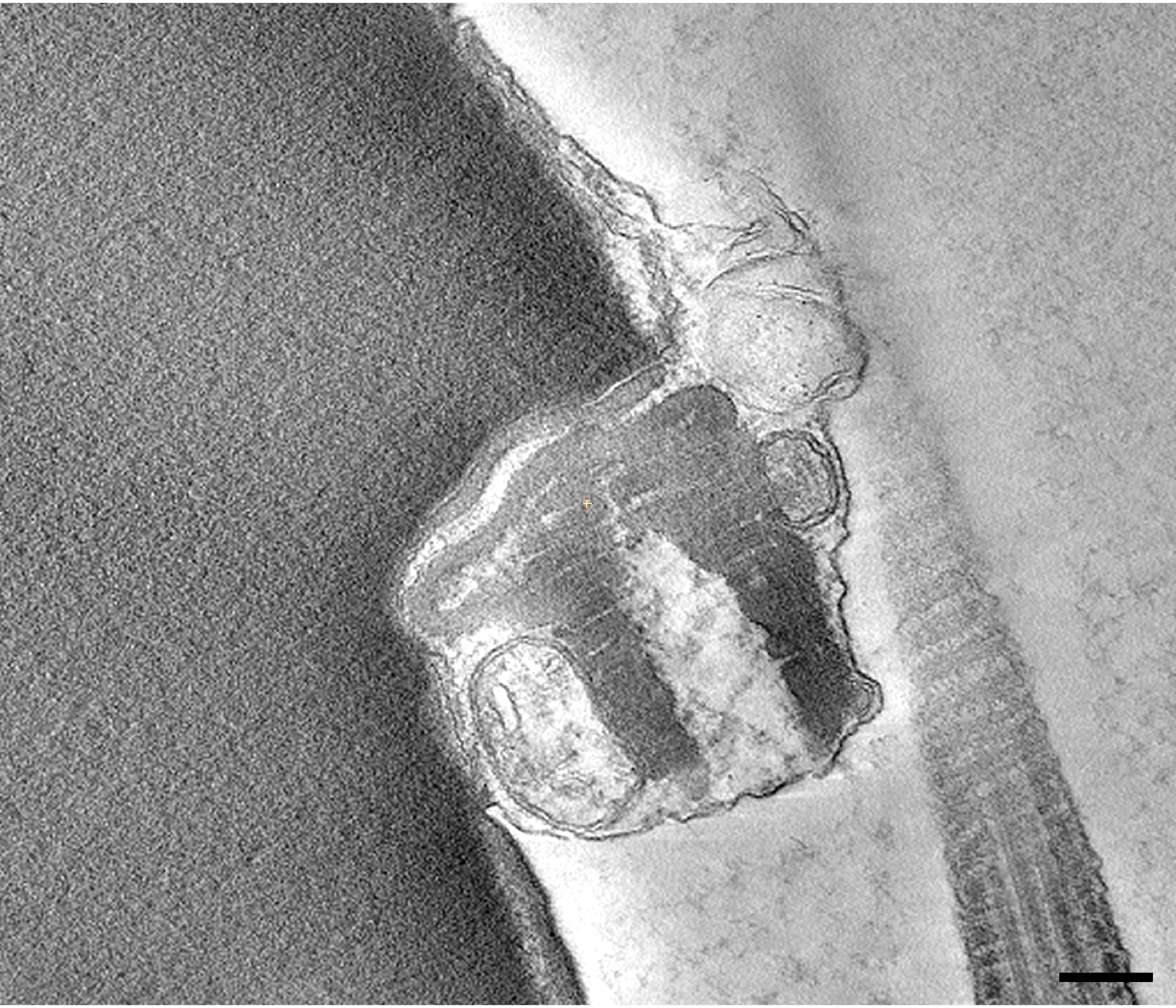

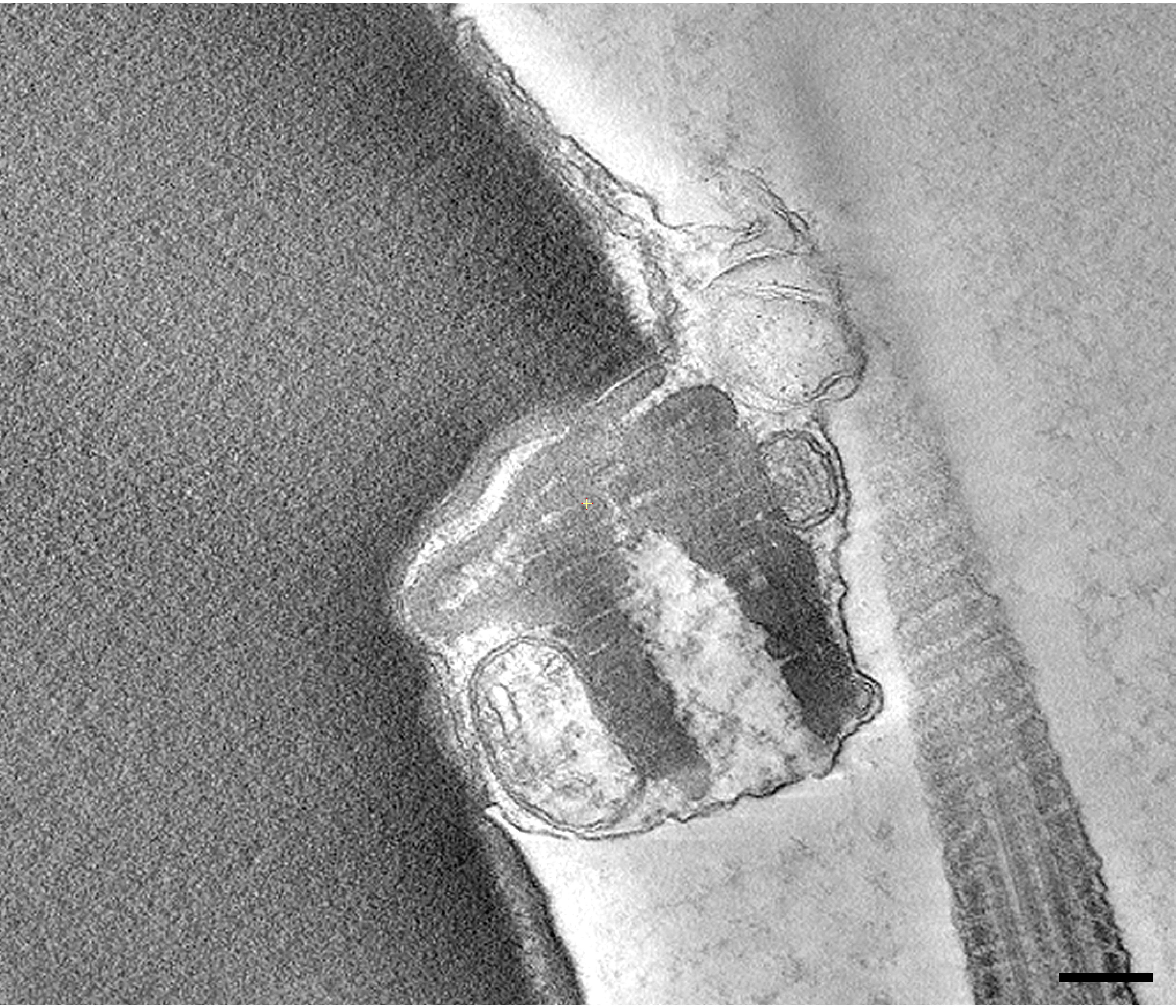

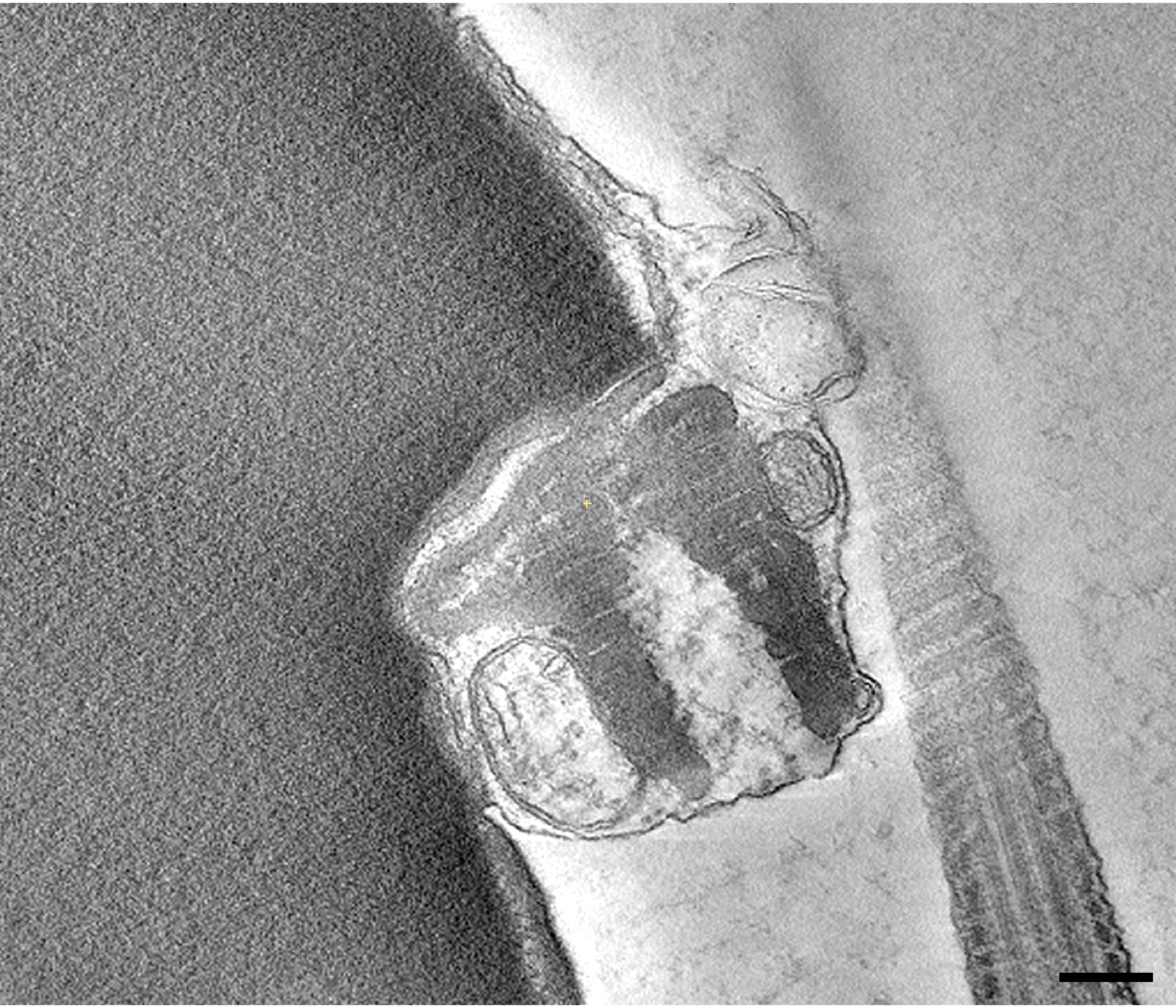

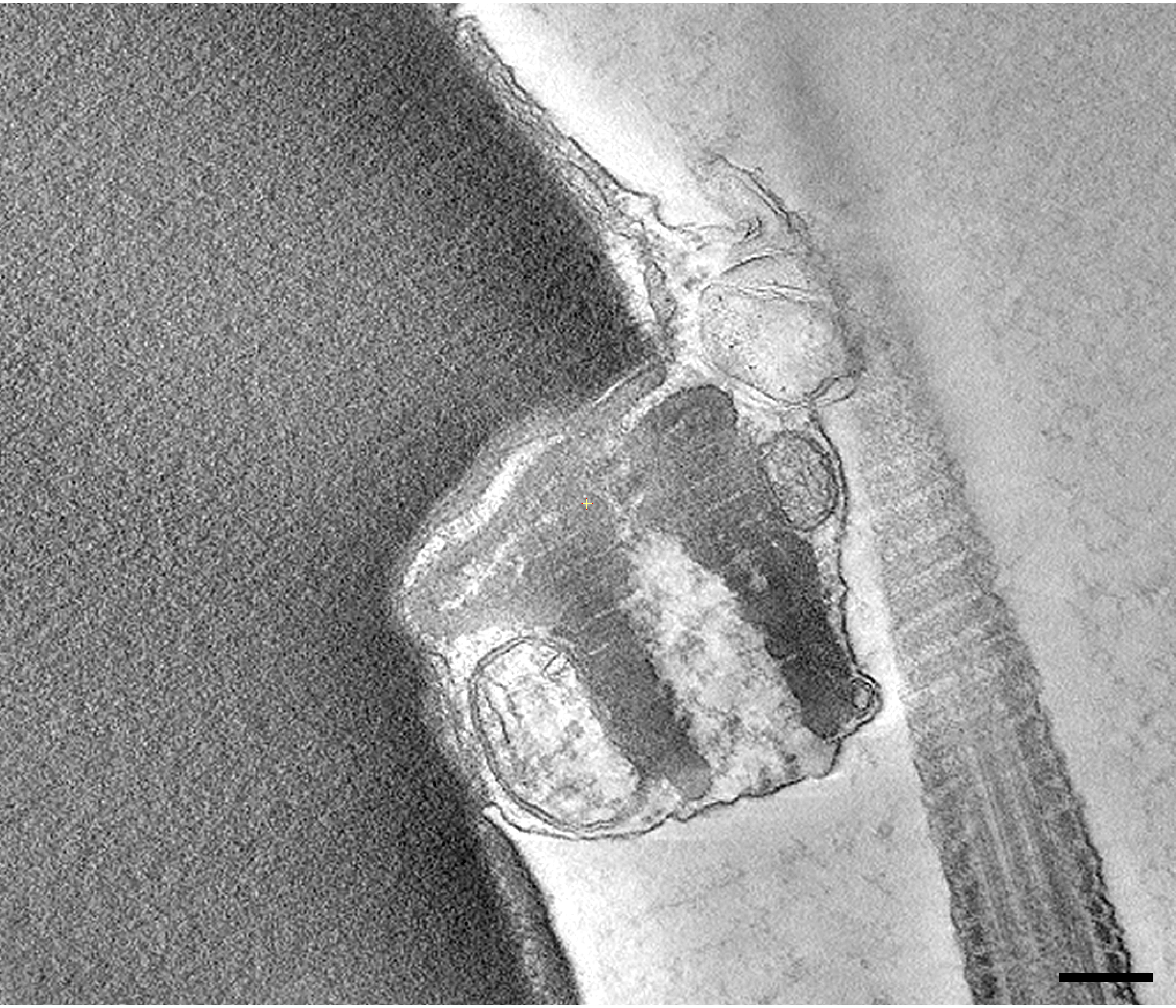

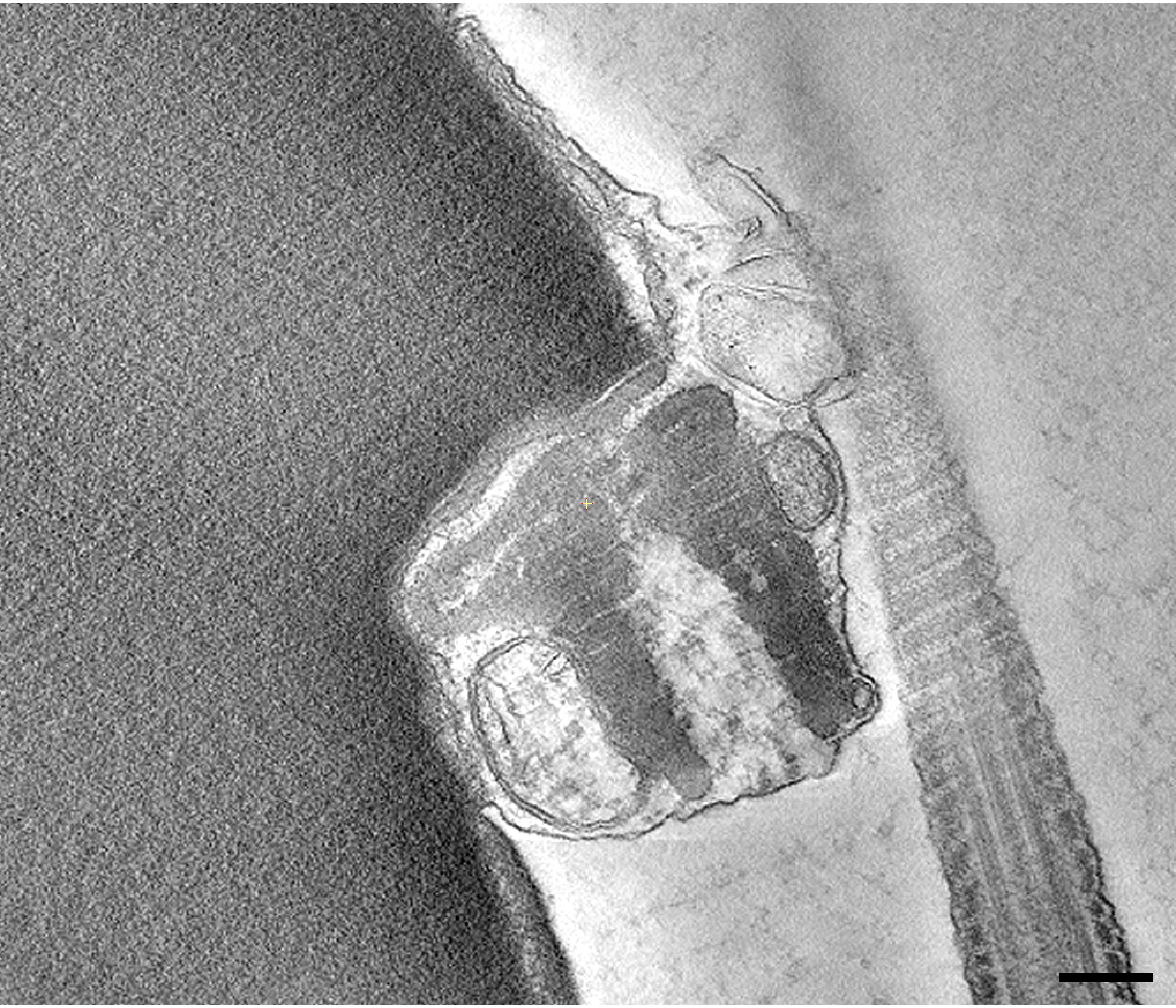

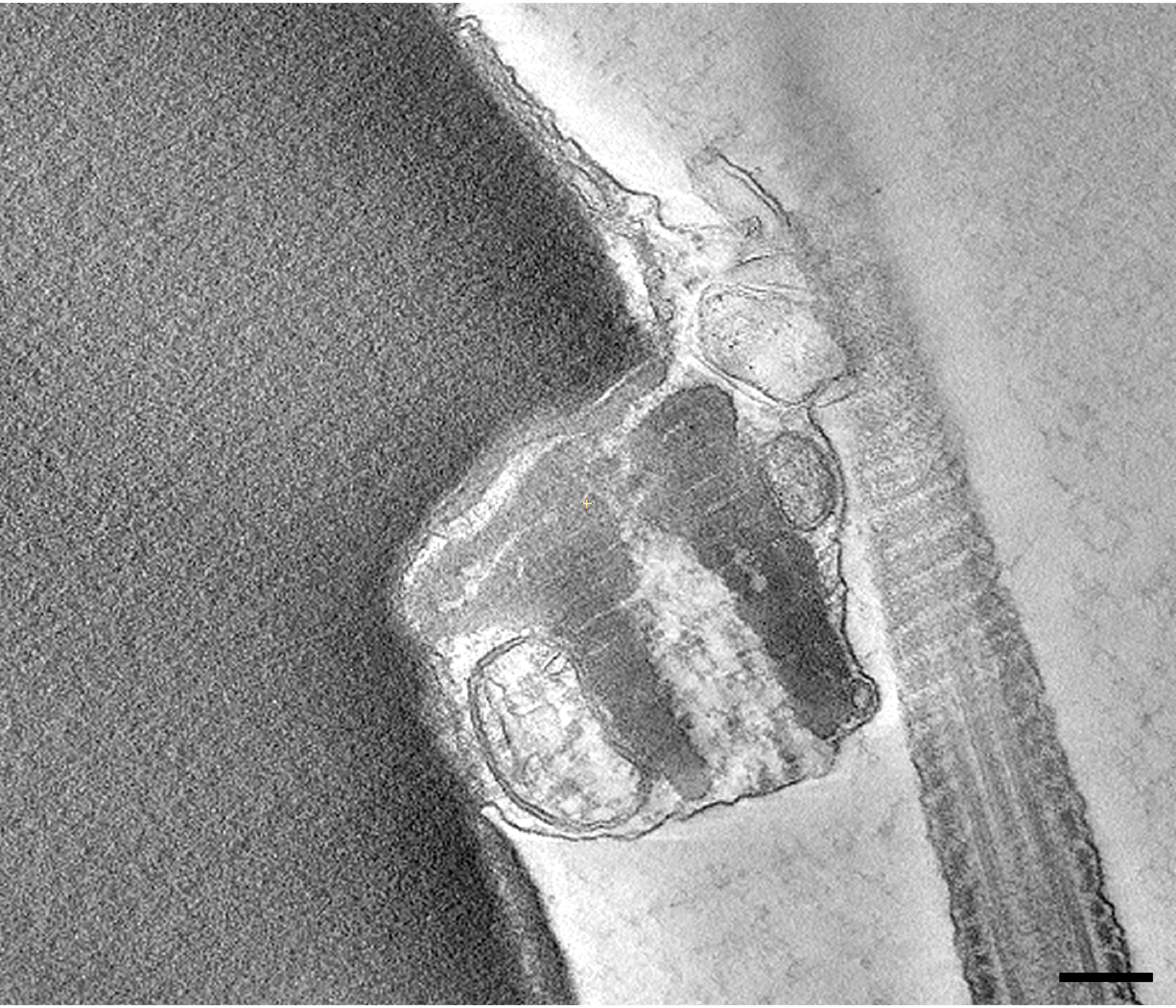

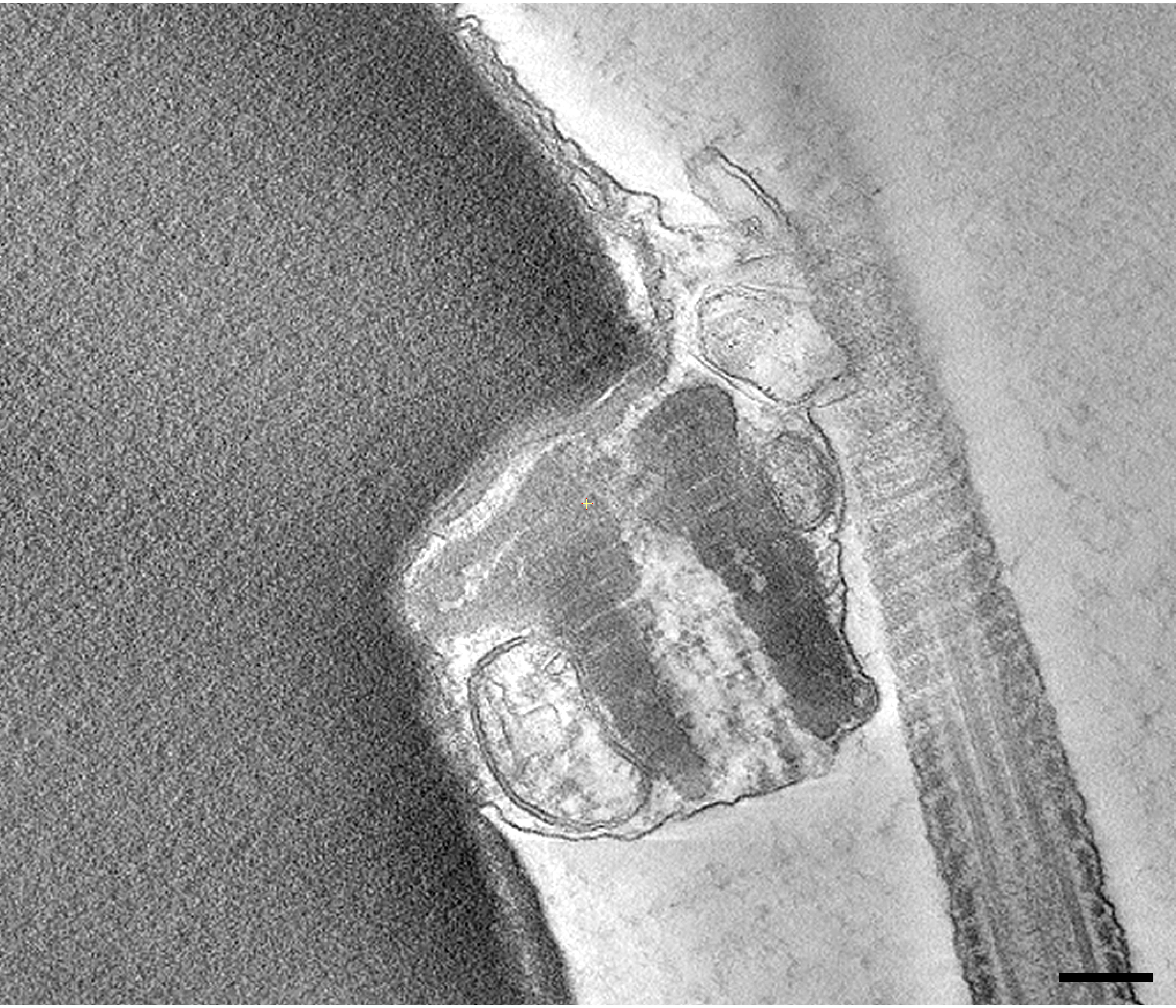

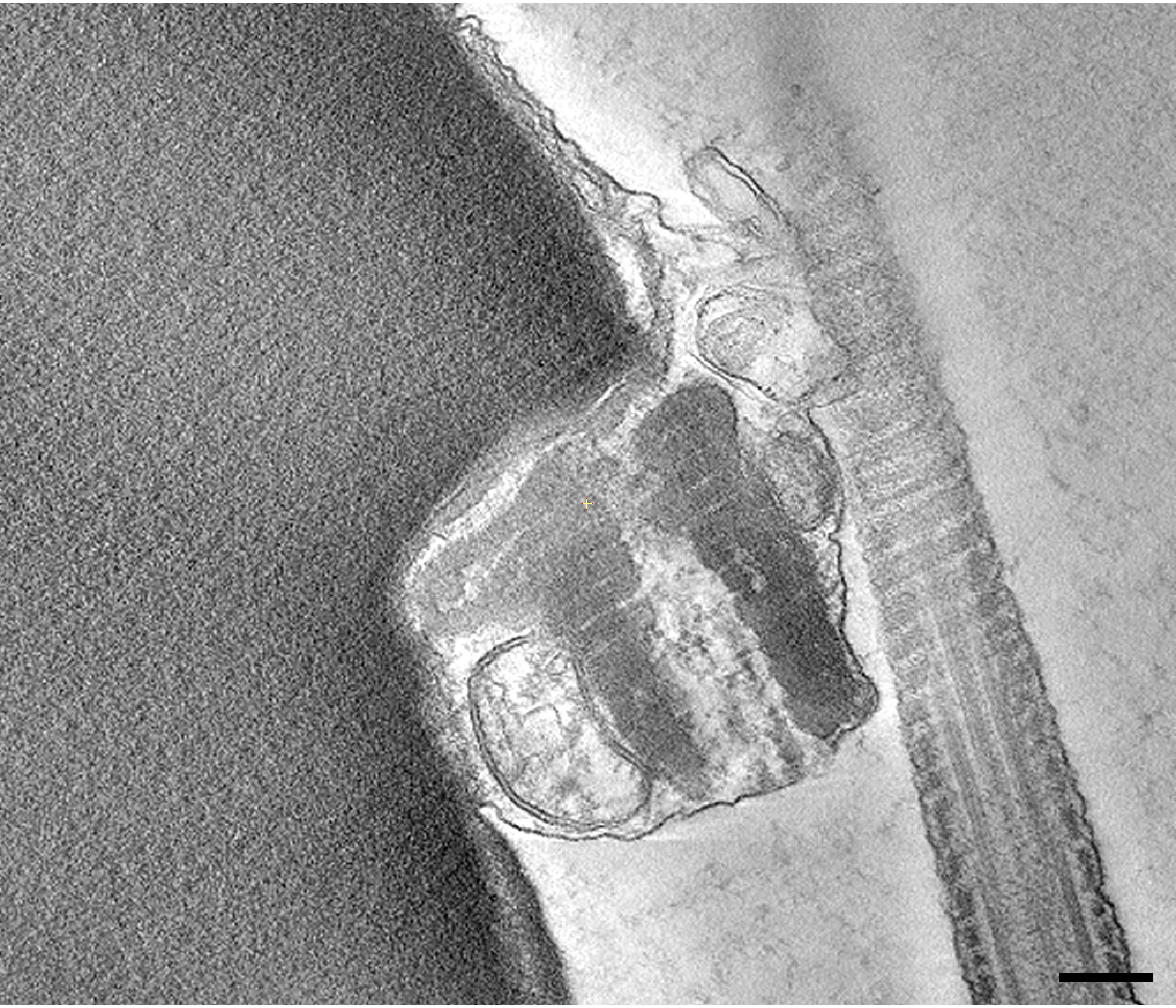

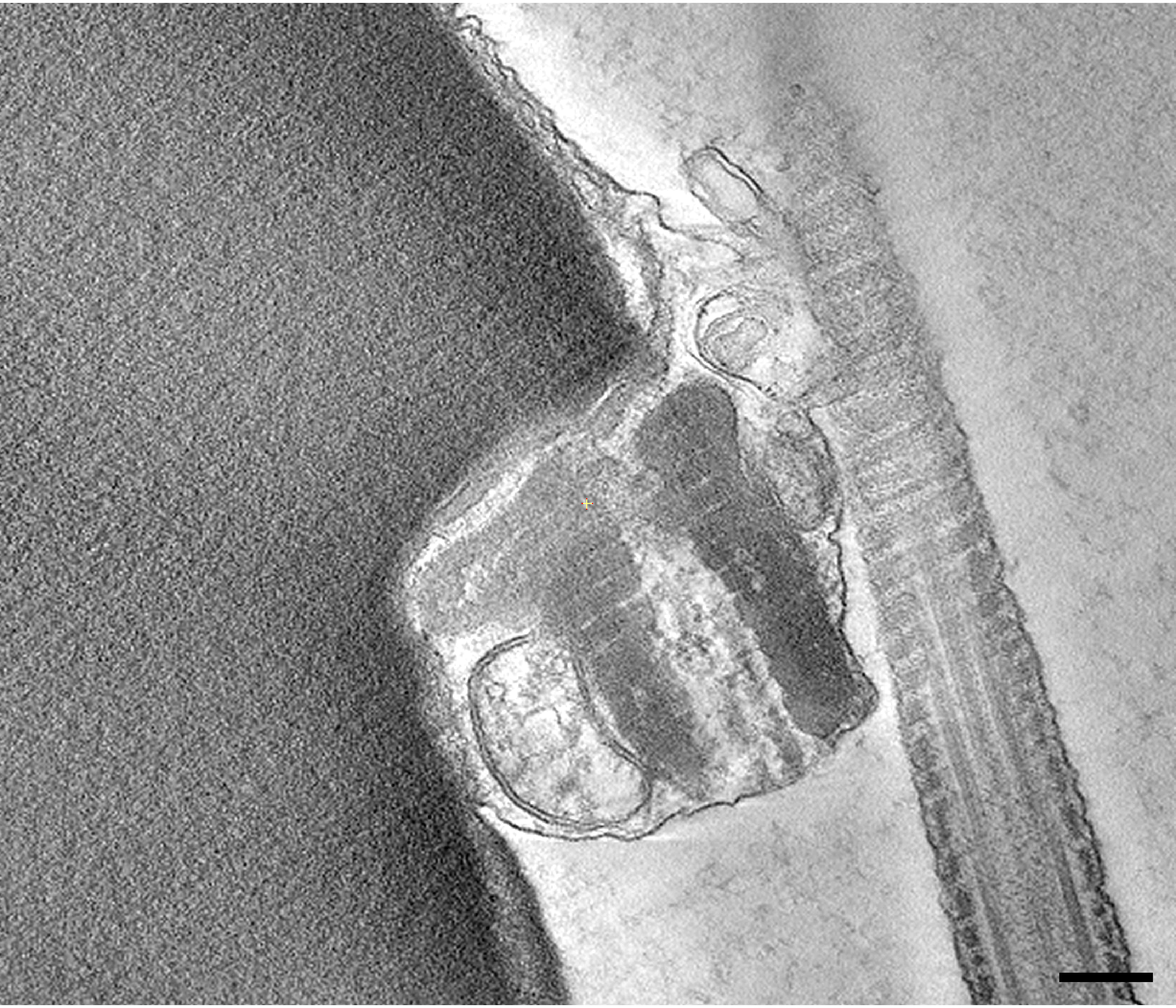

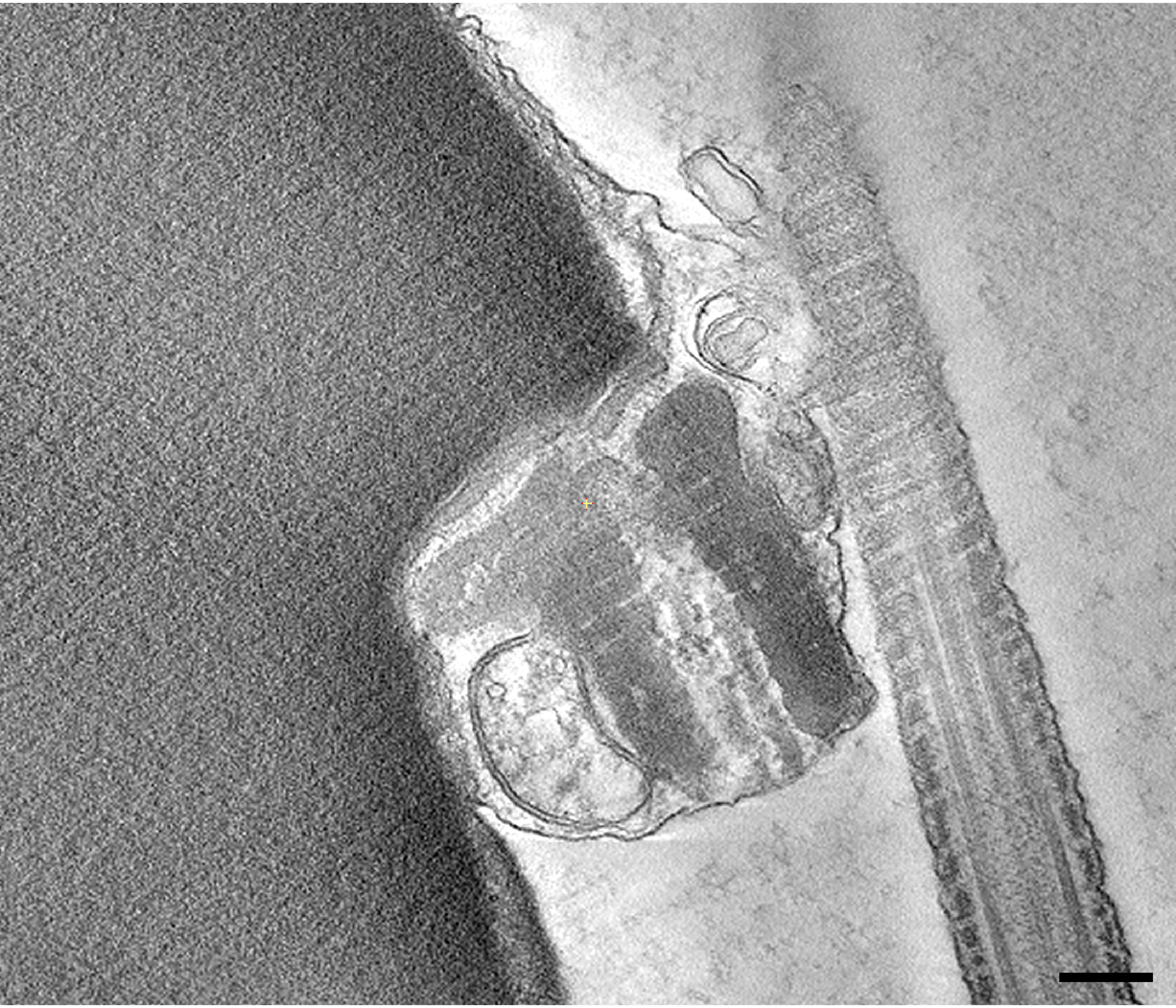

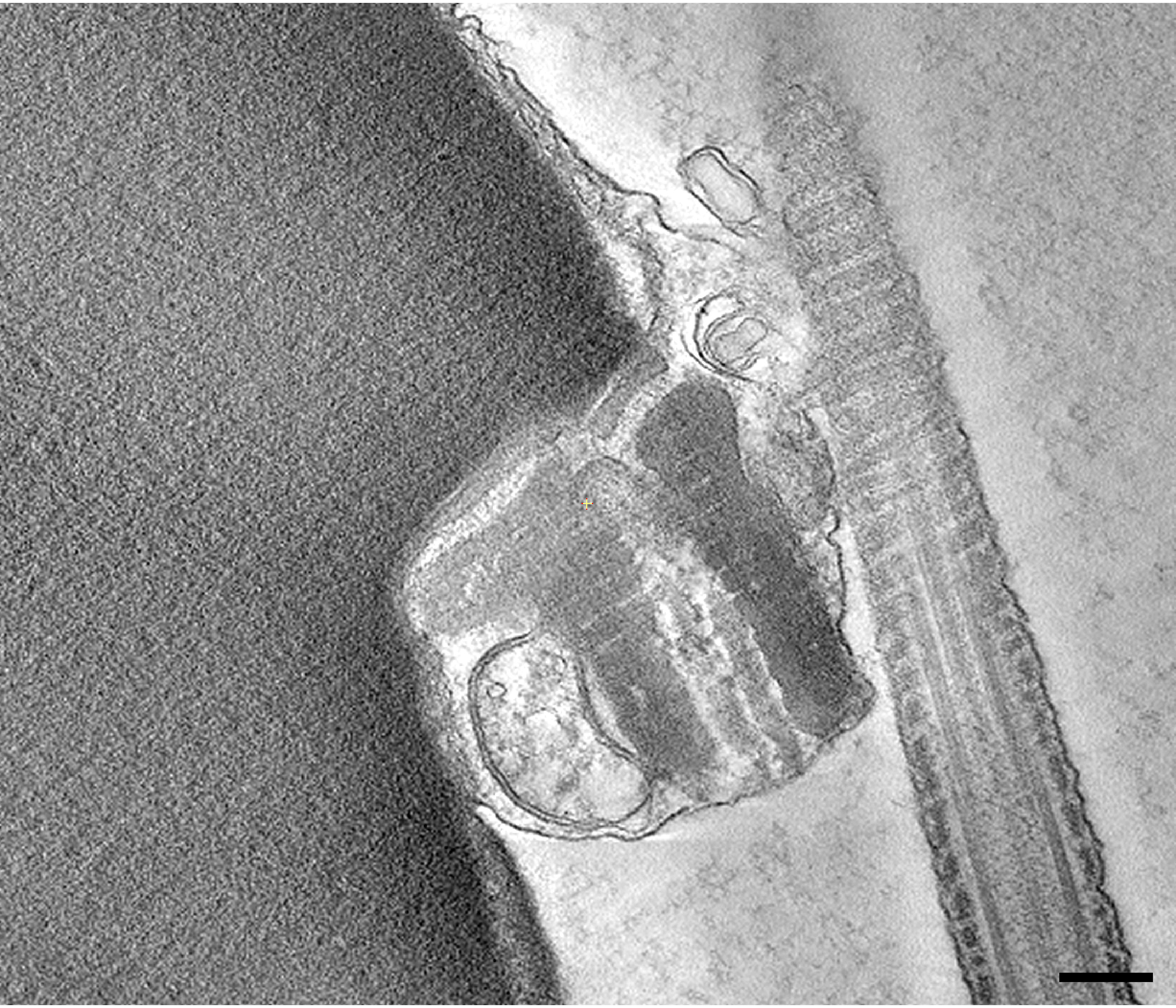

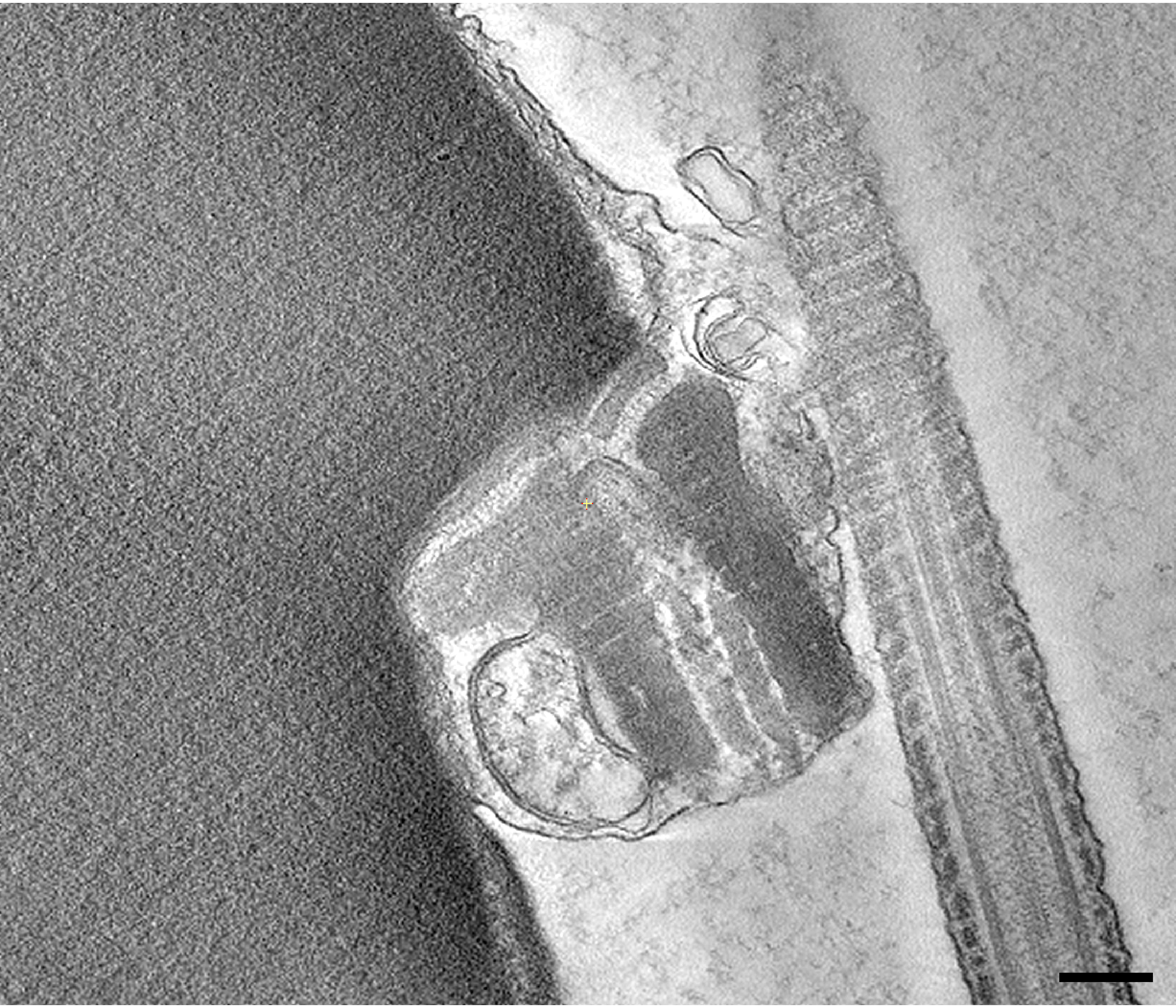

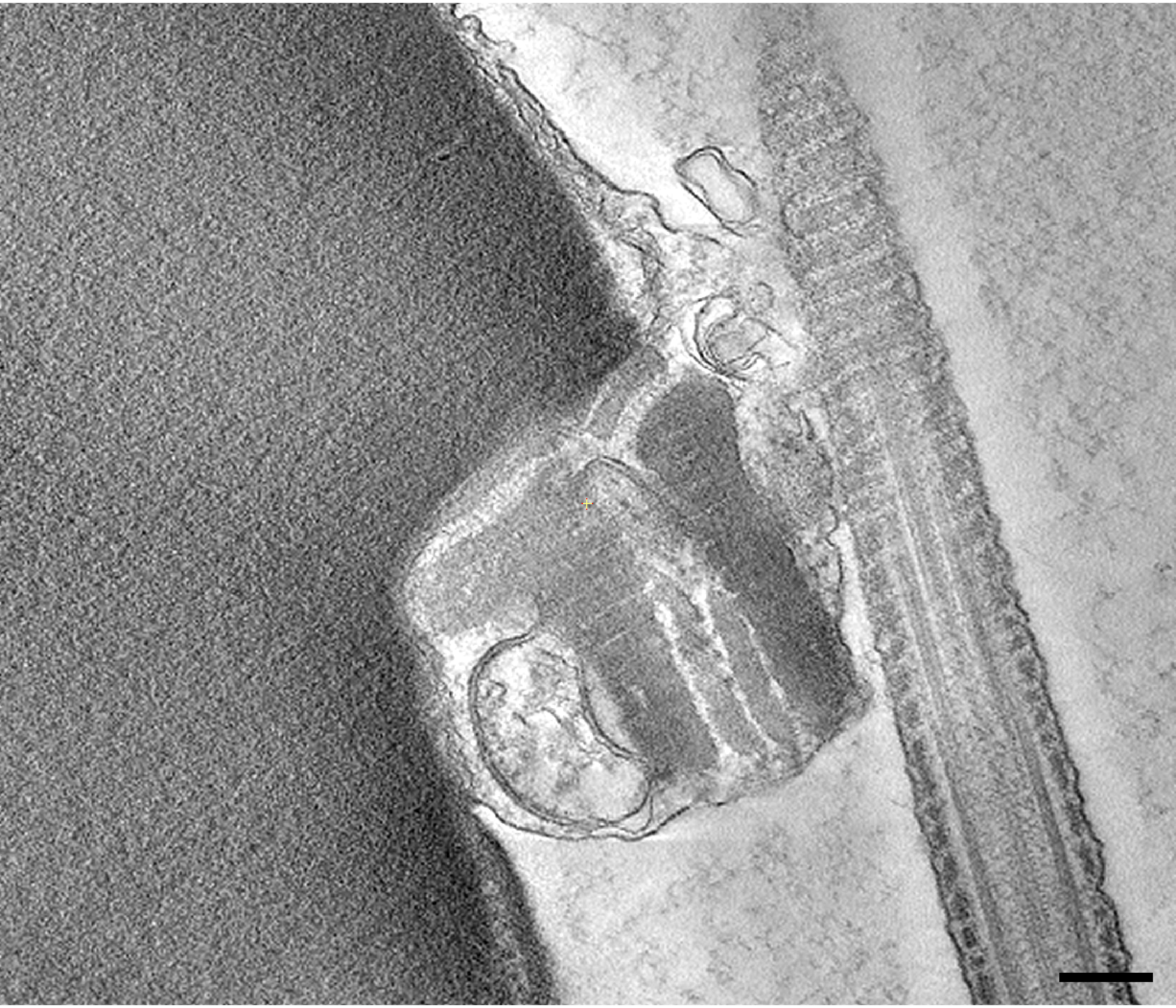

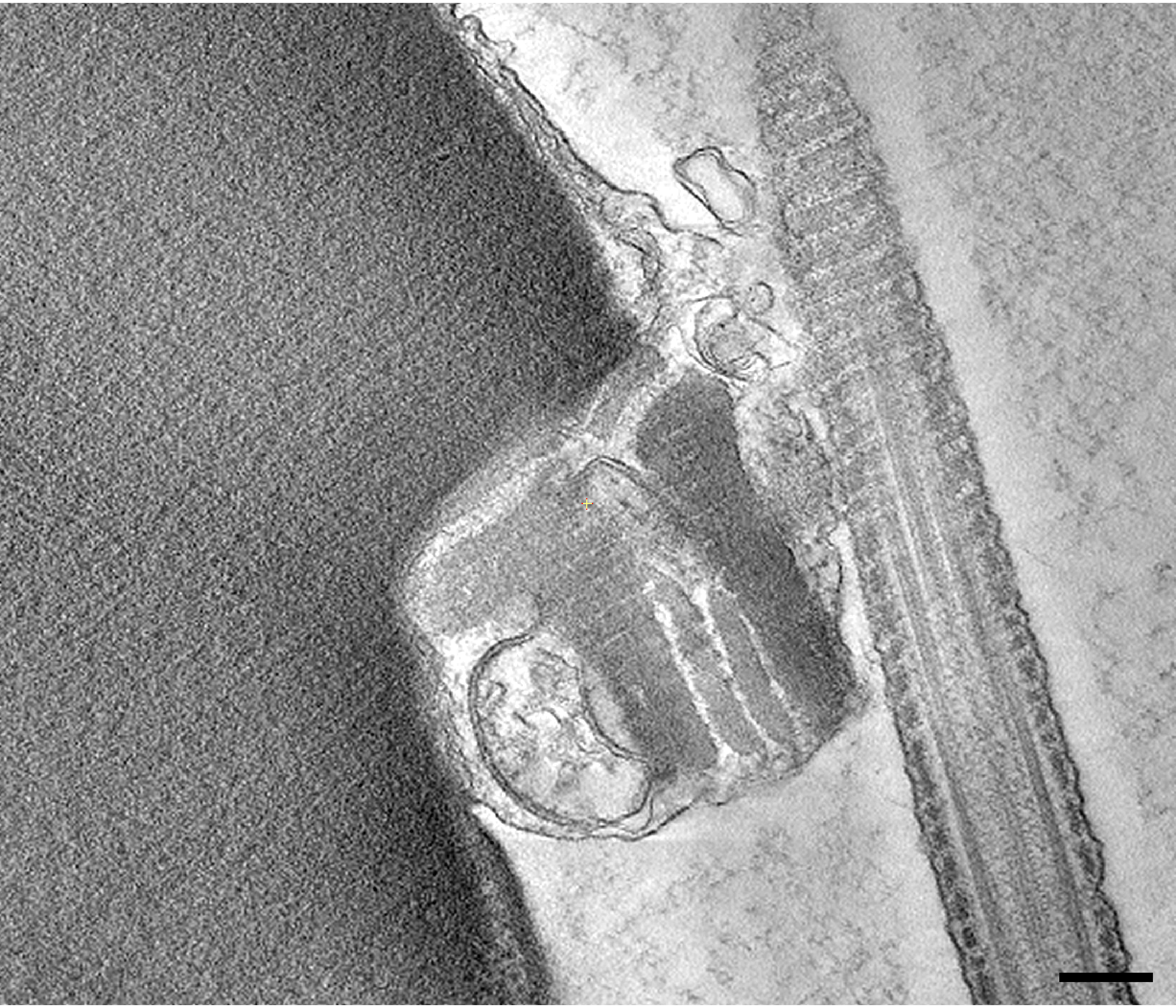

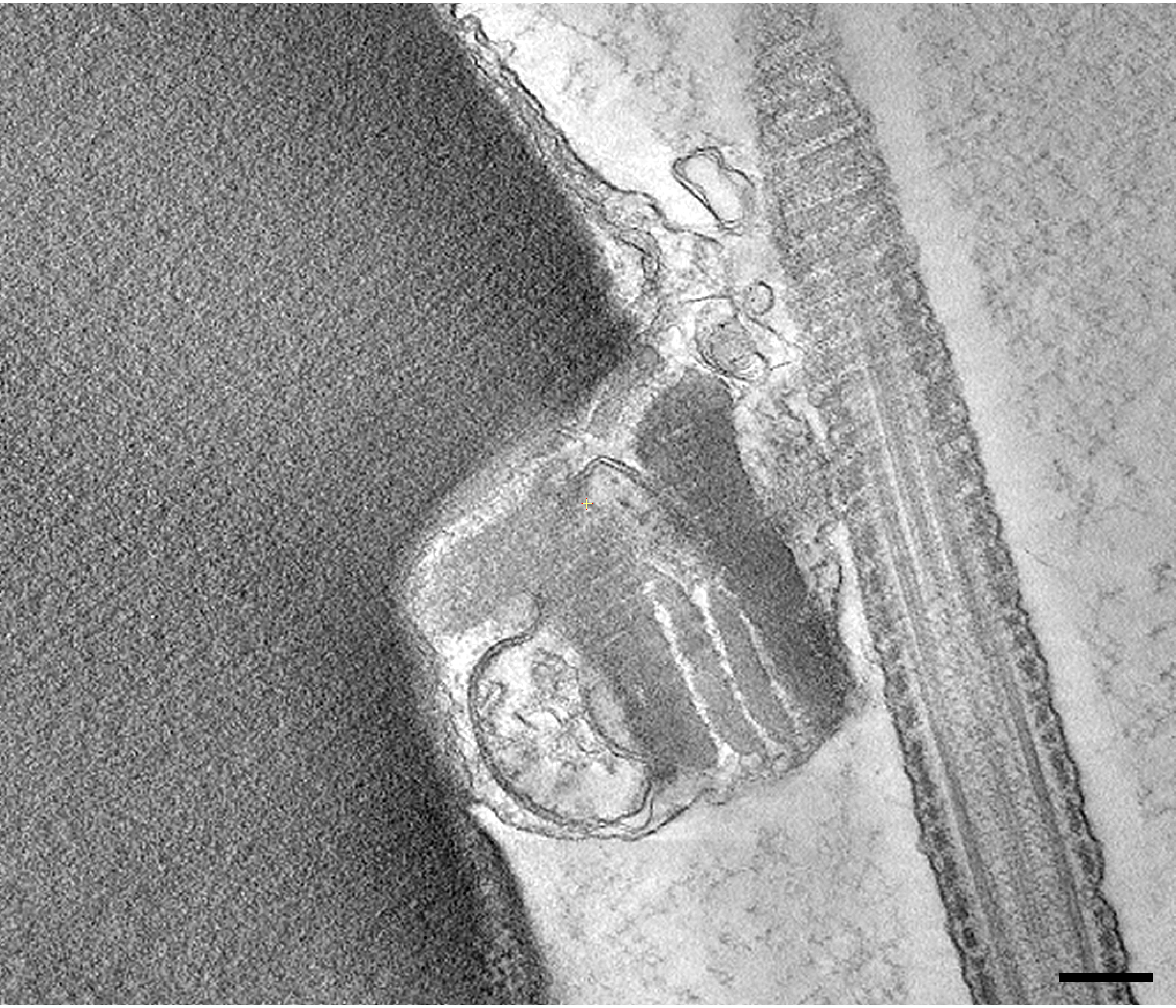

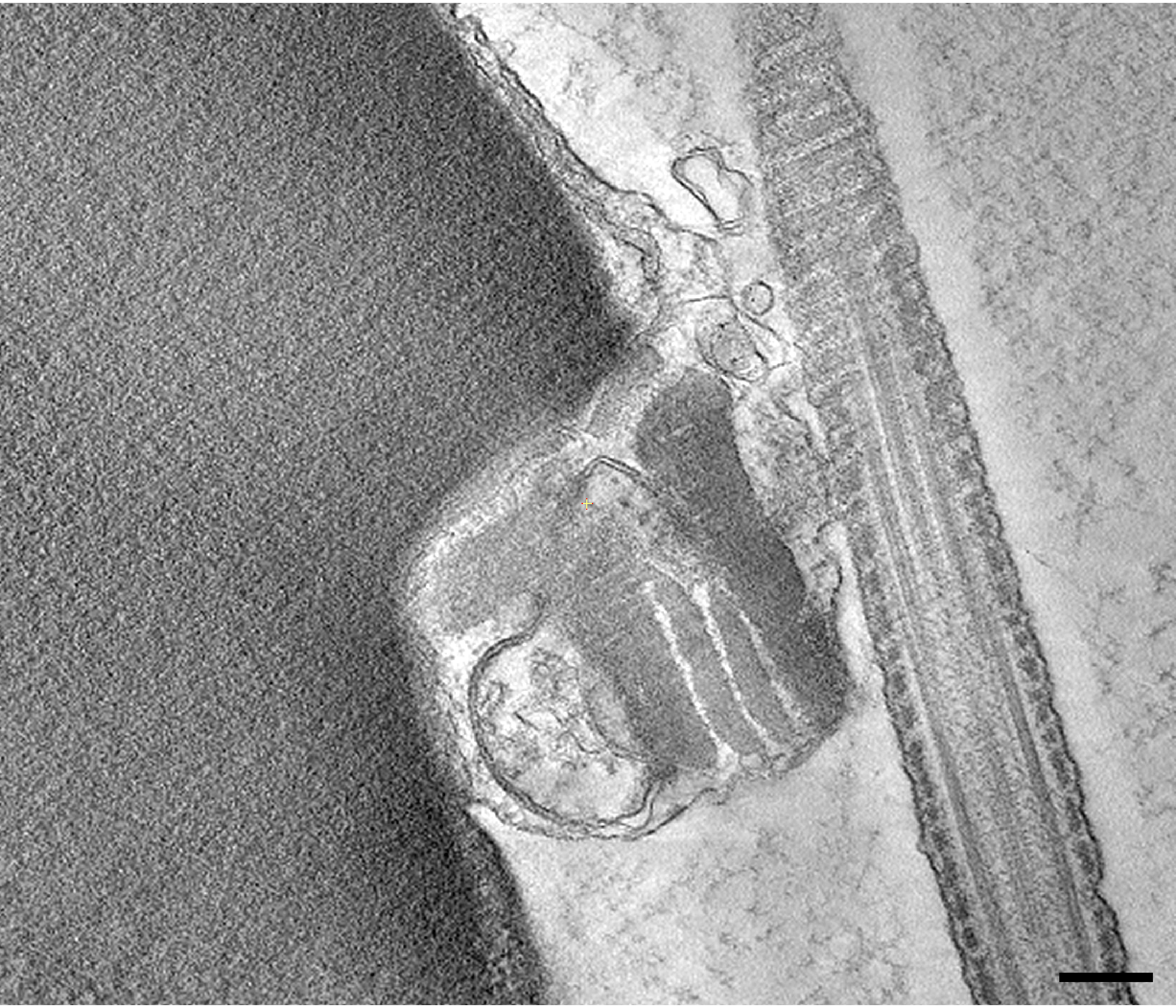

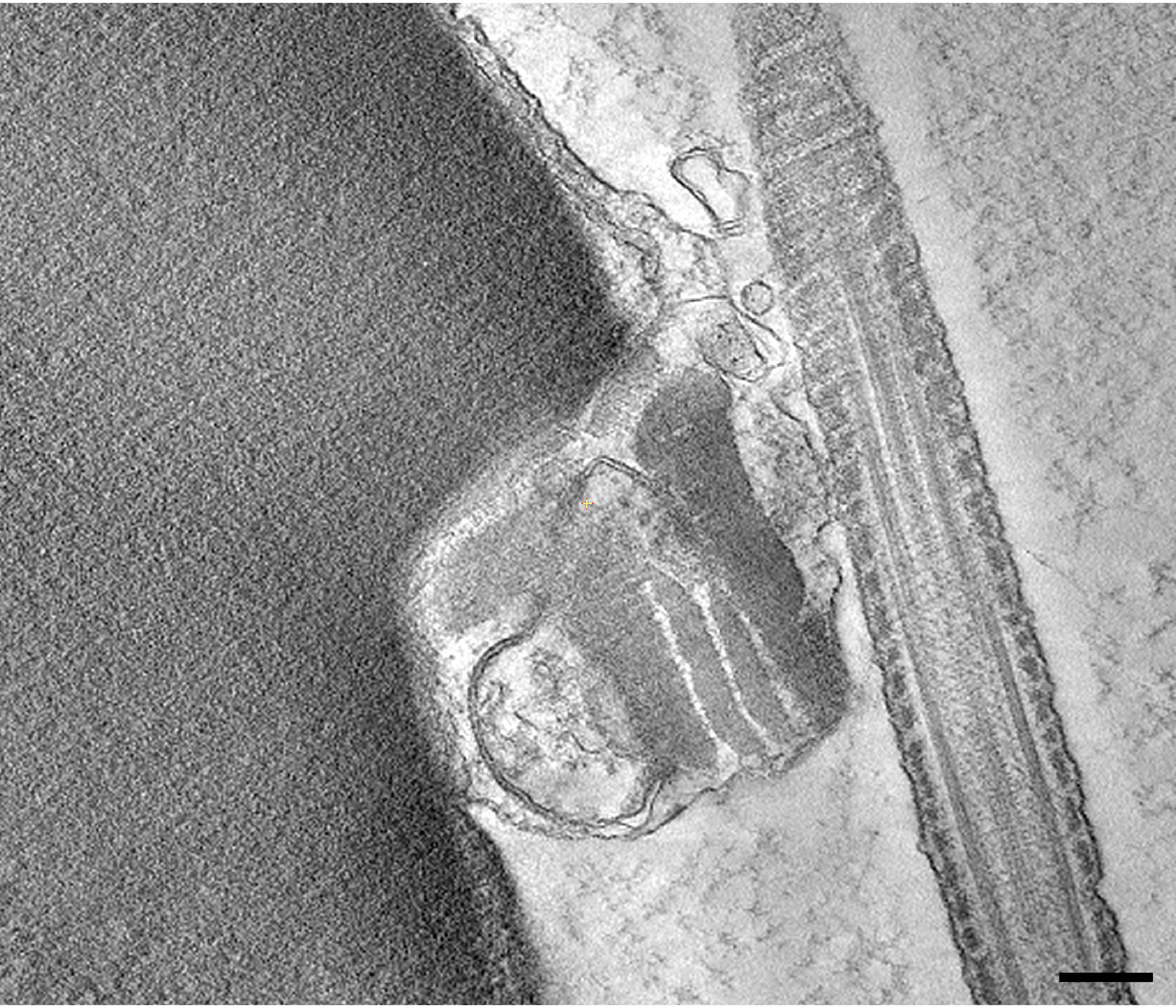

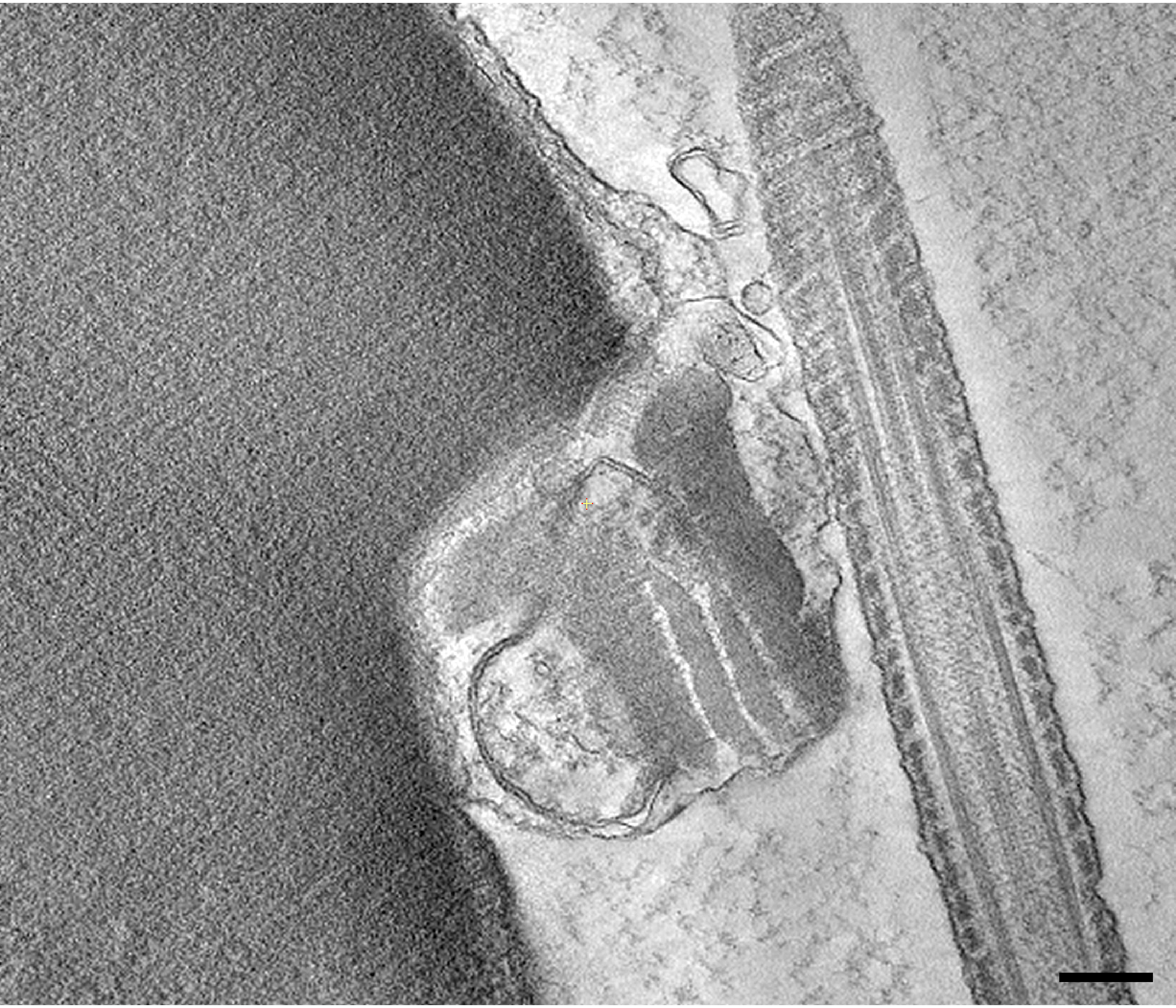

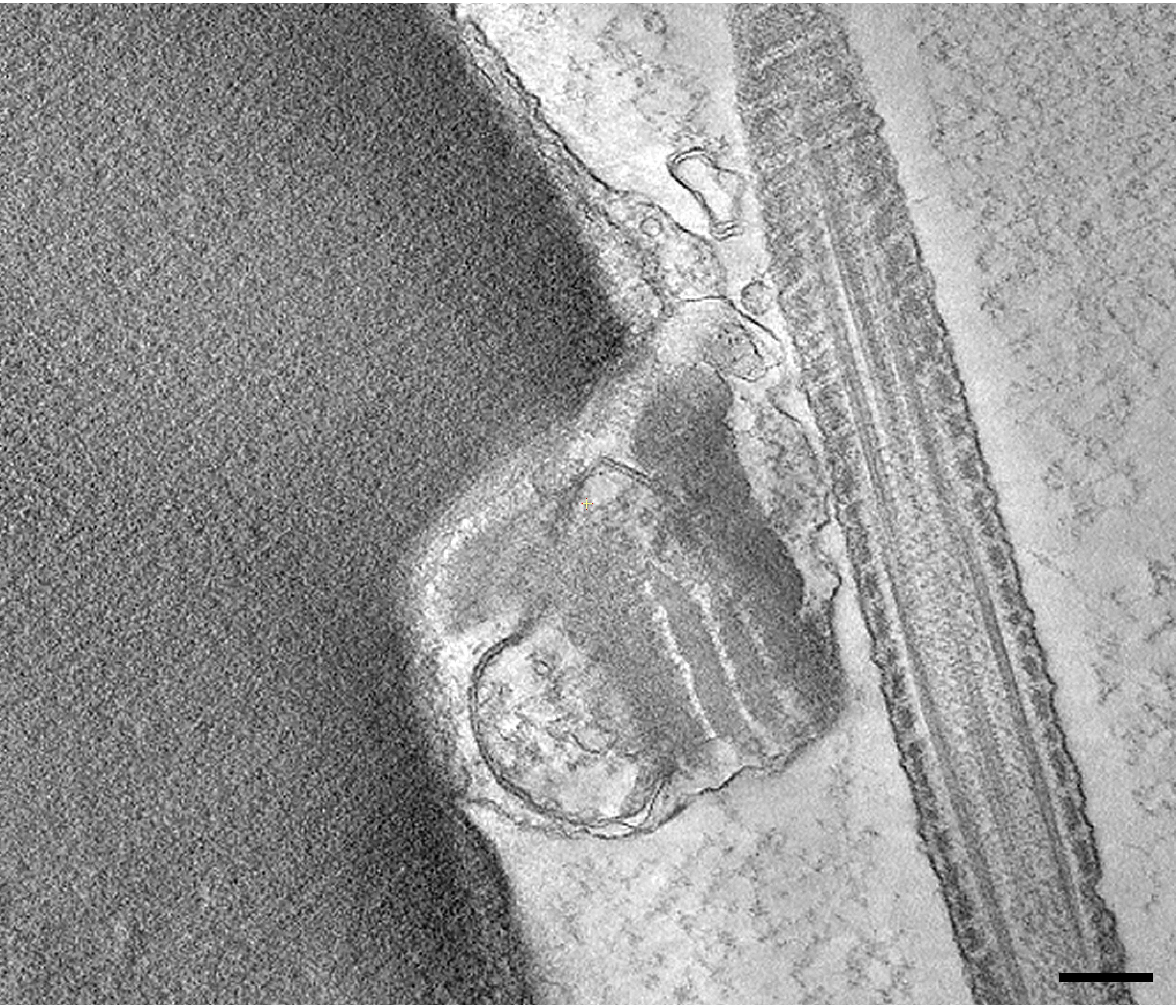

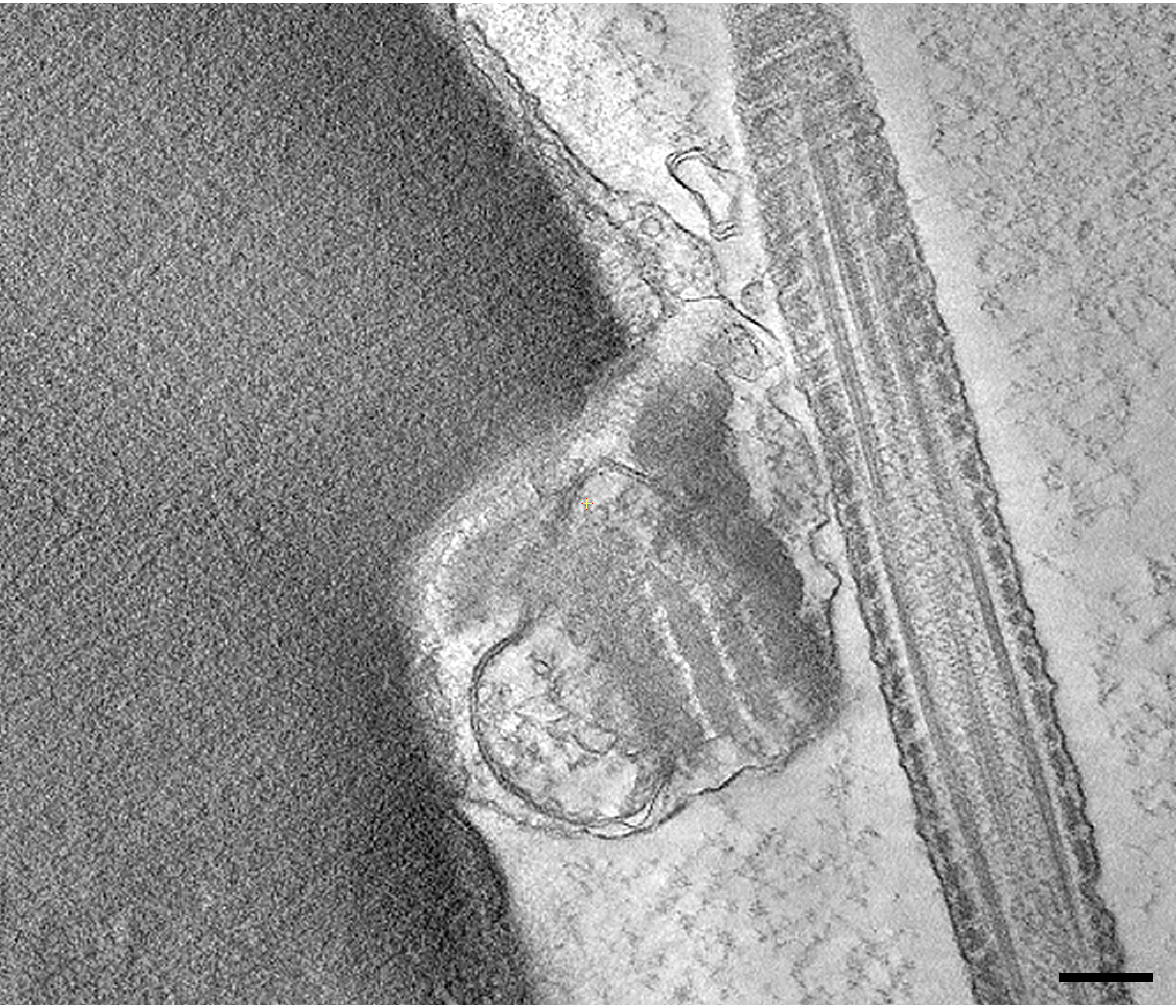

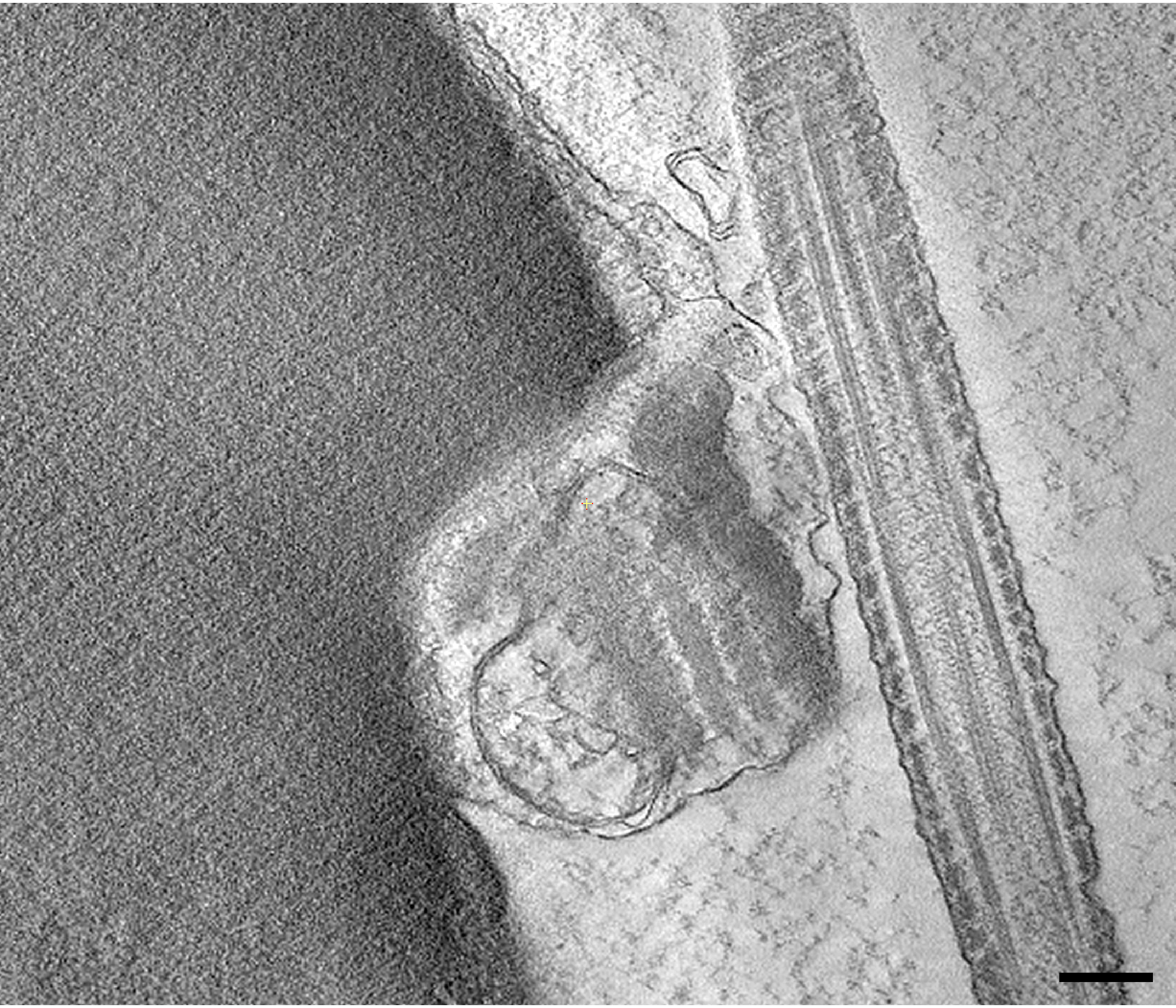

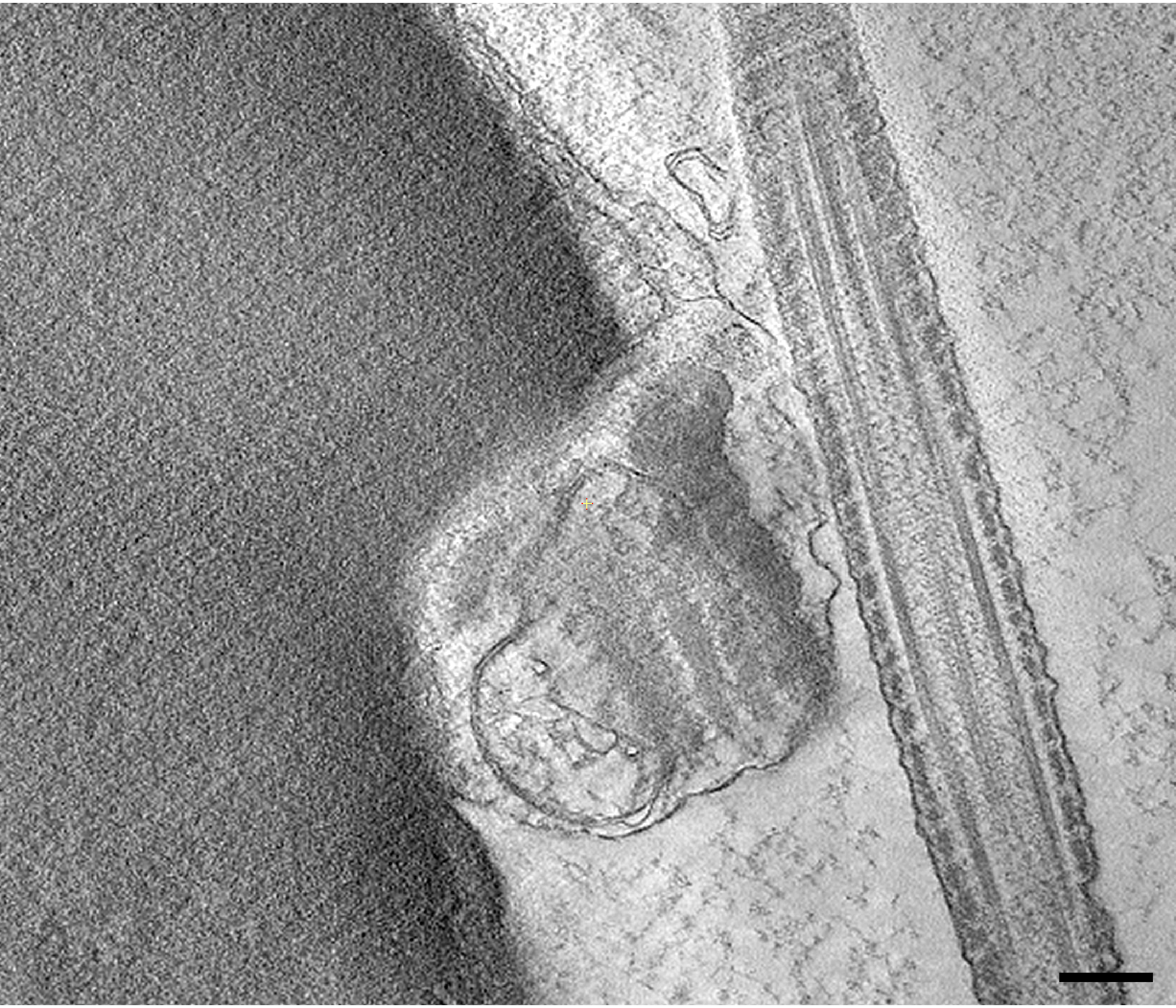

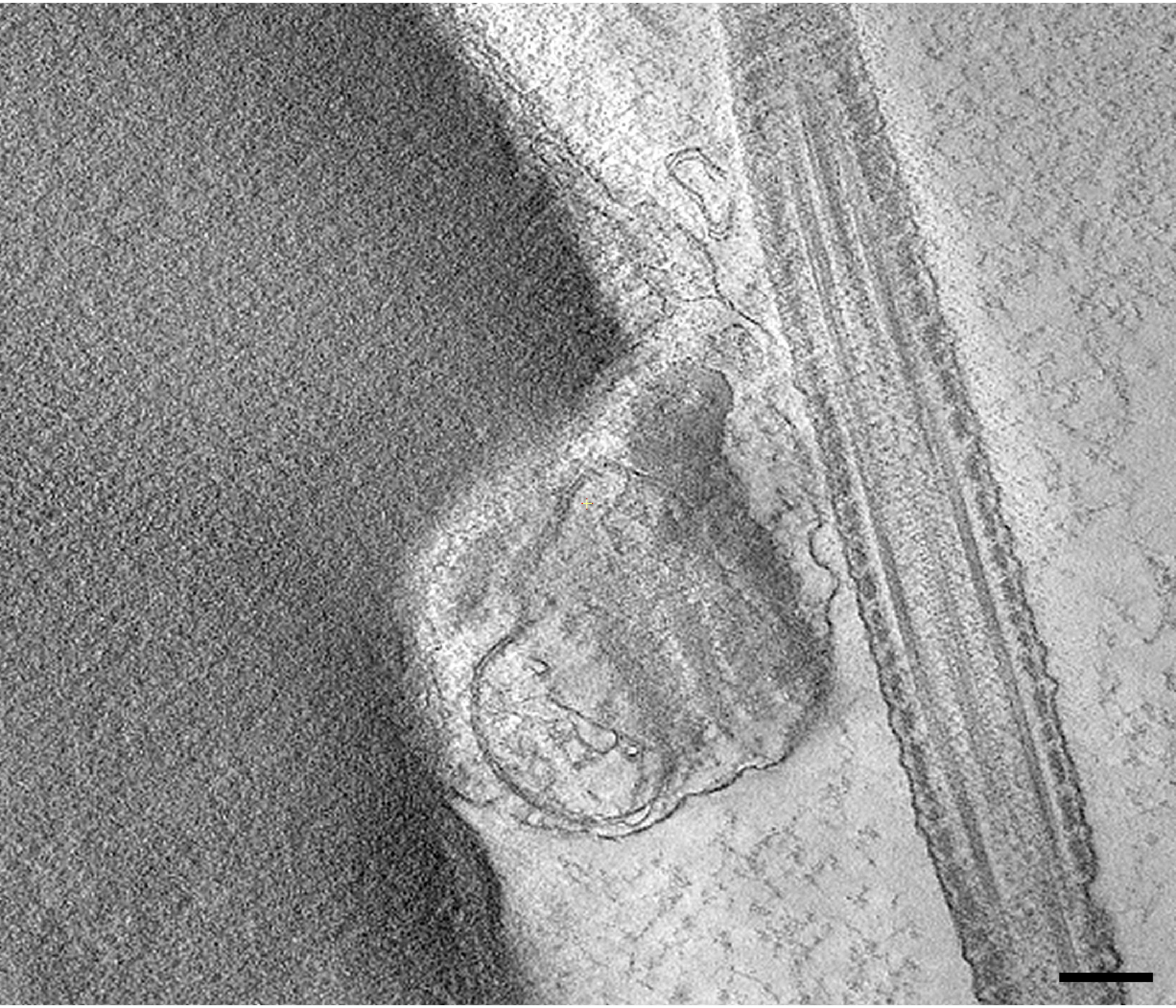

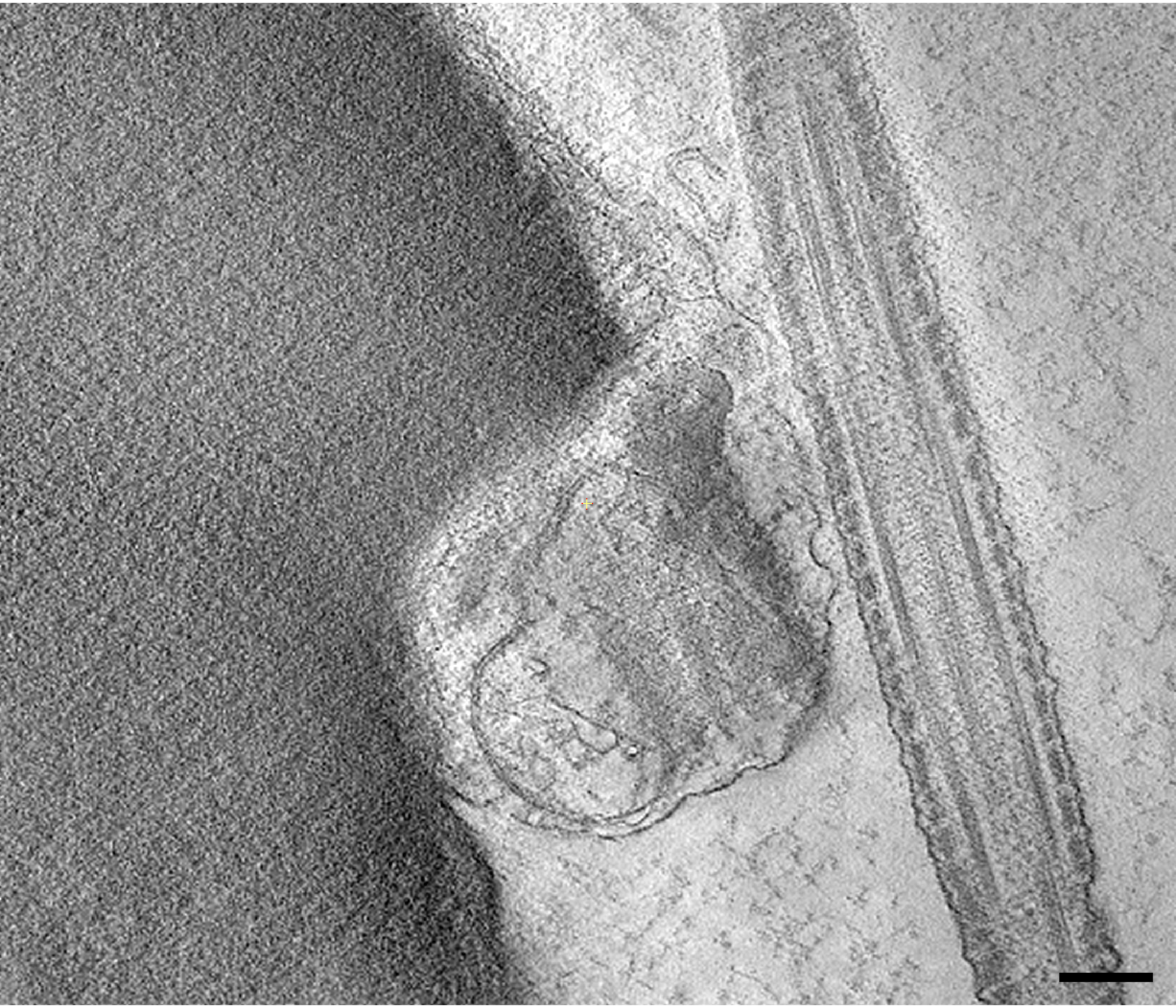

Supplement: Supplementary file 2 — SI Z stack images for Fig.6A [file 41598_2019_50516_MOESM2_ESM.pdf]
